# Supplementary figures and images for: MAF1, a repressor of RNA polymerase III-dependent transcription, regulates bone mass (part 1 of 2)
Source: eLife. 2022 May 25;11:e74740. doi: 10.7554/eLife.74740 (PMC9212997; doi:10.7554/eLife.74740)

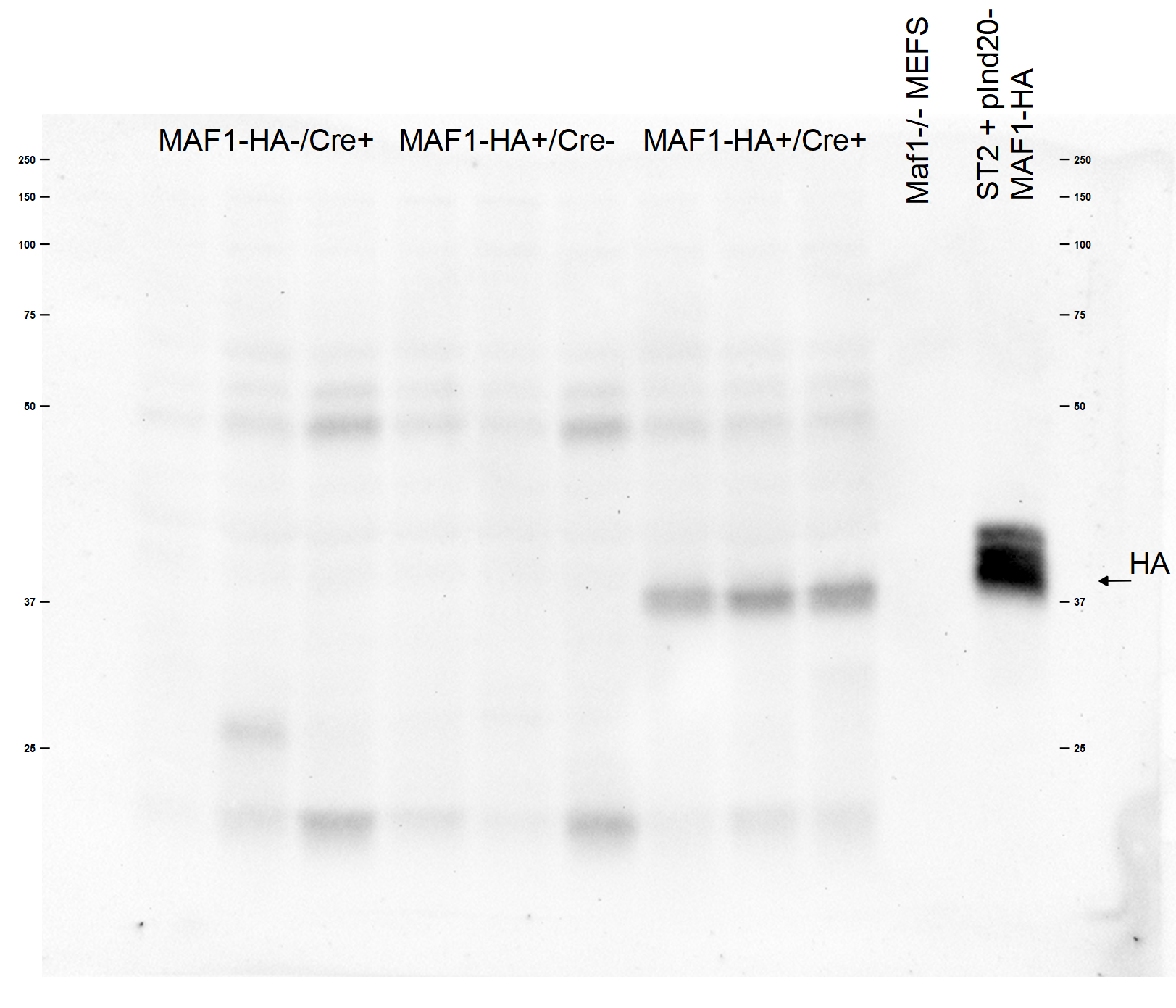

Supplement: Figure 1—source data 1. [file elife-74740-fig1-data1.zip › Figure 1- source data/Figure 1A - source data-1 HA labeled.tif]

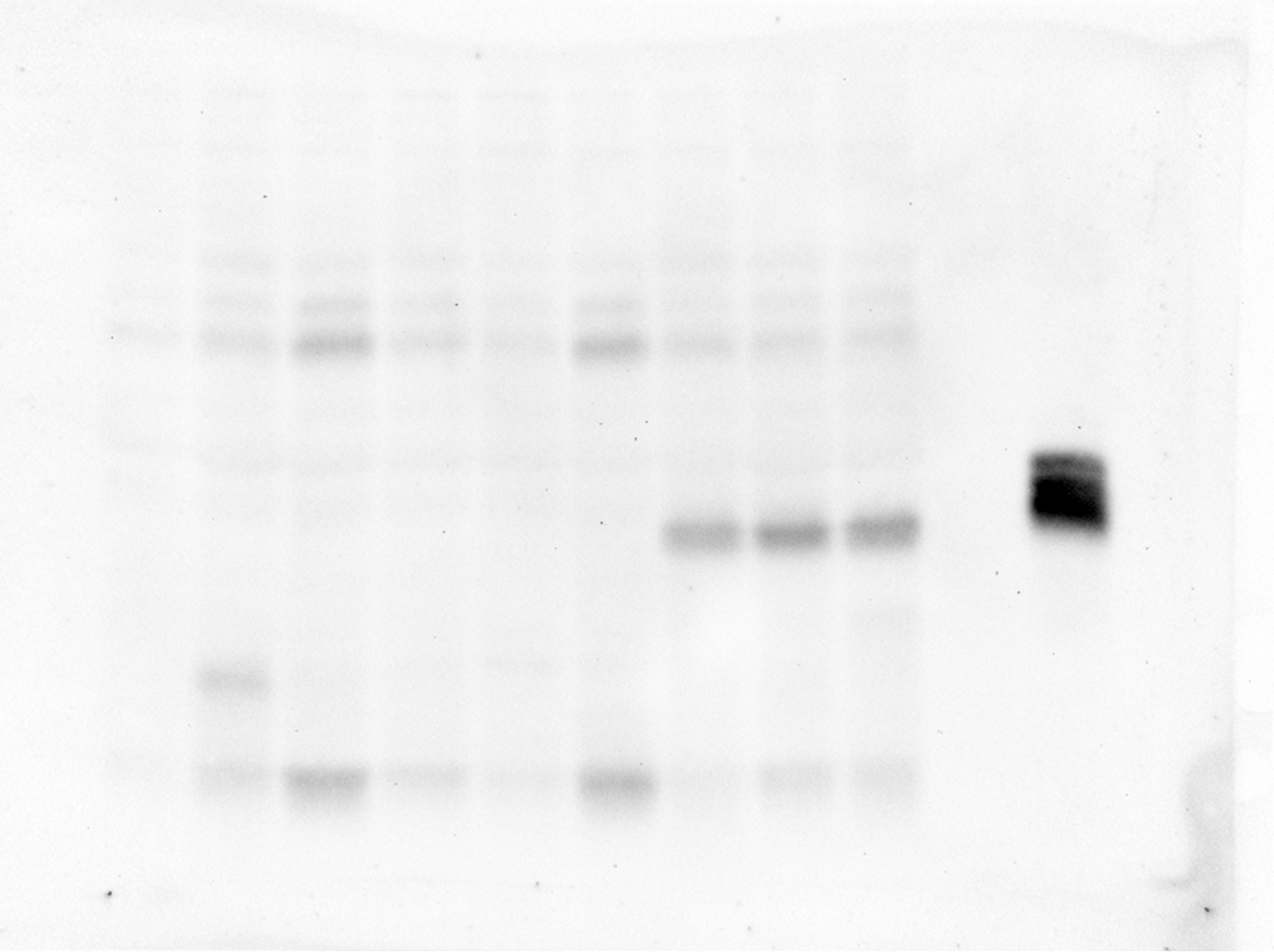

Supplement: Figure 1—source data 1. [file elife-74740-fig1-data1.zip › Figure 1- source data/Figure 1A - source data-2 HA.tif]

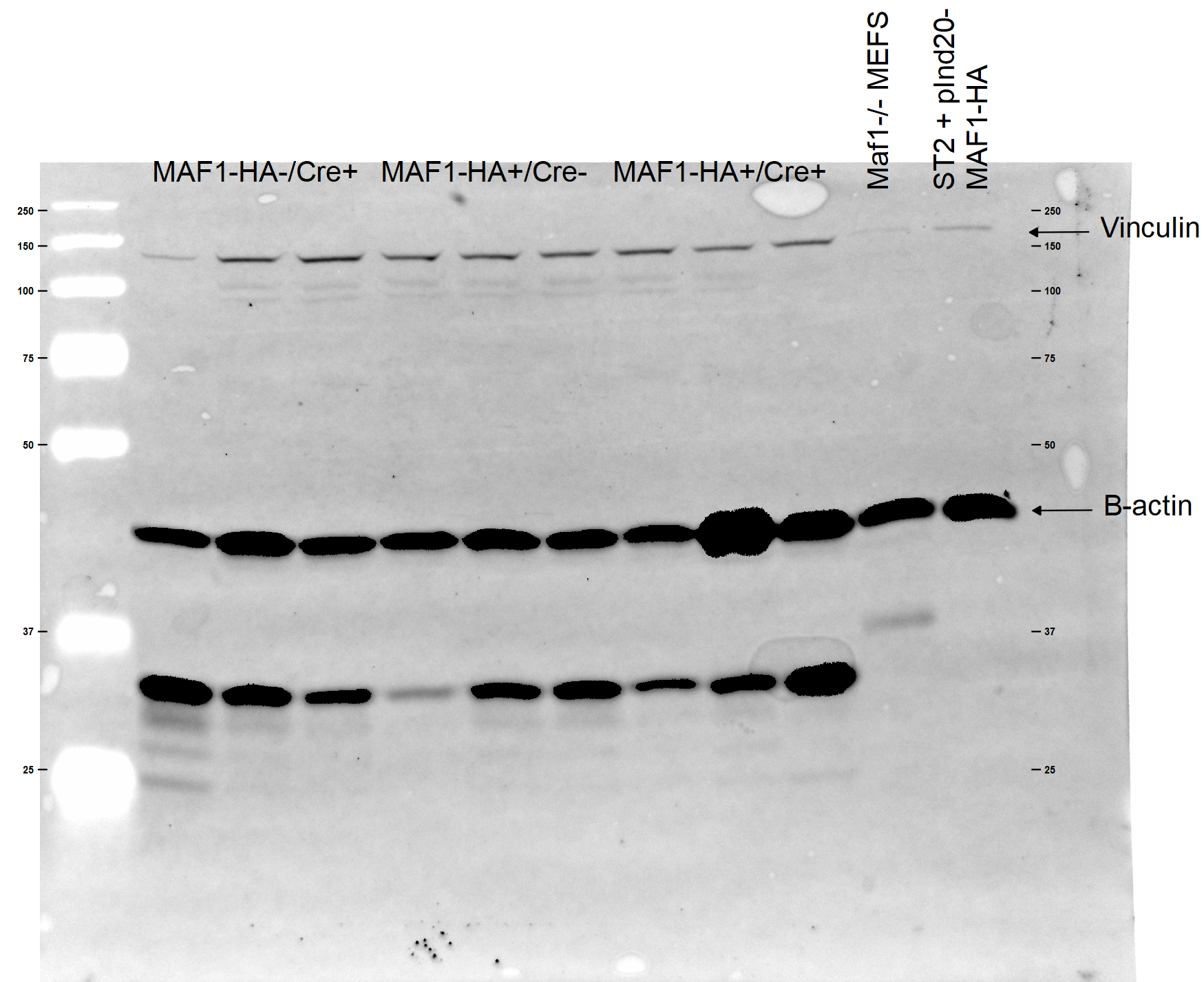

Supplement: Figure 1—source data 1. [file elife-74740-fig1-data1.zip › Figure 1- source data/Figure 1A- source data-3 vinculin labeled.tif]

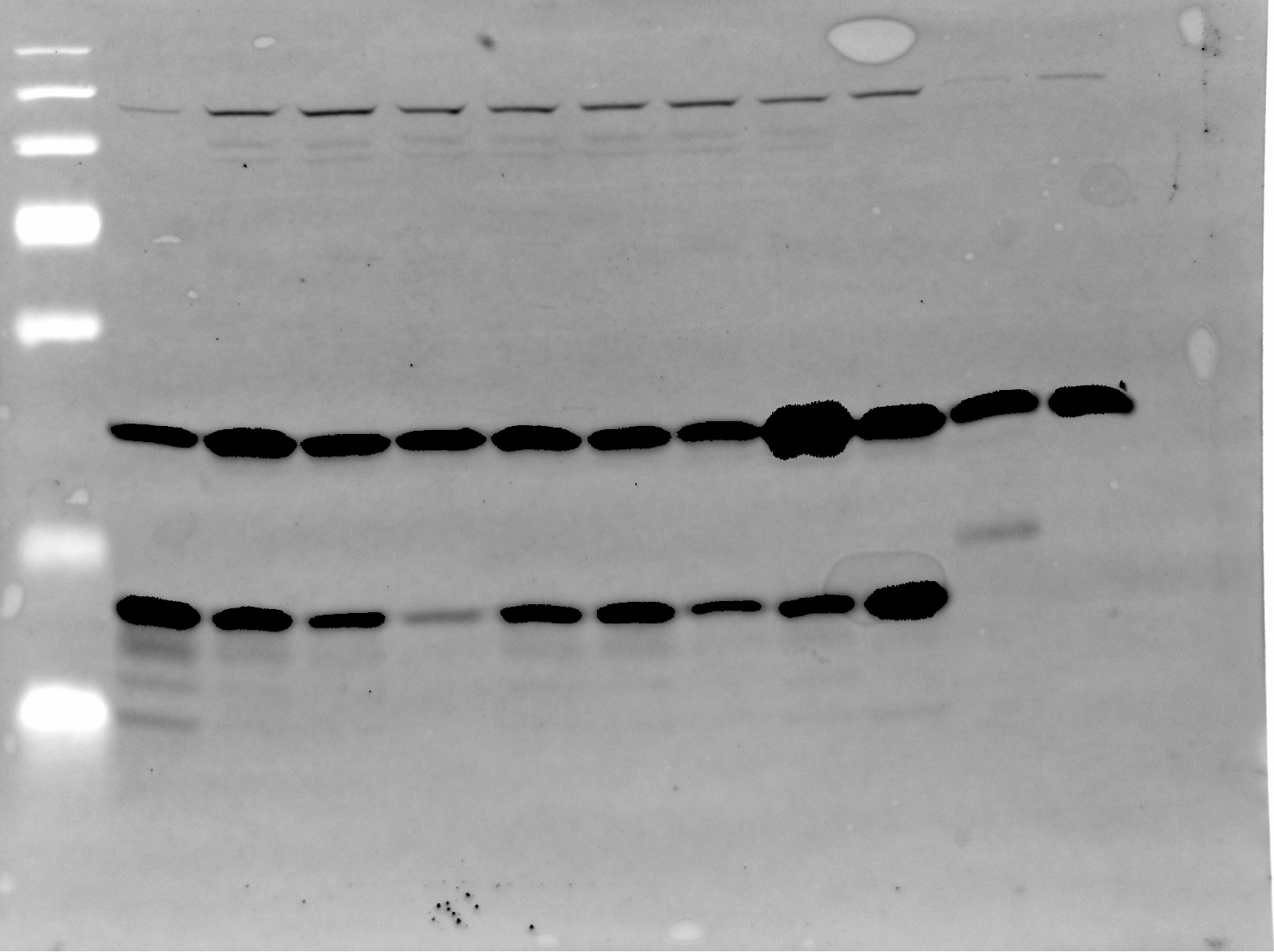

Supplement: Figure 1—source data 1. [file elife-74740-fig1-data1.zip › Figure 1- source data/Figure 1A- source data-4 vinculin.tif]

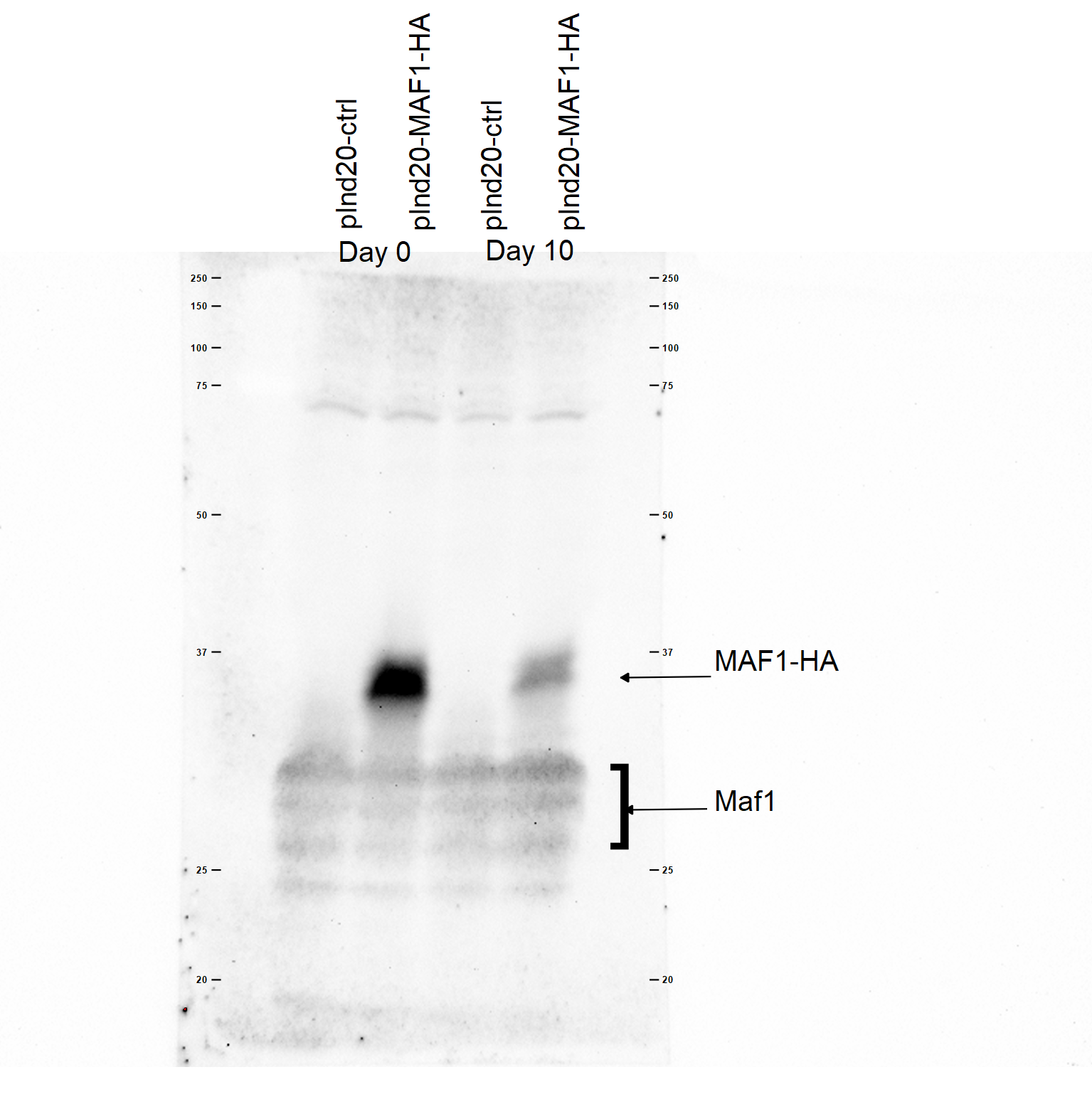

Supplement: Figure 2—source data 1. [file elife-74740-fig2-data1.zip › Figure 2A -source data/figure 2A - source data 1 Maf1 labeled.tif]

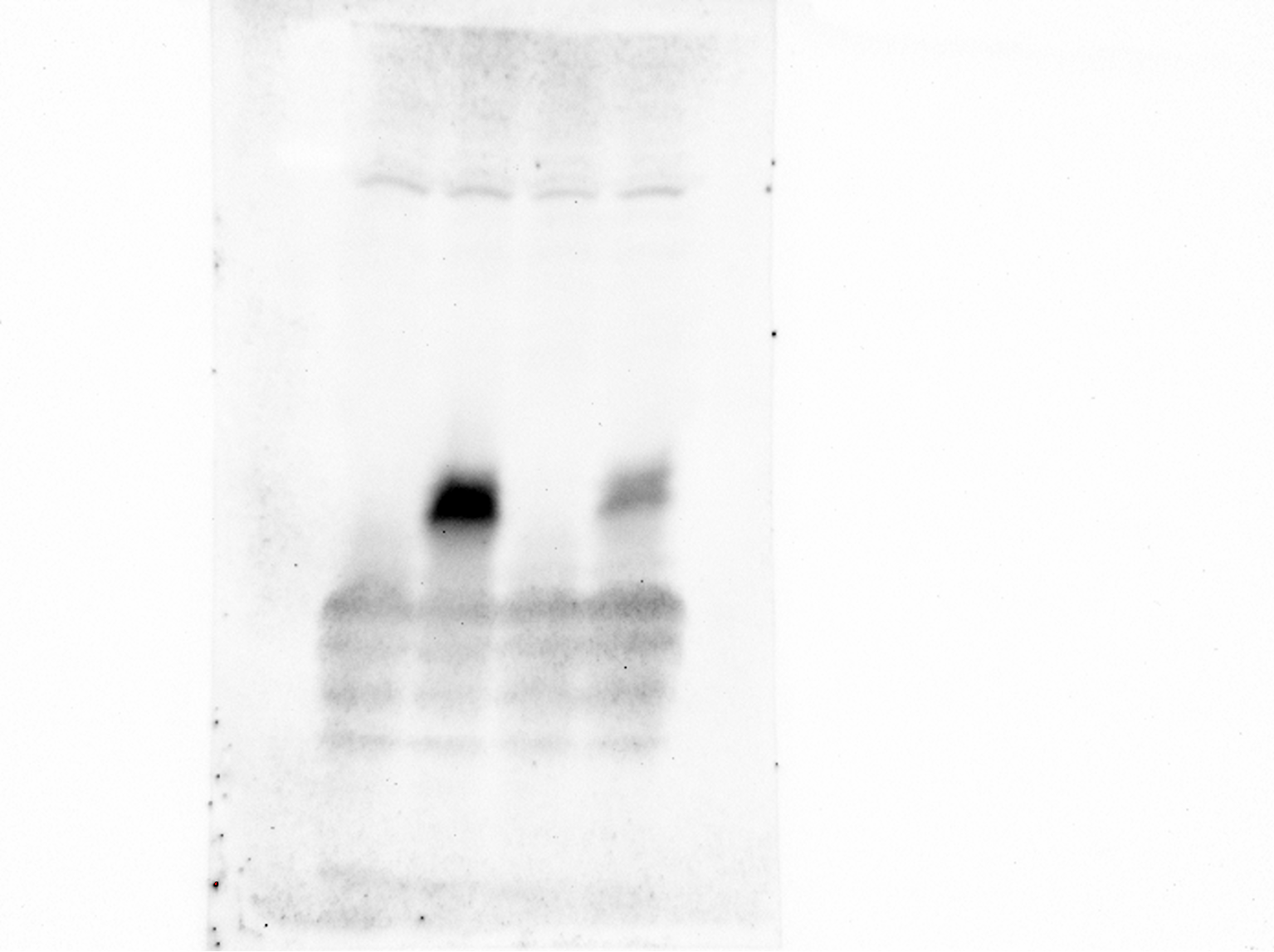

Supplement: Figure 2—source data 1. [file elife-74740-fig2-data1.zip › Figure 2A -source data/figure 2A - source data 2 Maf1.tif]

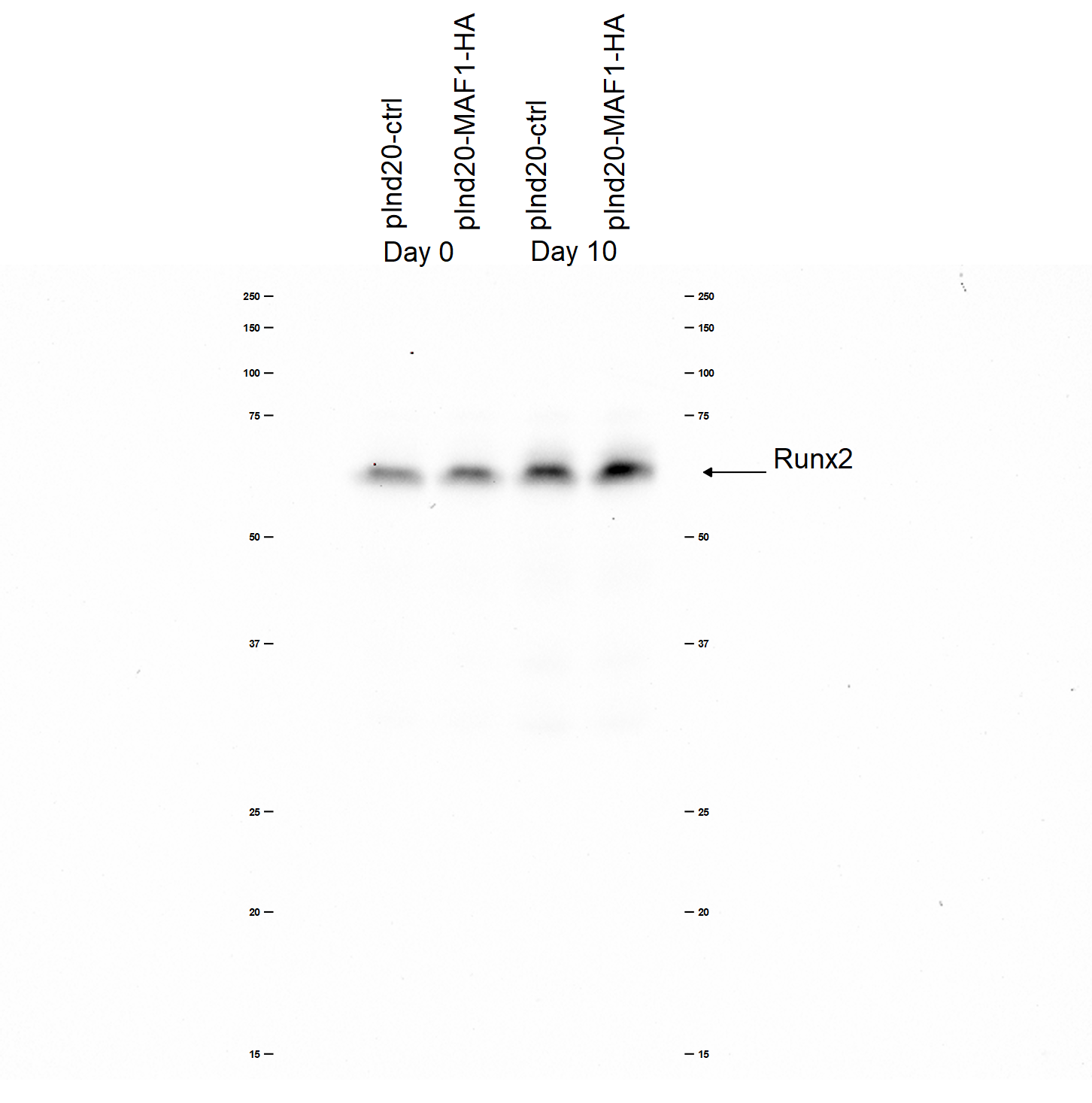

Supplement: Figure 2—source data 1. [file elife-74740-fig2-data1.zip › Figure 2A -source data/figure 2A - source data 3 Runx2 labeled.tif]

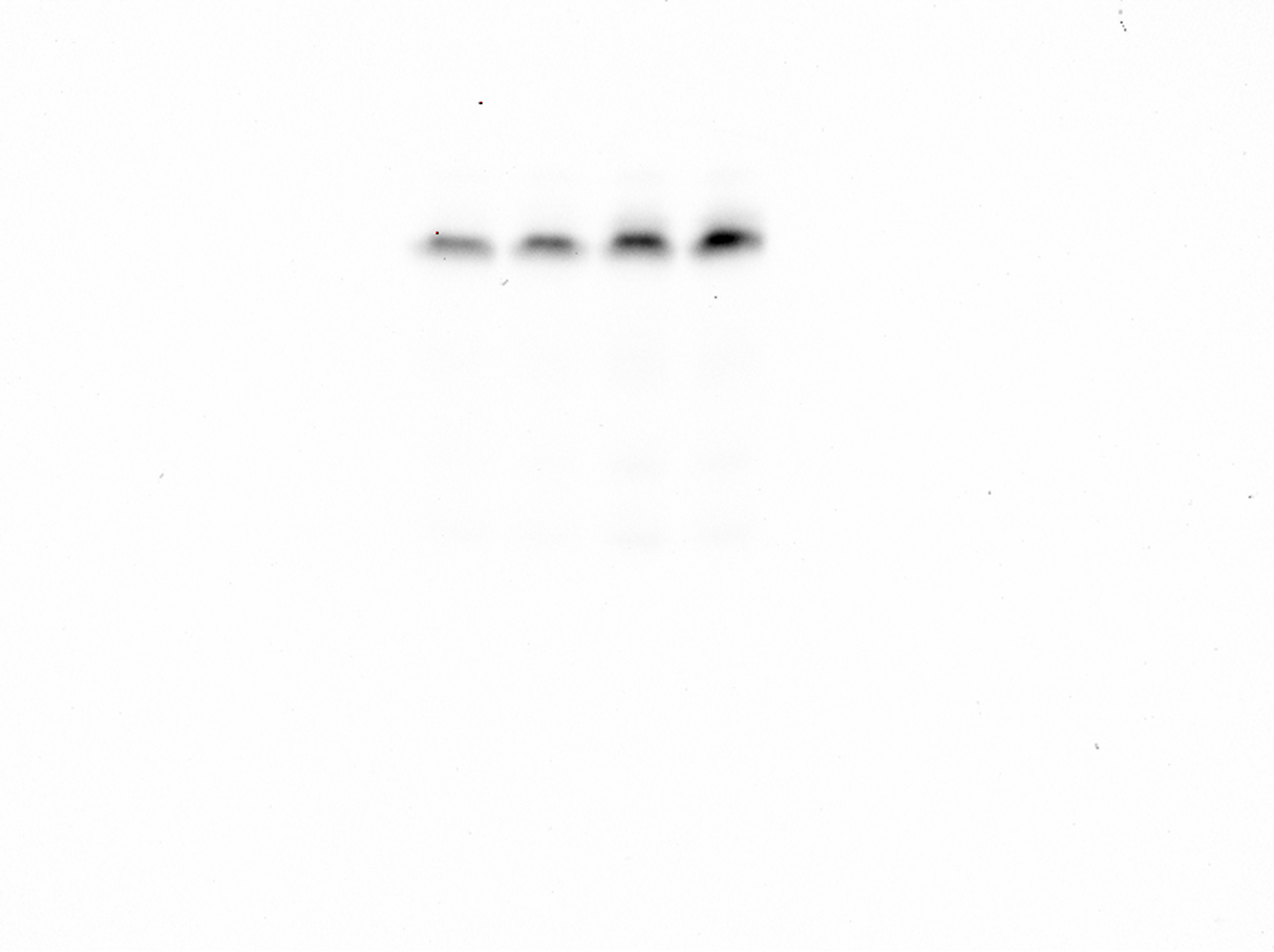

Supplement: Figure 2—source data 1. [file elife-74740-fig2-data1.zip › Figure 2A -source data/figure 2A - source data 4 Runx2.tif]

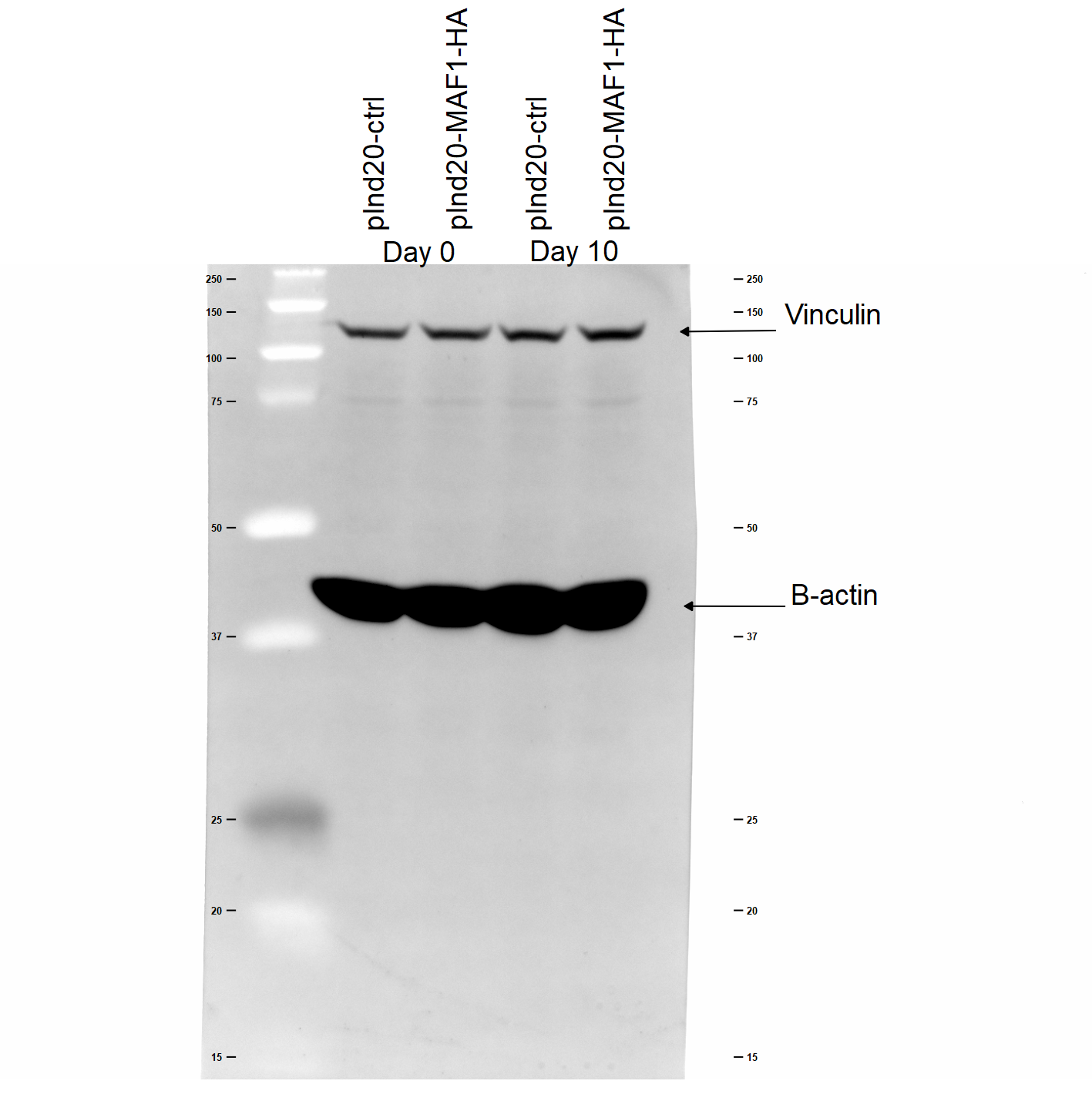

Supplement: Figure 2—source data 1. [file elife-74740-fig2-data1.zip › Figure 2A -source data/figure 2A - source data 5 Vinculin labeled.tif]

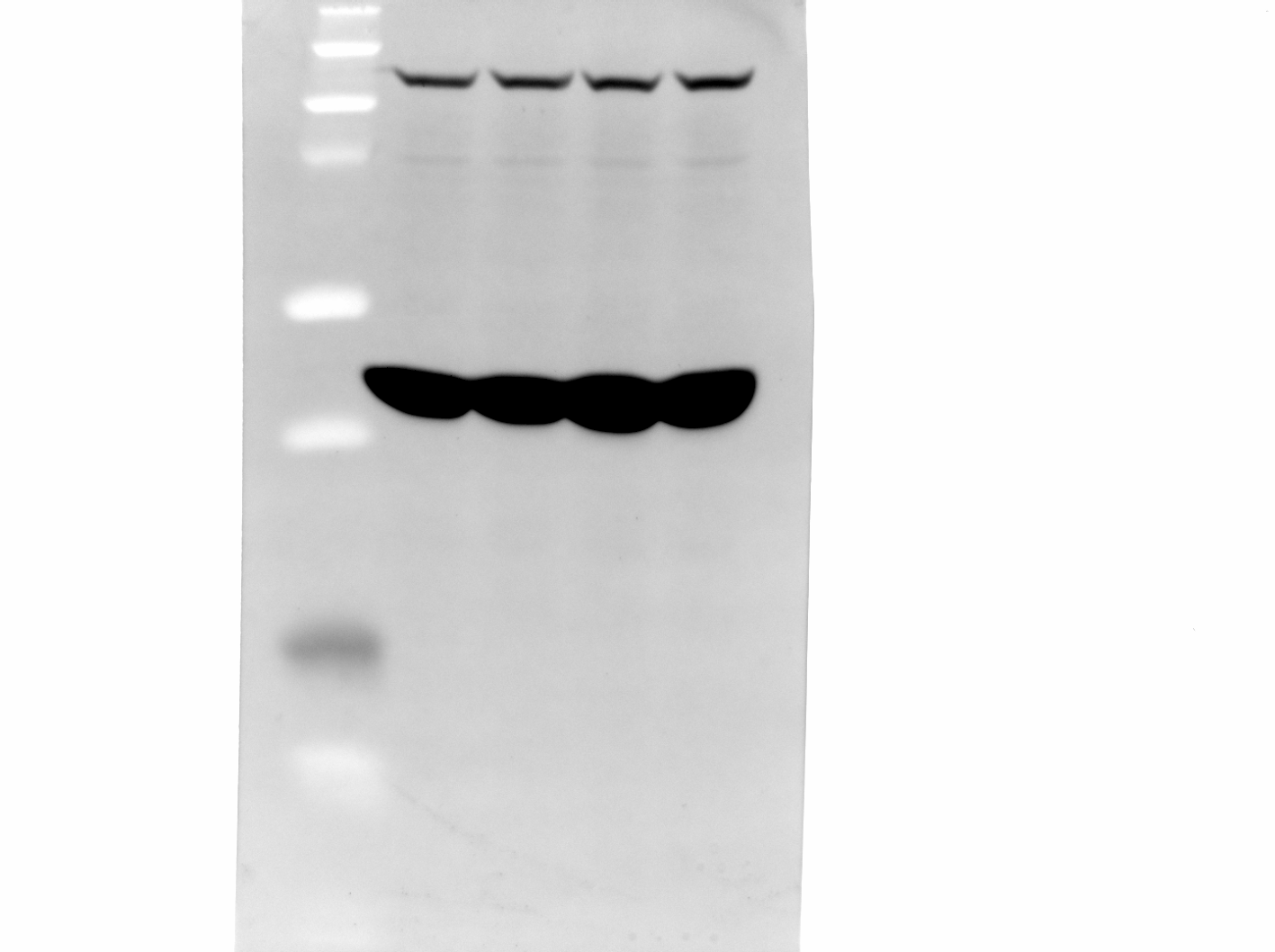

Supplement: Figure 2—source data 1. [file elife-74740-fig2-data1.zip › Figure 2A -source data/figure 2A - source data 6 Vinculin.tif]

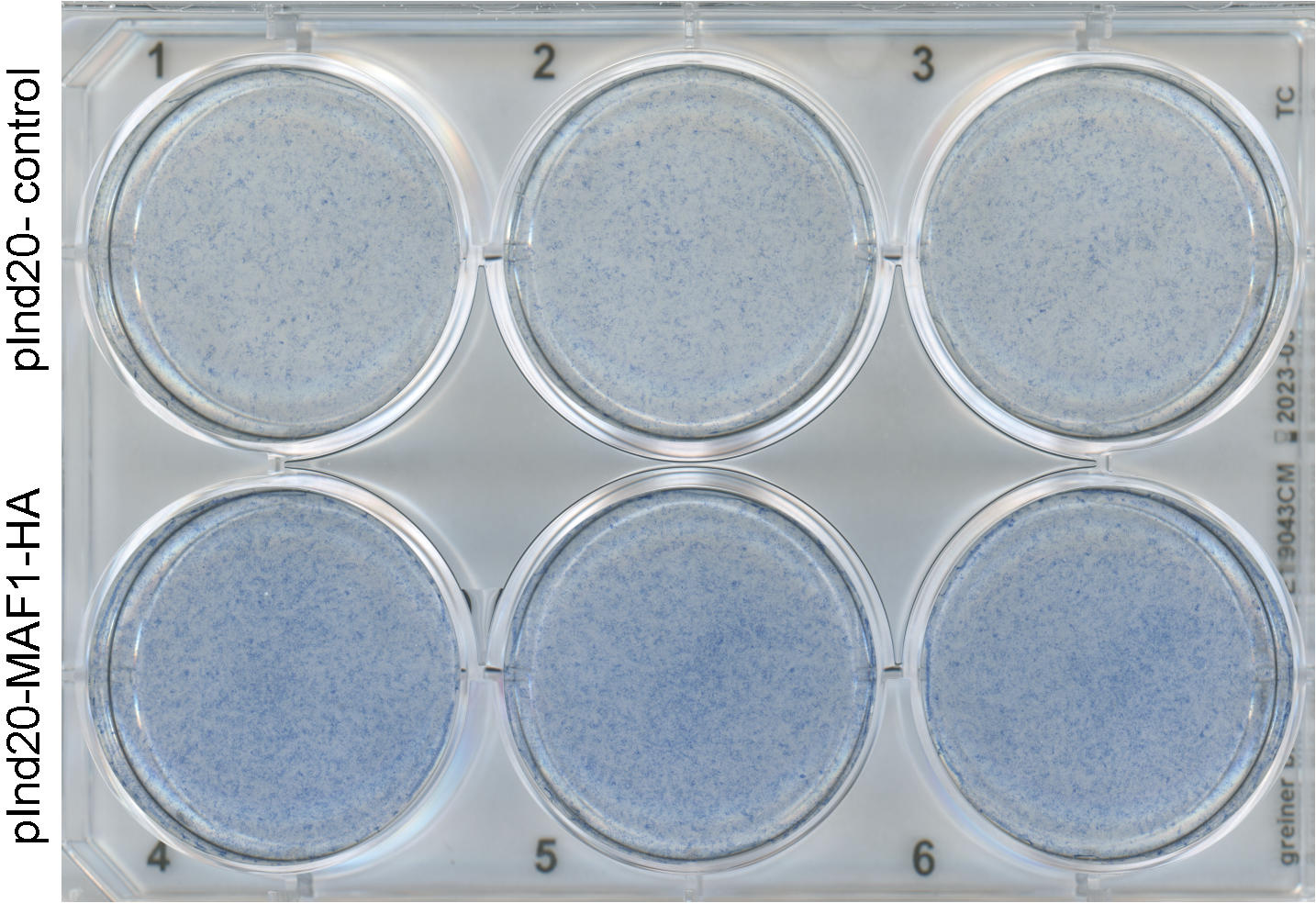

Supplement: Figure 2—source data 2. [file elife-74740-fig2-data2.zip › Figure 2 C-D -source data/Figure 2C - source data1 - ALP.png]

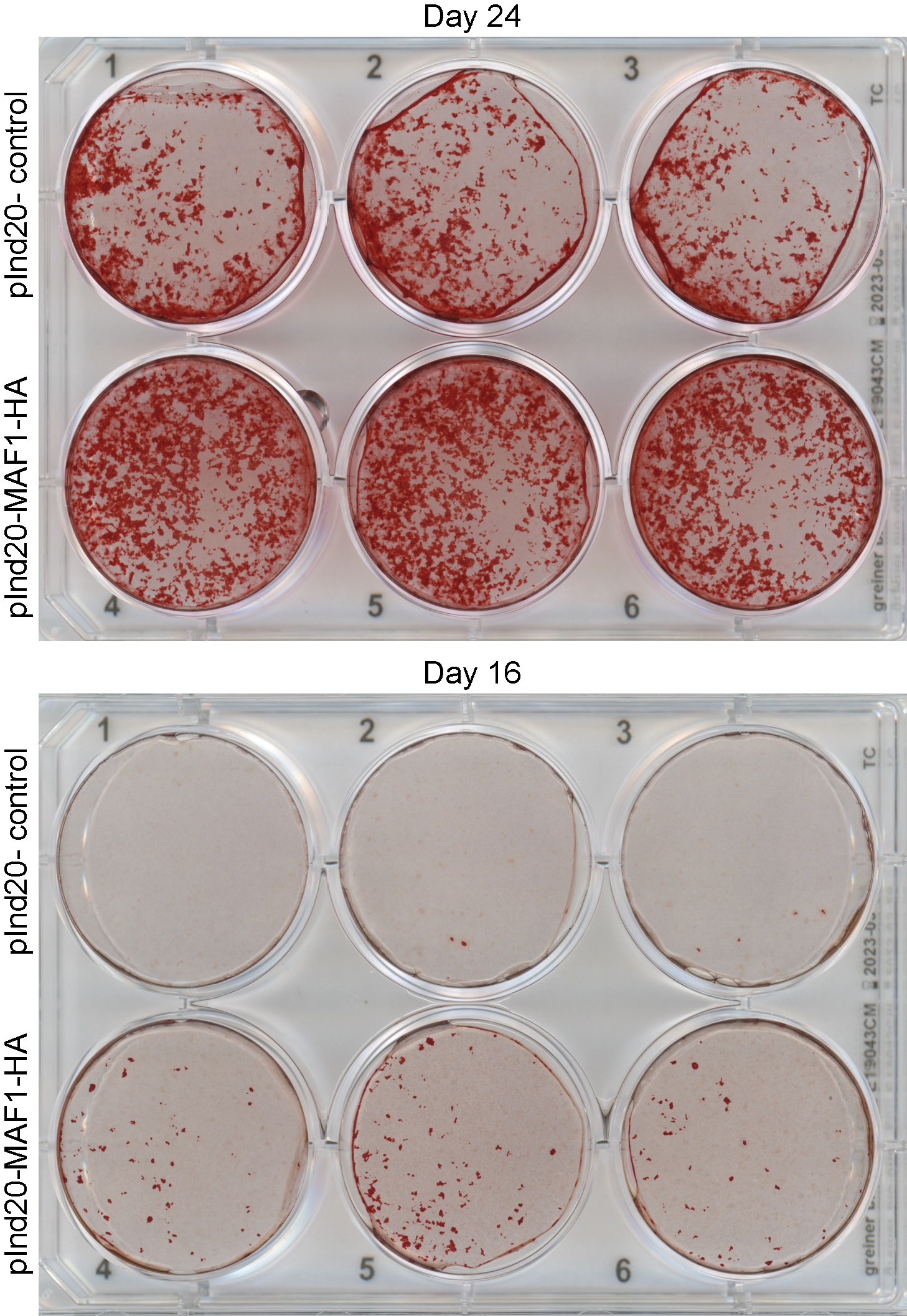

Supplement: Figure 2—source data 2. [file elife-74740-fig2-data2.zip › Figure 2 C-D -source data/Figure 2D - source data2- Alizarin Red.png]

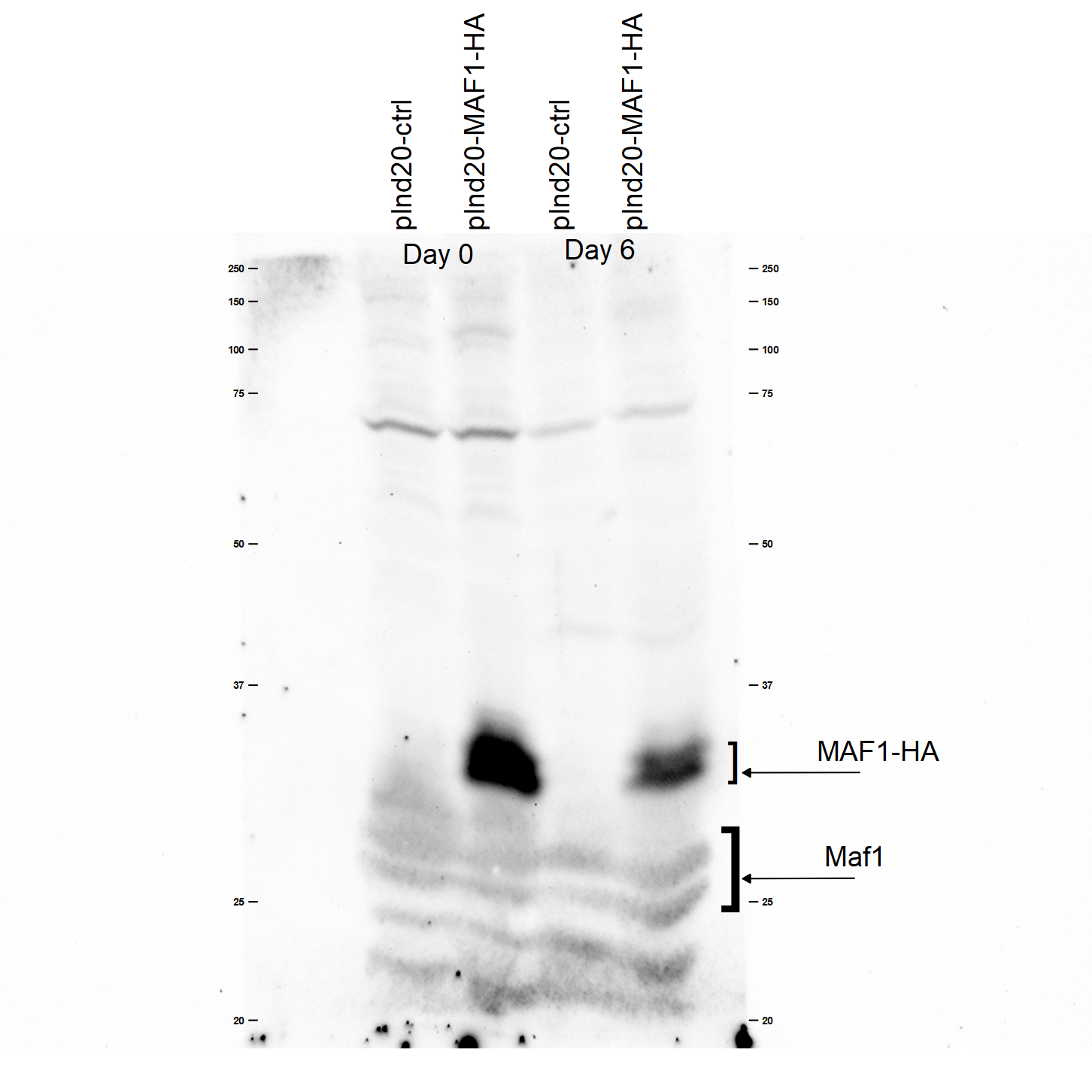

Supplement: Figure 2—figure supplement 1—source data 1. [file elife-74740-fig2-figsupp1-data1.zip › supplementary figure 5 A - source data/supplemental figure 5A source data 1 Maf1 labeled.tif]

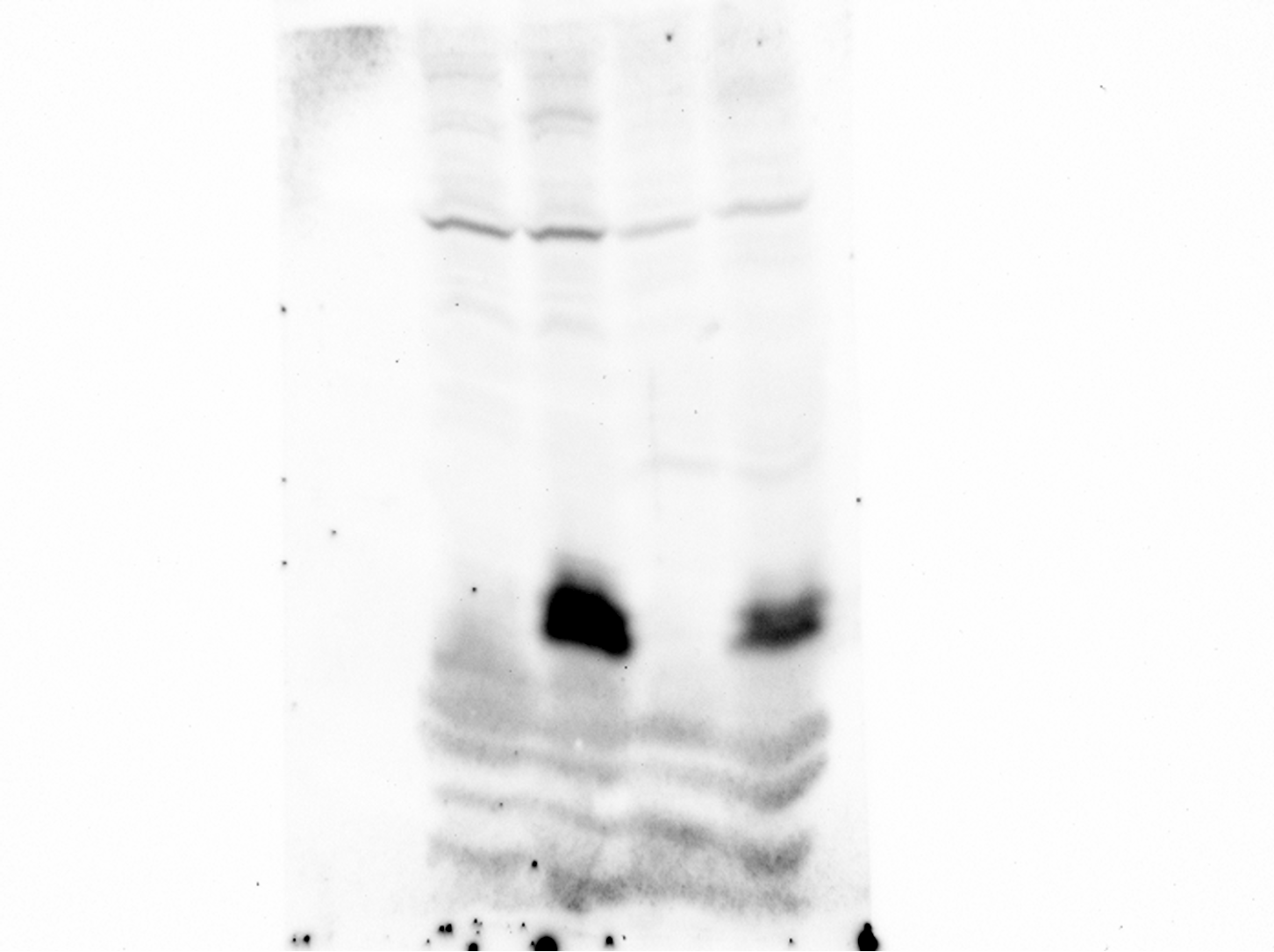

Supplement: Figure 2—figure supplement 1—source data 1. [file elife-74740-fig2-figsupp1-data1.zip › supplementary figure 5 A - source data/supplemental figure 5A source data 2 Maf1.tif]

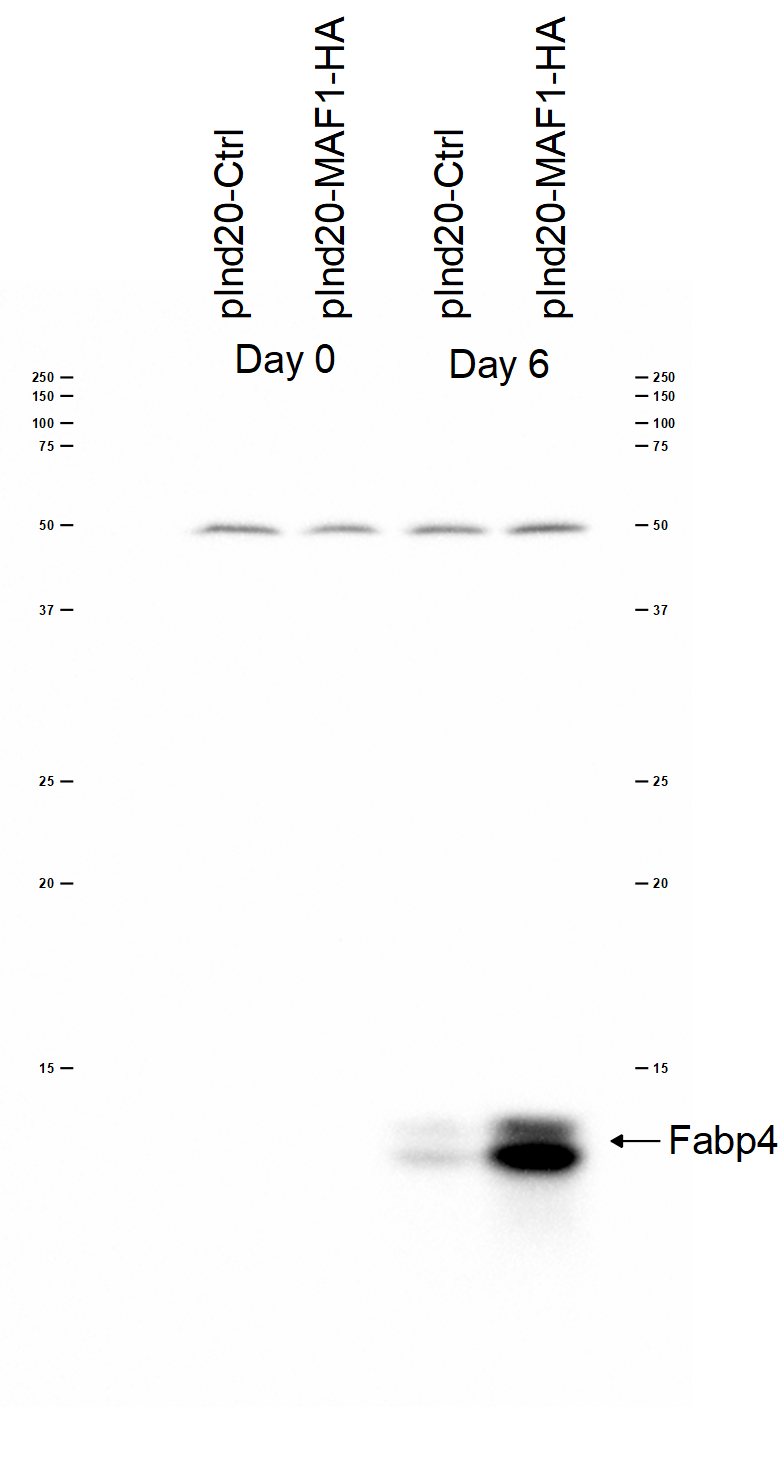

Supplement: Figure 2—figure supplement 1—source data 1. [file elife-74740-fig2-figsupp1-data1.zip › supplementary figure 5 A - source data/supplemental figure 5A source data 3 Fabp4 labeled.tif]

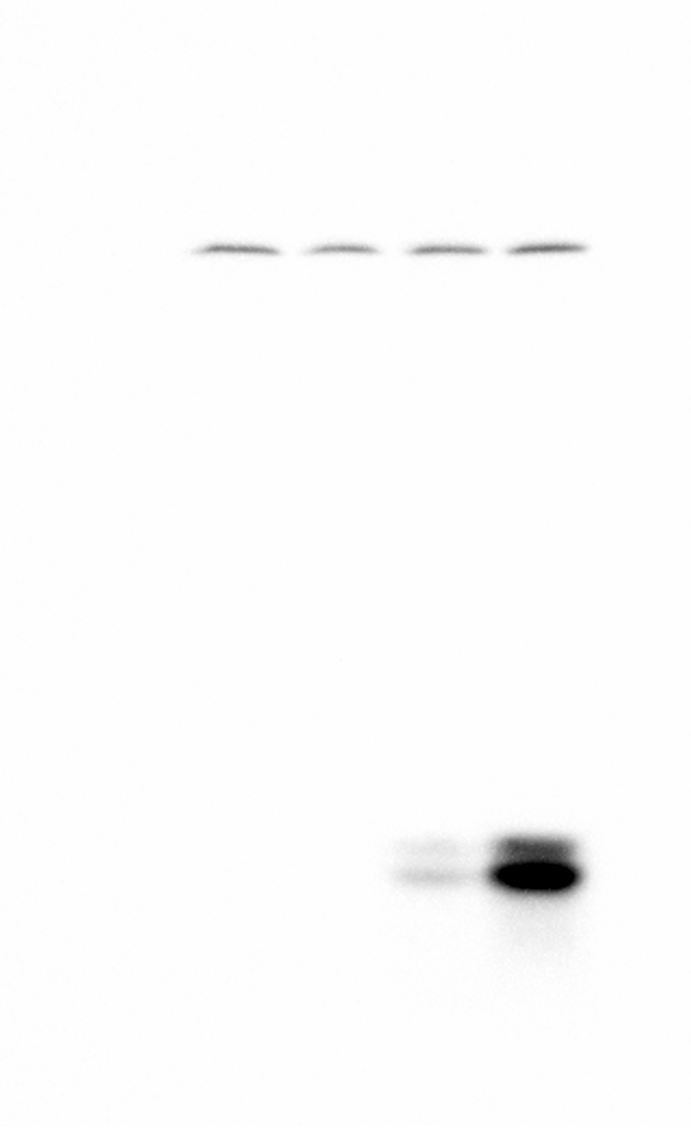

Supplement: Figure 2—figure supplement 1—source data 1. [file elife-74740-fig2-figsupp1-data1.zip › supplementary figure 5 A - source data/supplemental figure 5A source data 4 Fabp4.tif]

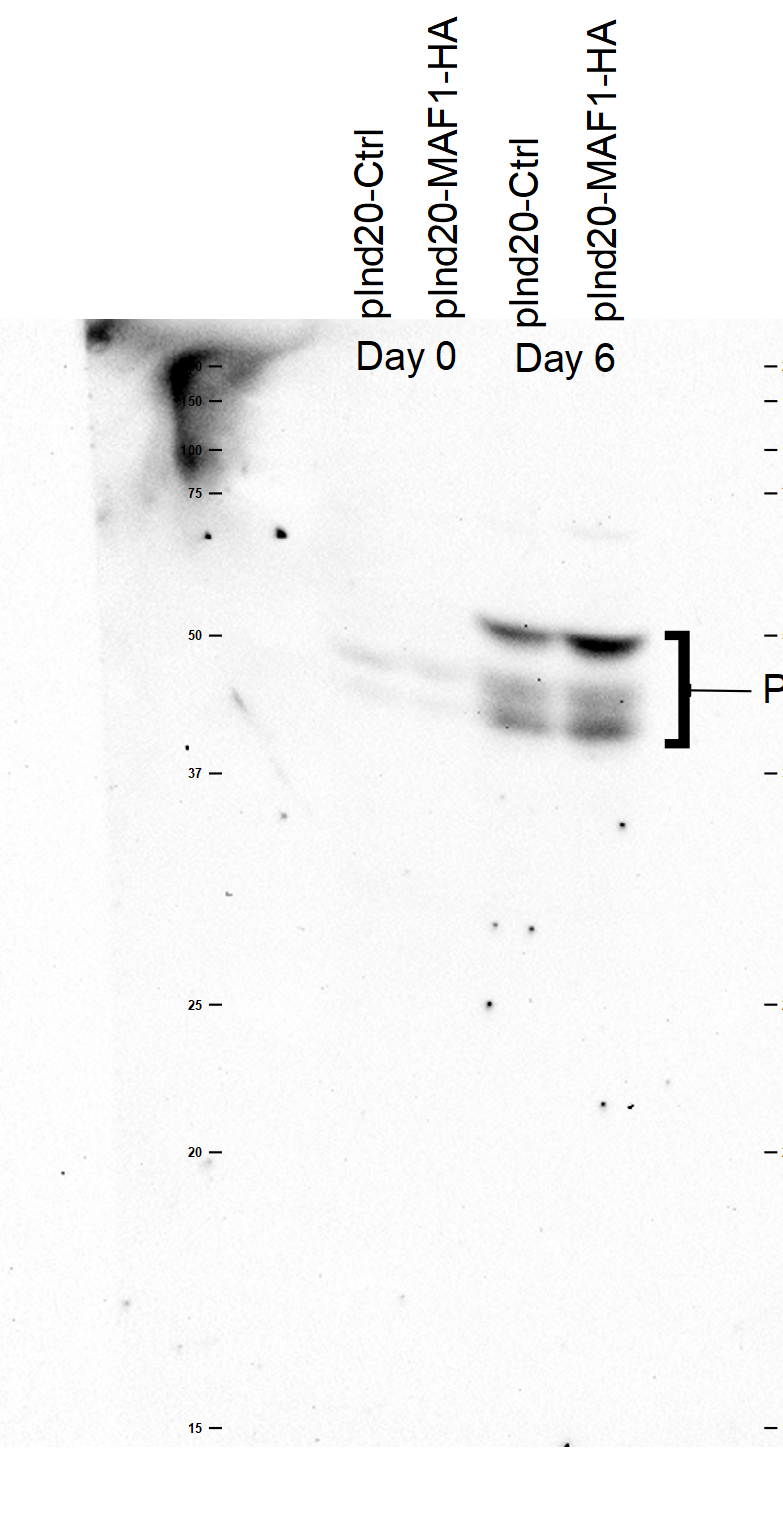

Supplement: Figure 2—figure supplement 1—source data 1. [file elife-74740-fig2-figsupp1-data1.zip › supplementary figure 5 A - source data/supplemental figure 5A source data 5 Ppary labeled.tif]

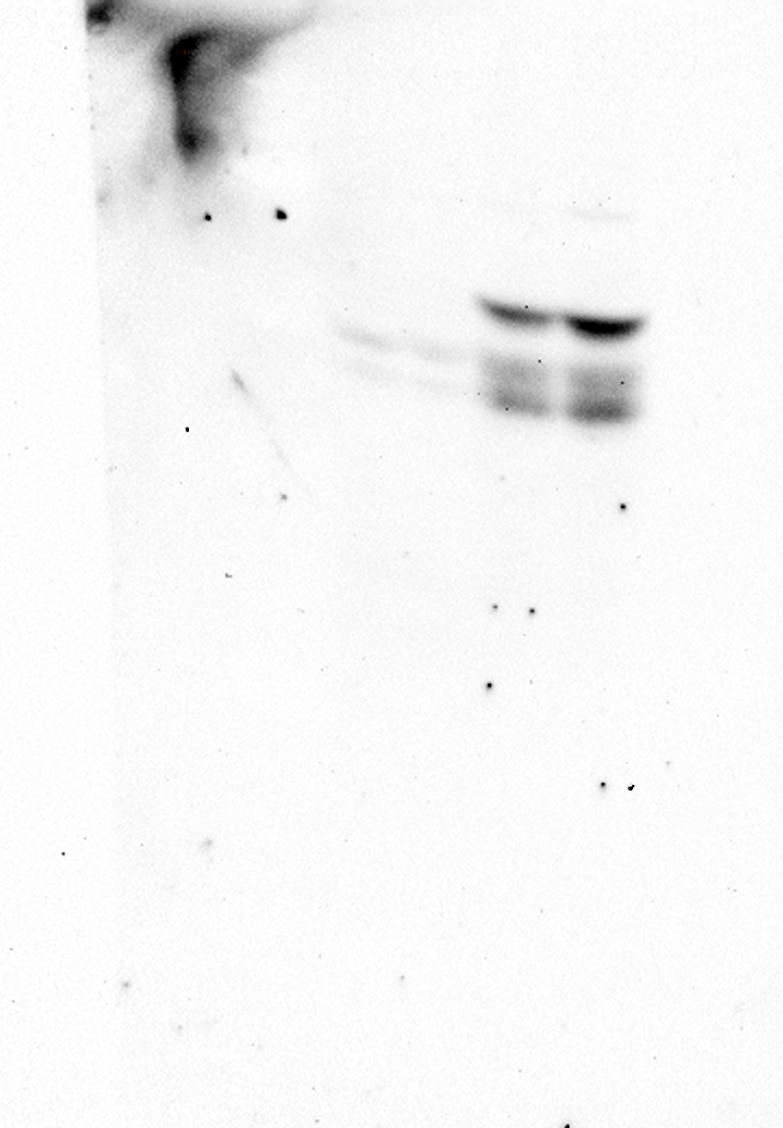

Supplement: Figure 2—figure supplement 1—source data 1. [file elife-74740-fig2-figsupp1-data1.zip › supplementary figure 5 A - source data/supplemental figure 5A source data 6 PPARy.tif]

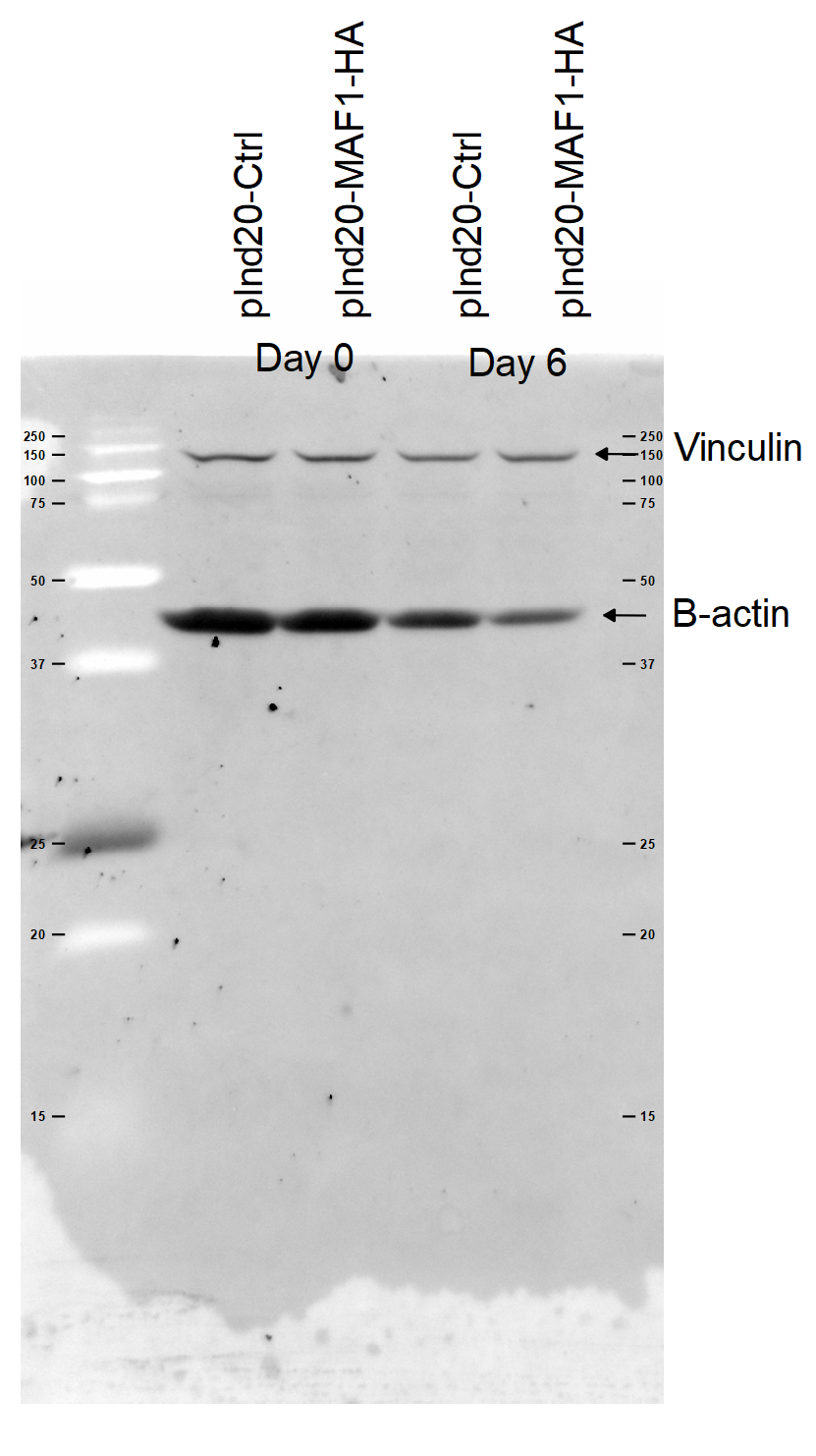

Supplement: Figure 2—figure supplement 1—source data 1. [file elife-74740-fig2-figsupp1-data1.zip › supplementary figure 5 A - source data/supplemental figure 5A source data 7 Vinculin labeled.tif]

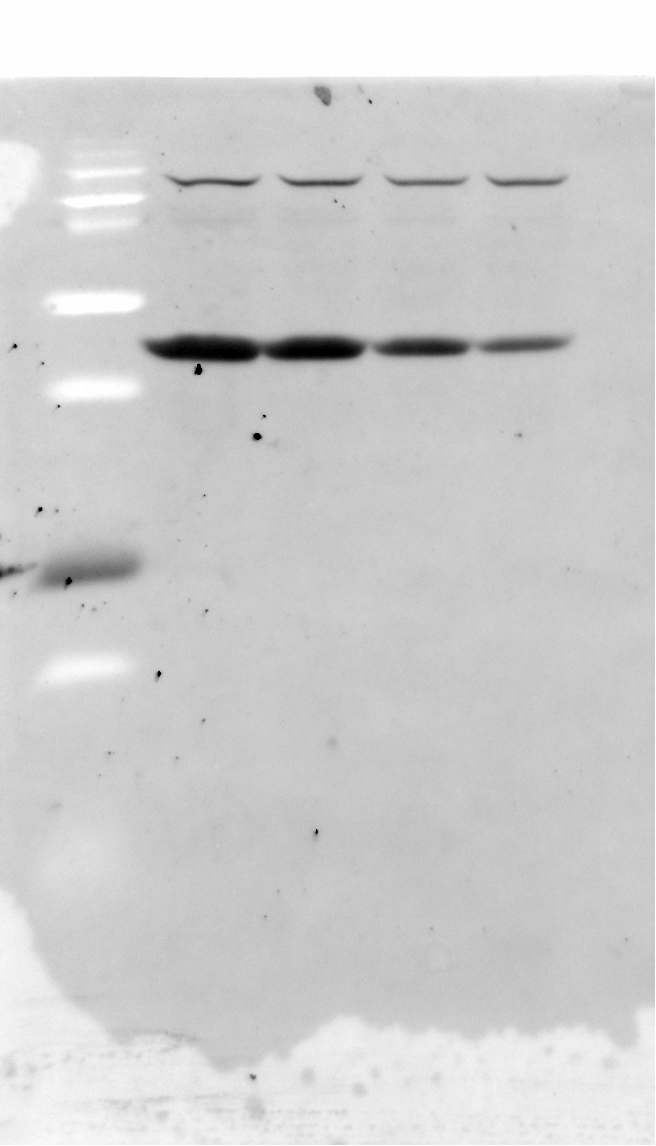

Supplement: Figure 2—figure supplement 1—source data 1. [file elife-74740-fig2-figsupp1-data1.zip › supplementary figure 5 A - source data/supplemental figure 5A source data 8 Vinculin.tif]

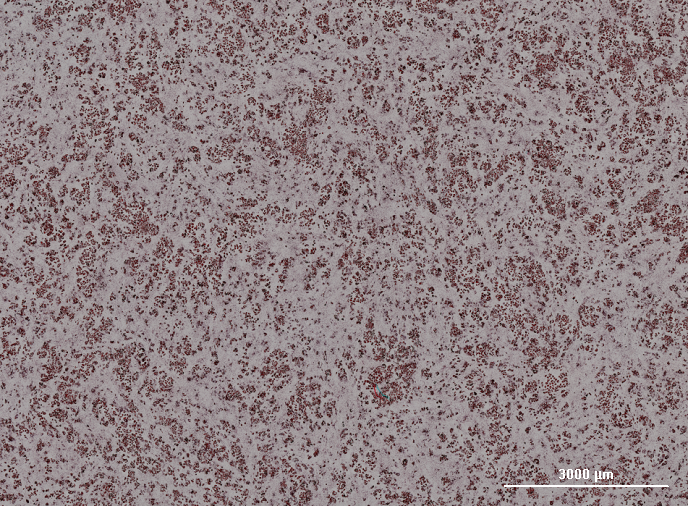

Supplement: Figure 2—figure supplement 1—source data 2. [file elife-74740-fig2-figsupp1-data2.zip › supplementary figure 5 D - source data/suppl fig 5D - source data 10 - well 3 crtl stitched.png]

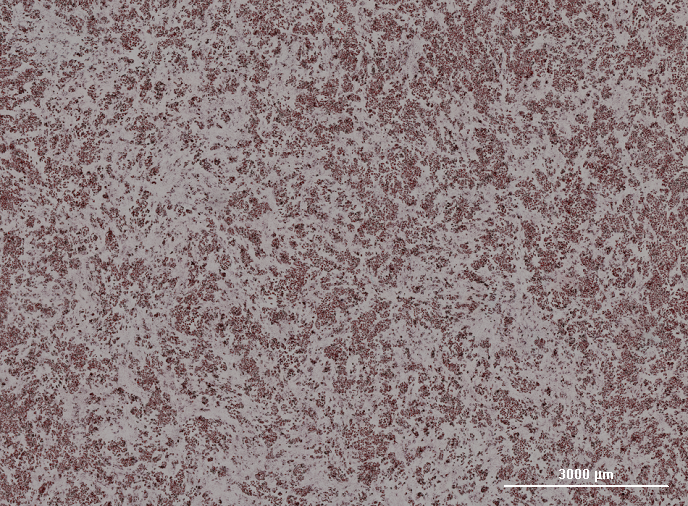

Supplement: Figure 2—figure supplement 1—source data 2. [file elife-74740-fig2-figsupp1-data2.zip › supplementary figure 5 D - source data/suppl fig 5D - source data 11 - well 4 MAF1 stitched.png]

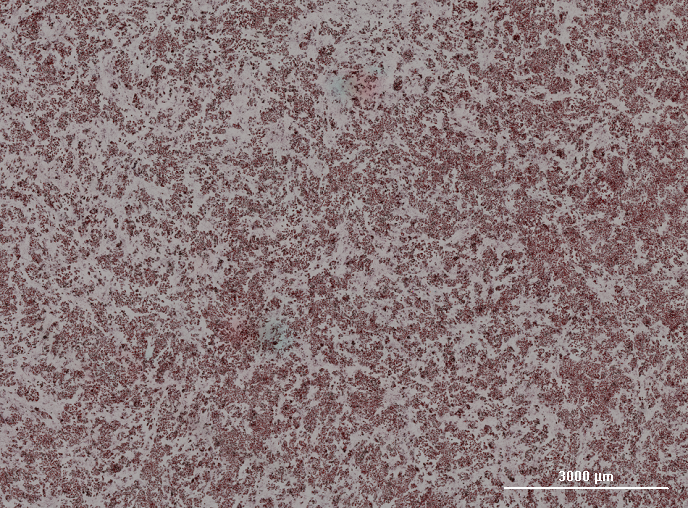

Supplement: Figure 2—figure supplement 1—source data 2. [file elife-74740-fig2-figsupp1-data2.zip › supplementary figure 5 D - source data/suppl fig 5D - source data 12 - well 5 MAF1 stitched.png]

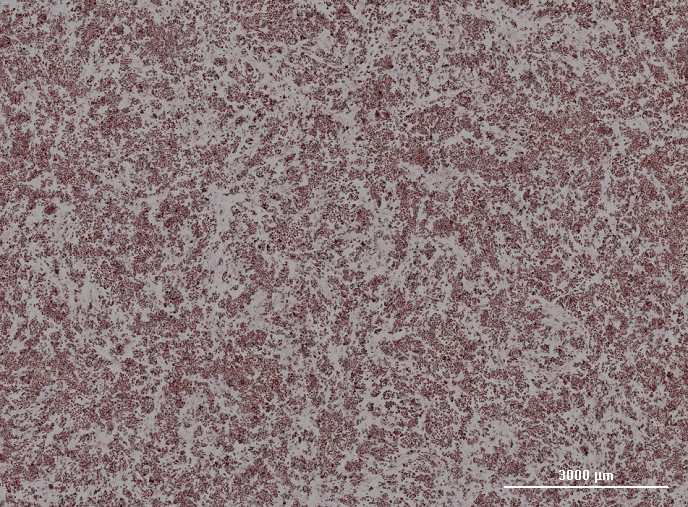

Supplement: Figure 2—figure supplement 1—source data 2. [file elife-74740-fig2-figsupp1-data2.zip › supplementary figure 5 D - source data/suppl fig 5D - source data 13 - well 6 MAF1 stitched.png]

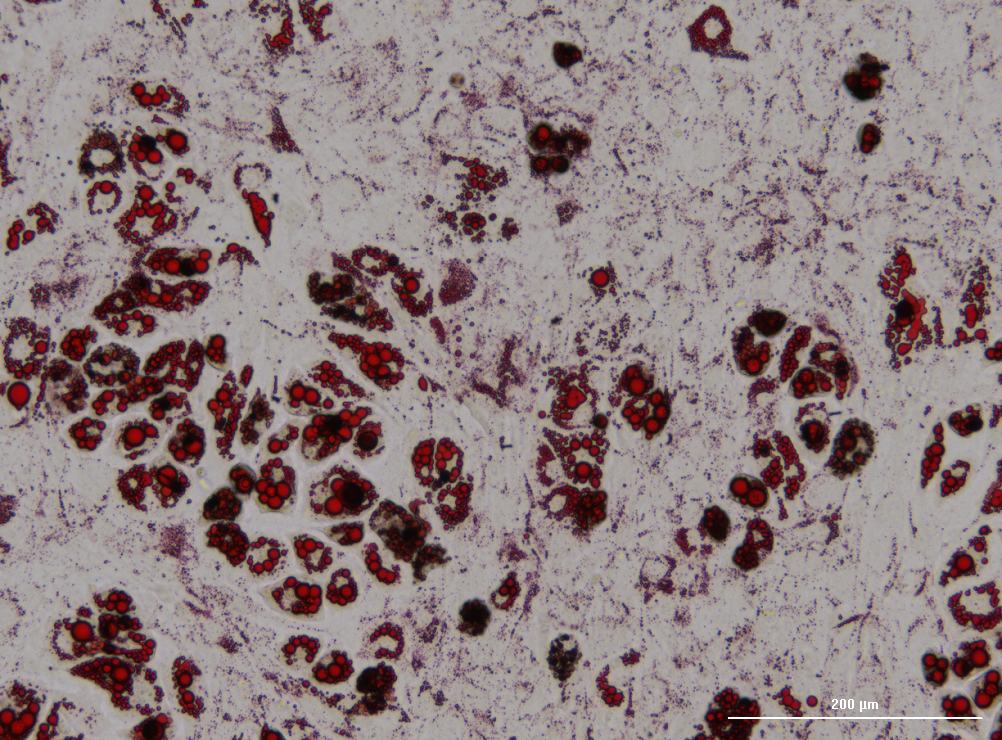

Supplement: Figure 2—figure supplement 1—source data 2. [file elife-74740-fig2-figsupp1-data2.zip › supplementary figure 5 D - source data/suppl fig 5D - source data 4 - well 3 ctrl 10x.png]

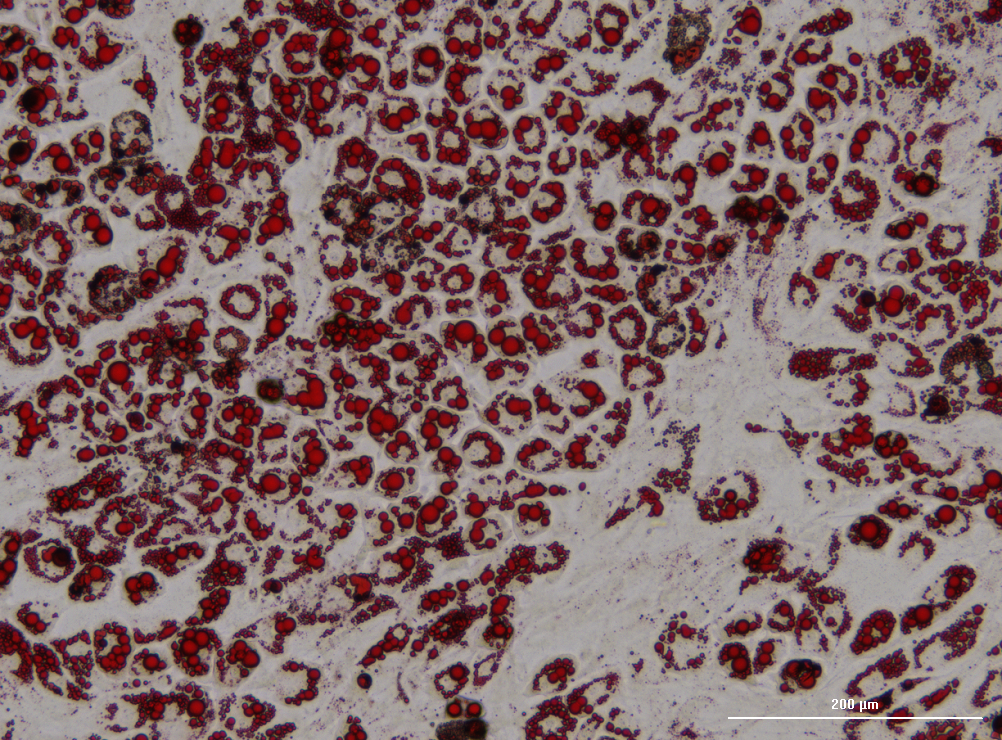

Supplement: Figure 2—figure supplement 1—source data 2. [file elife-74740-fig2-figsupp1-data2.zip › supplementary figure 5 D - source data/suppl fig 5D - source data 6 - well 5 MAF1 10x.png]

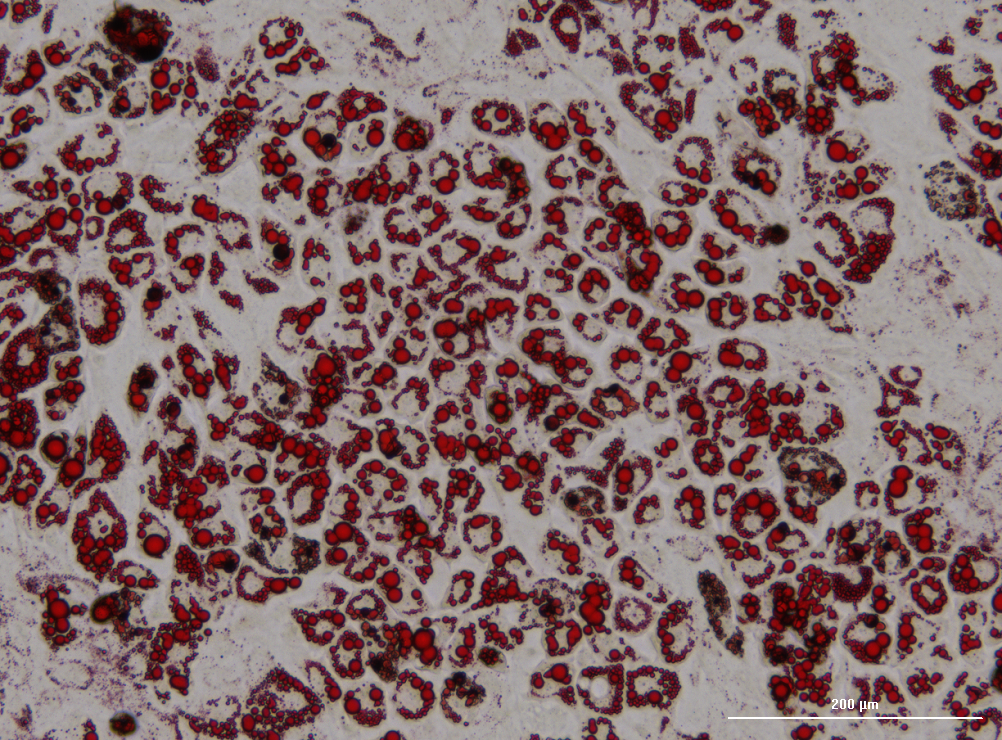

Supplement: Figure 2—figure supplement 1—source data 2. [file elife-74740-fig2-figsupp1-data2.zip › supplementary figure 5 D - source data/suppl fig 5D - source data 7 - well 6 MAF1 10X.png]

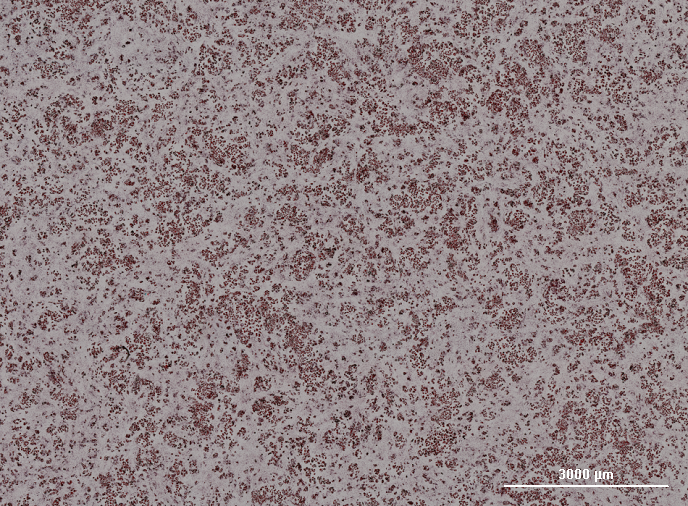

Supplement: Figure 2—figure supplement 1—source data 2. [file elife-74740-fig2-figsupp1-data2.zip › supplementary figure 5 D - source data/suppl fig 5D - source data 8 - well 1 ctrl stitched.png]

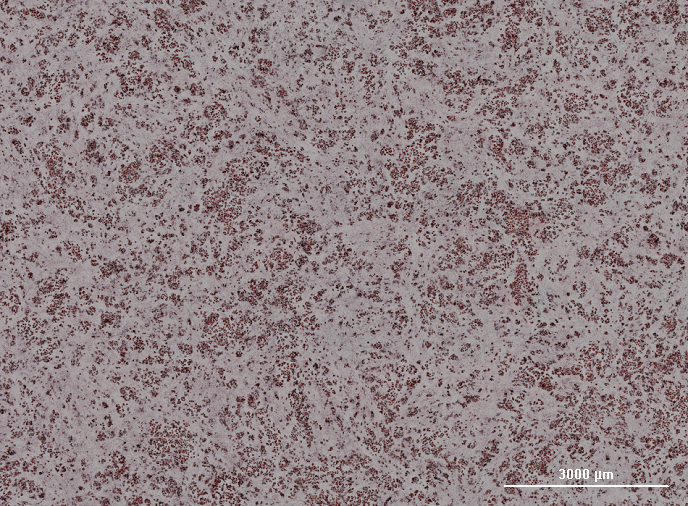

Supplement: Figure 2—figure supplement 1—source data 2. [file elife-74740-fig2-figsupp1-data2.zip › supplementary figure 5 D - source data/suppl fig 5D - source data 9 - well 2 crtl stitched.png]

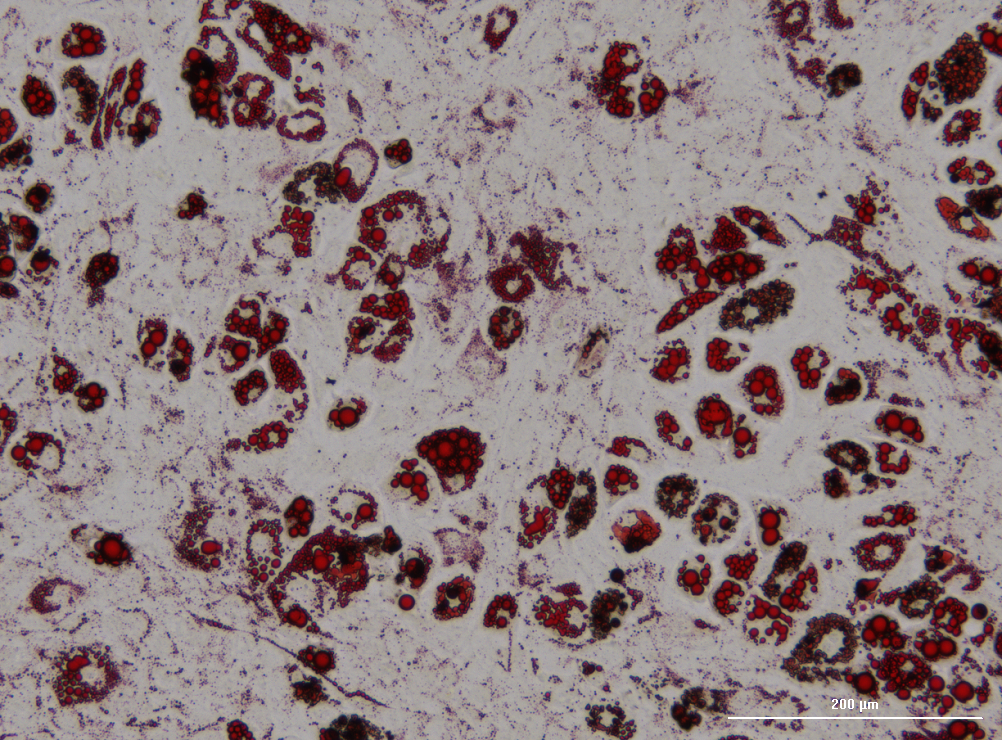

Supplement: Figure 2—figure supplement 1—source data 2. [file elife-74740-fig2-figsupp1-data2.zip › supplementary figure 5 D - source data/suppl fig 5D- source data 3- well2 ctrl 10x.png]

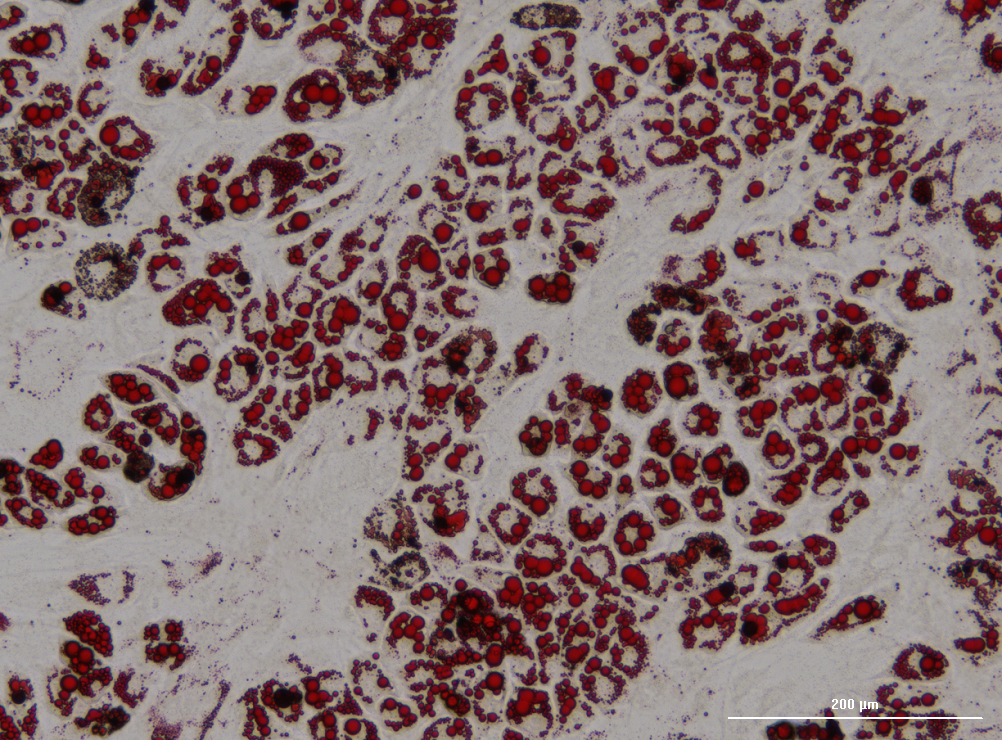

Supplement: Figure 2—figure supplement 1—source data 2. [file elife-74740-fig2-figsupp1-data2.zip › supplementary figure 5 D - source data/suppl fig 5D- source data 5 - well 4 MAF1 10x.png]

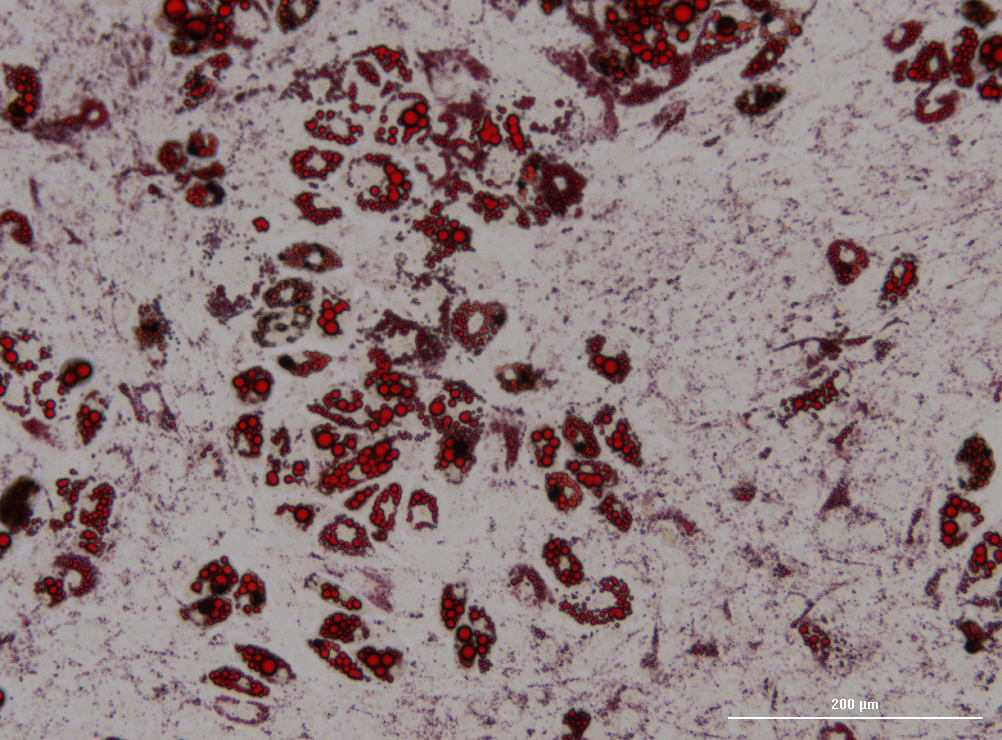

Supplement: Figure 2—figure supplement 1—source data 2. [file elife-74740-fig2-figsupp1-data2.zip › supplementary figure 5 D - source data/suppl fig 5D-source data 2- well1 ctrl 10x.png]

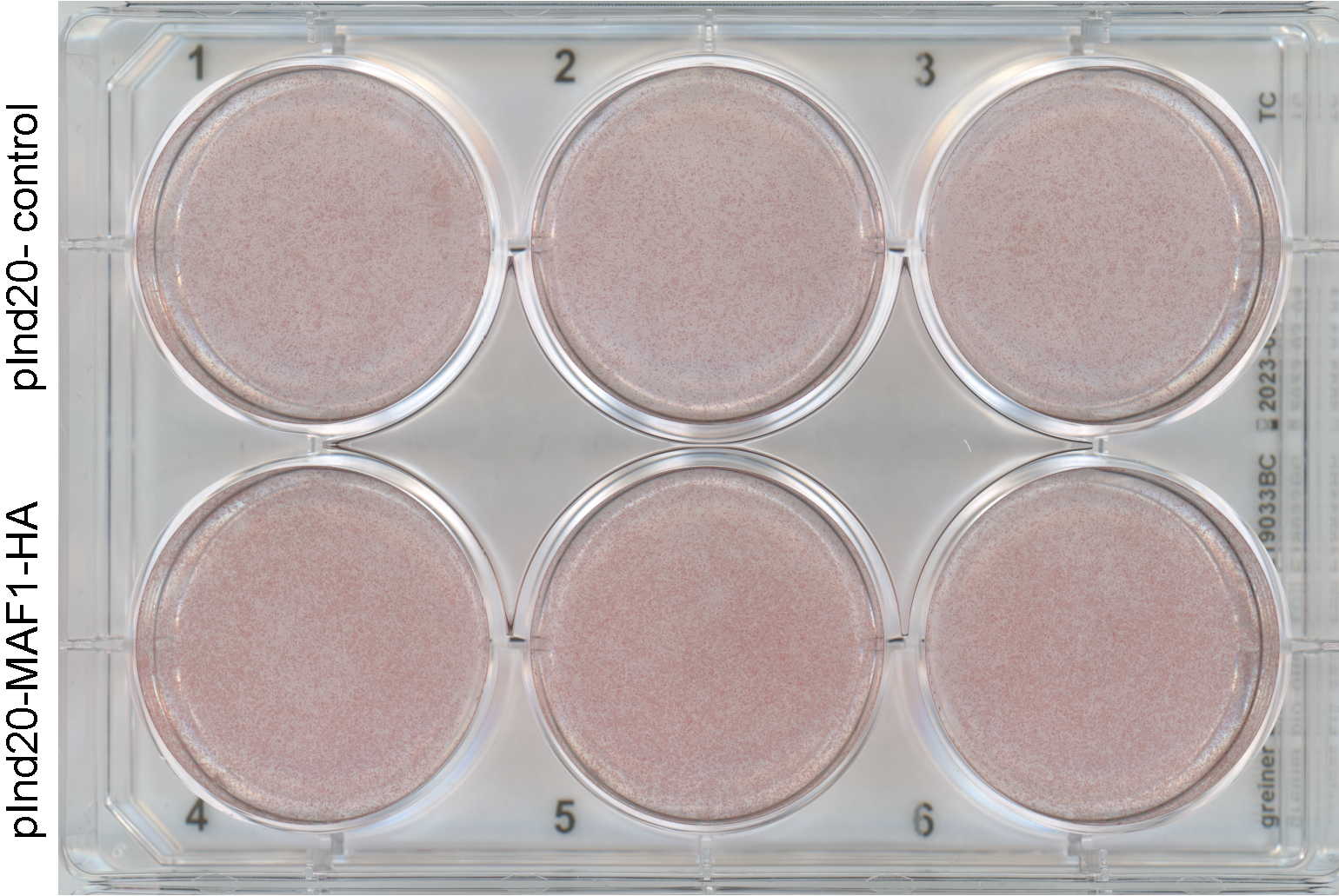

Supplement: Figure 2—figure supplement 1—source data 2. [file elife-74740-fig2-figsupp1-data2.zip › supplementary figure 5 D - source data/supplementary figure 5D - source data 1- ORO.png]

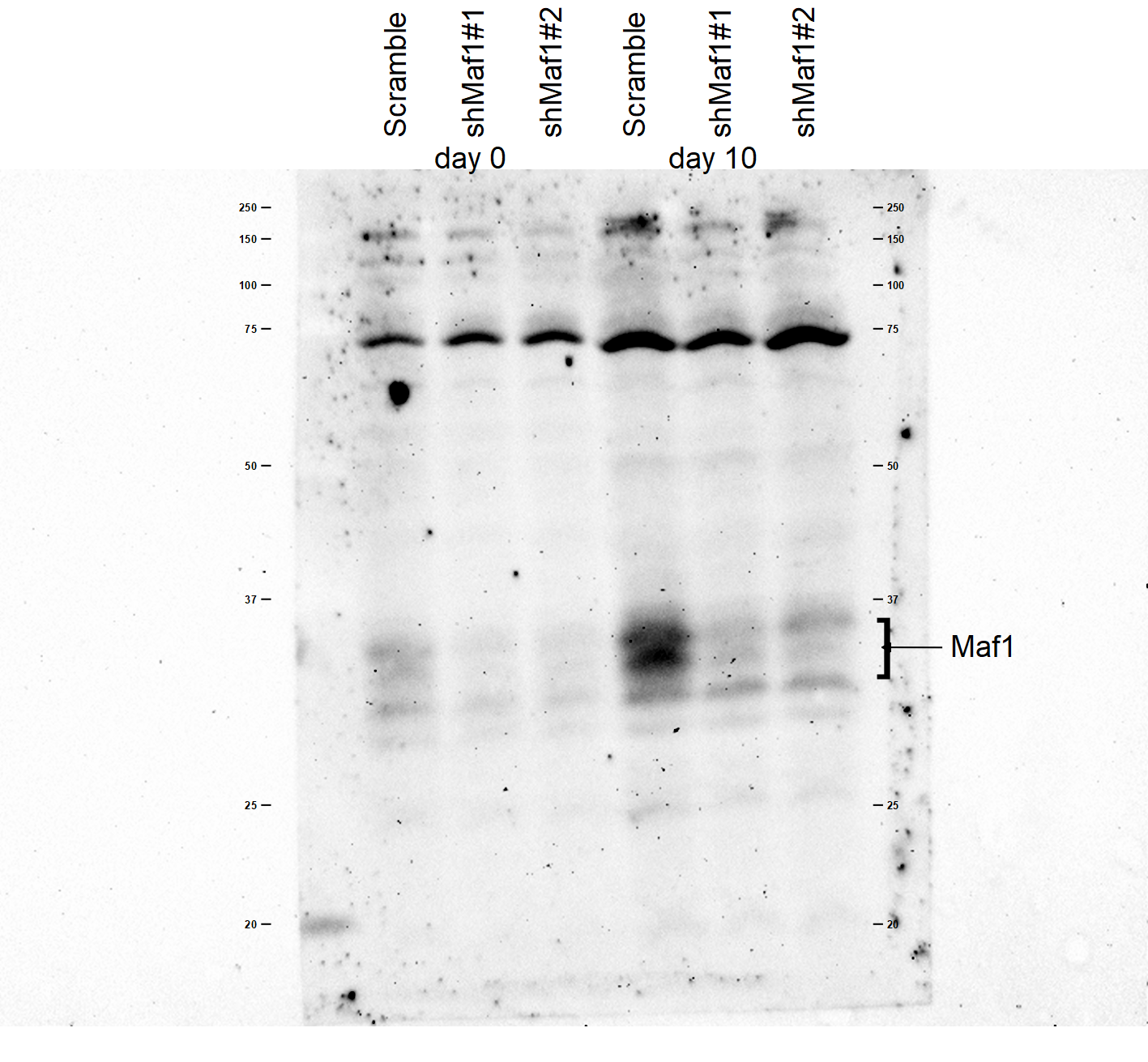

Supplement: Figure 3—source data 1. [file elife-74740-fig3-data1.zip › Figure 3A -source data/figure 3A - source data 1 Maf1 labeled.tif]

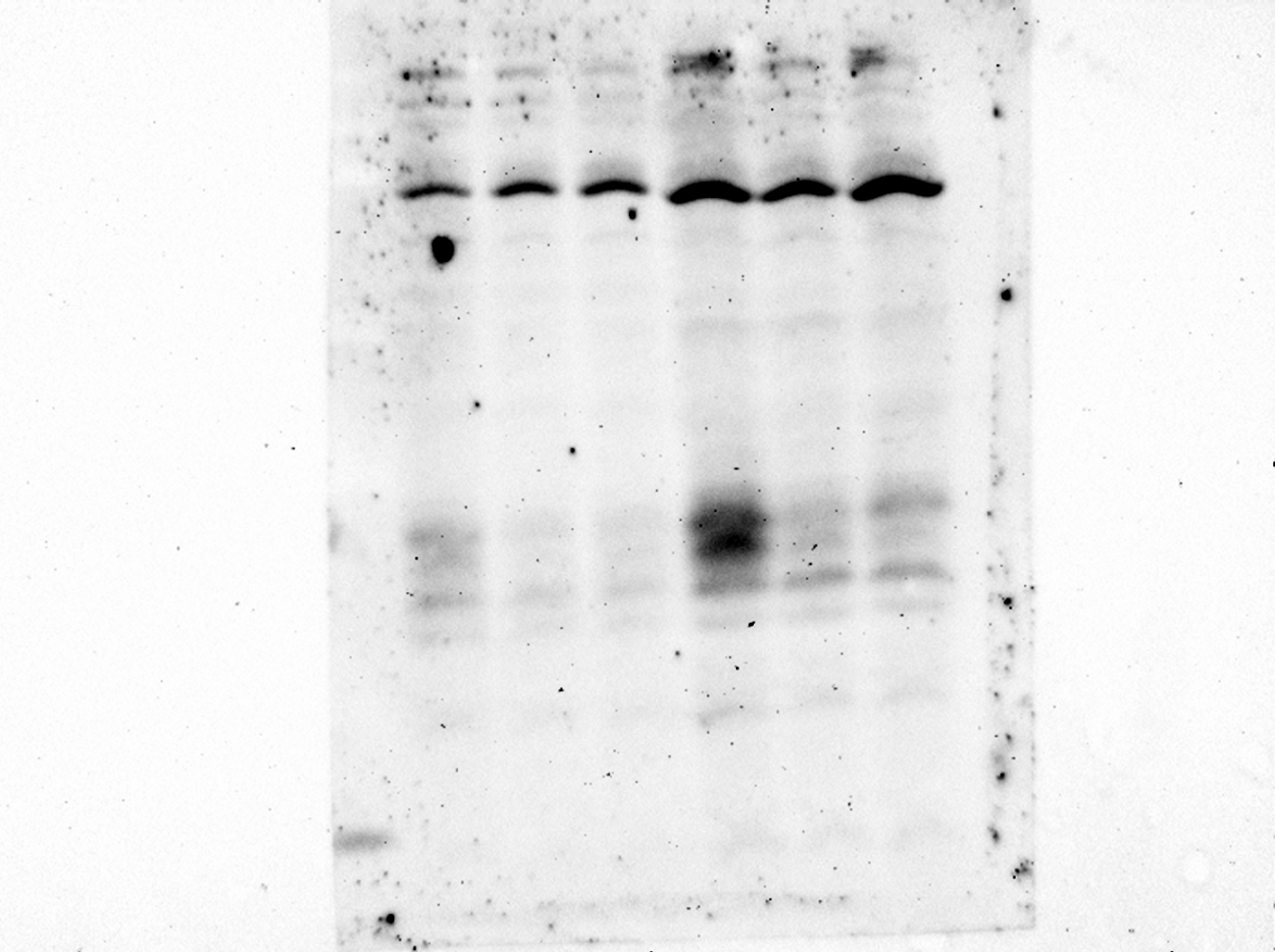

Supplement: Figure 3—source data 1. [file elife-74740-fig3-data1.zip › Figure 3A -source data/figure 3A - source data 2 Maf1.tif]

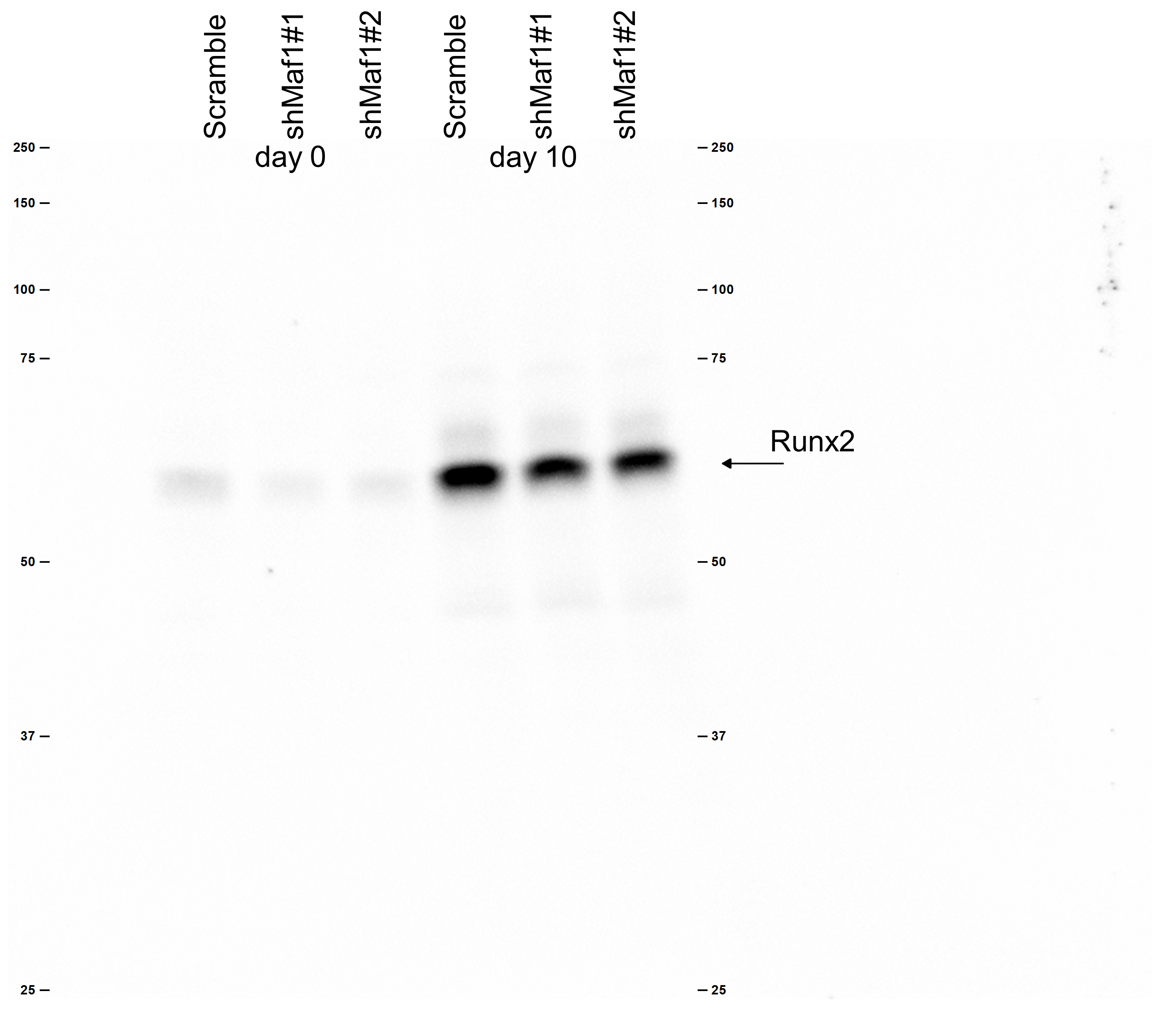

Supplement: Figure 3—source data 1. [file elife-74740-fig3-data1.zip › Figure 3A -source data/figure 3A - source data 3 Runx2 labeled.tif]

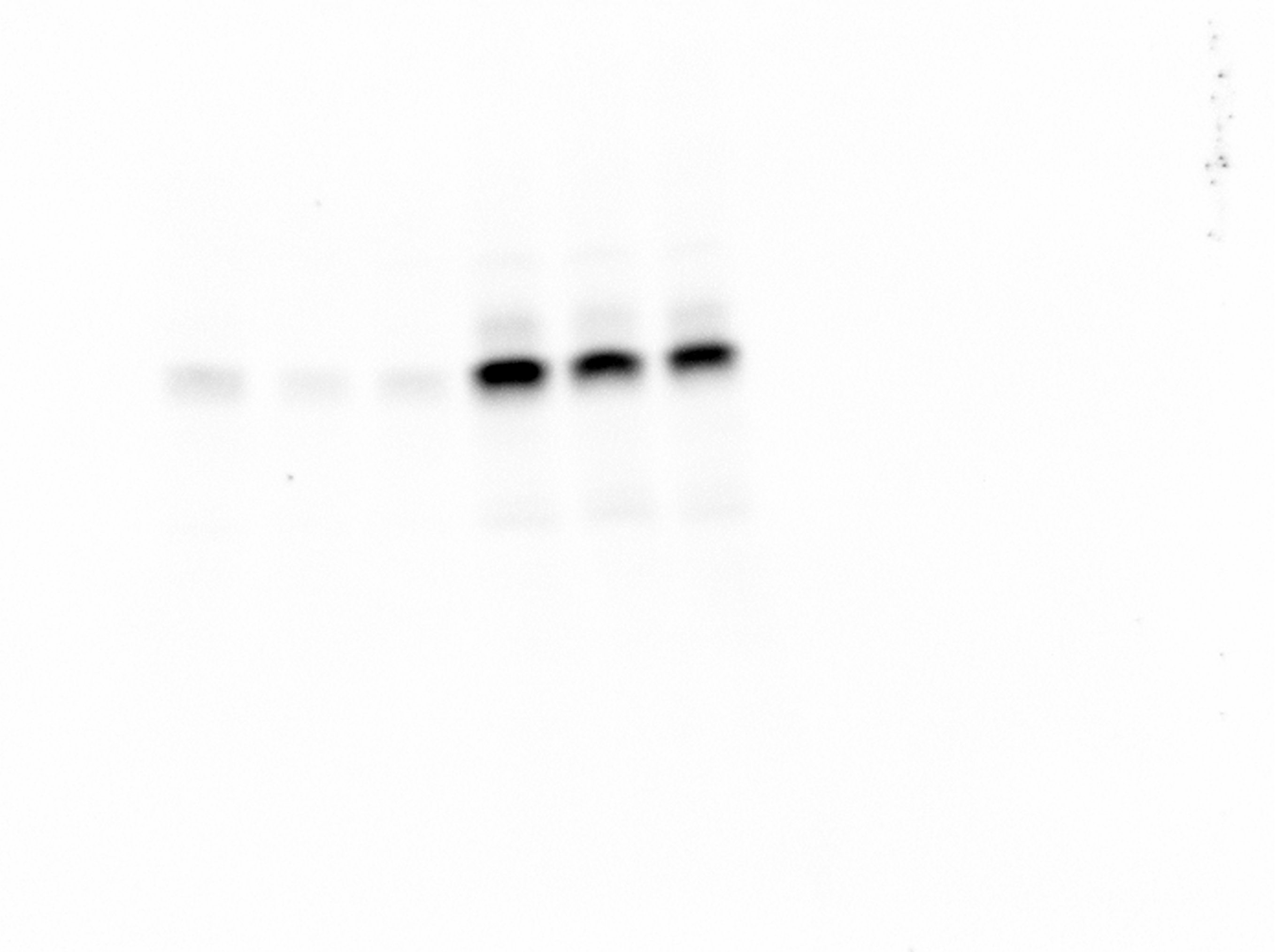

Supplement: Figure 3—source data 1. [file elife-74740-fig3-data1.zip › Figure 3A -source data/figure 3A - source data 4 Runx2.tif]

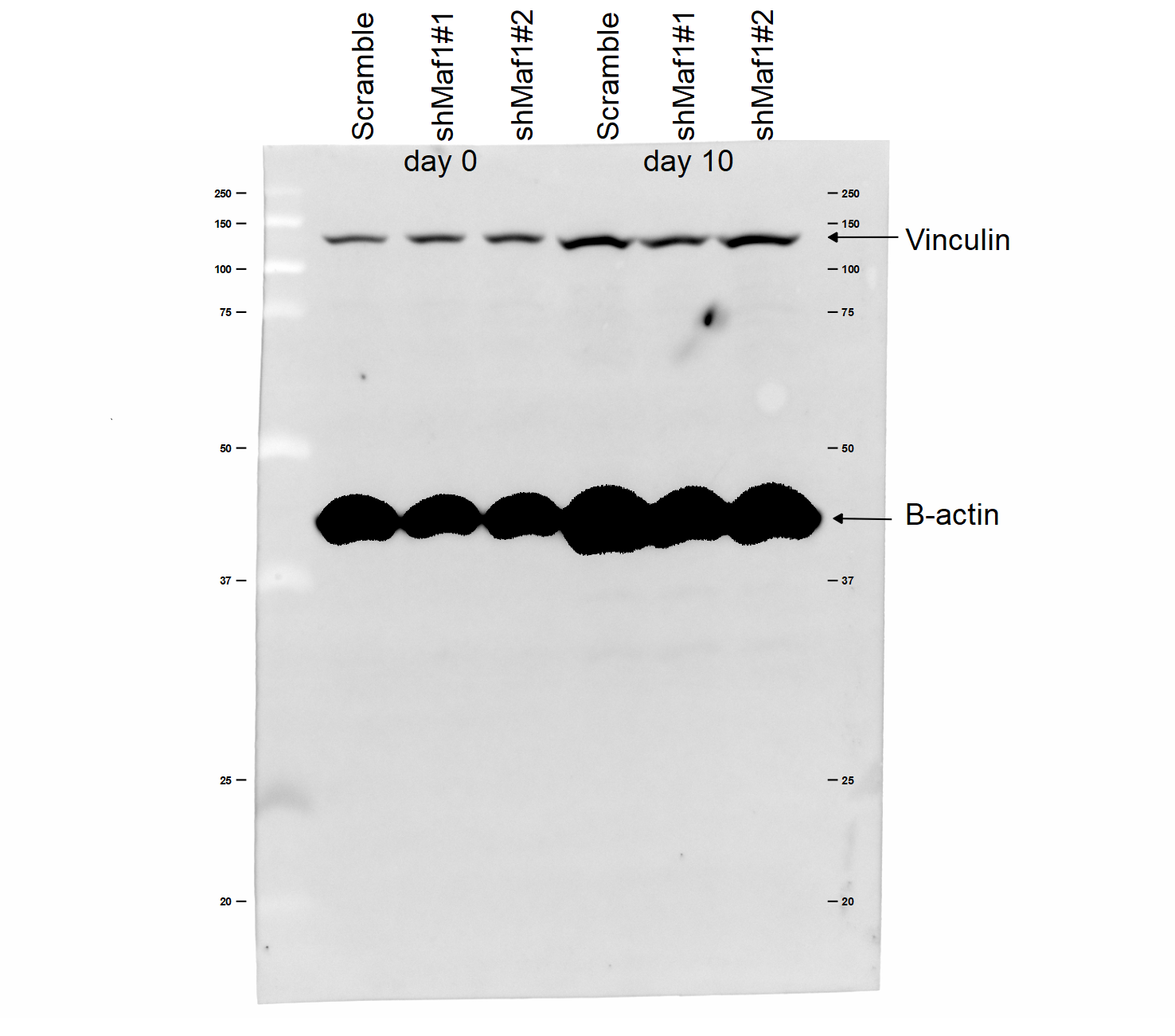

Supplement: Figure 3—source data 1. [file elife-74740-fig3-data1.zip › Figure 3A -source data/figure 3A - source data 5 Vinculin labeled.tif]

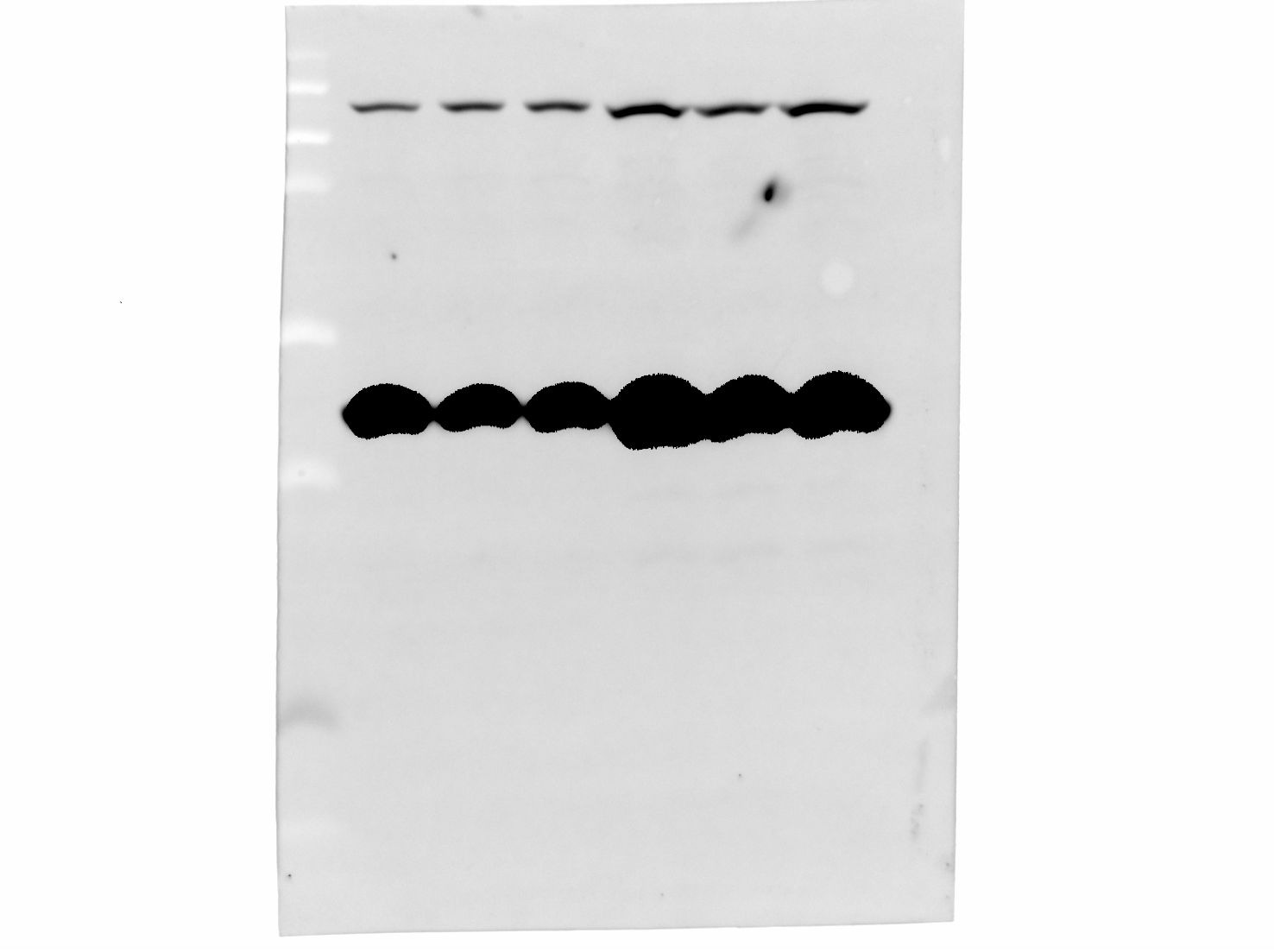

Supplement: Figure 3—source data 1. [file elife-74740-fig3-data1.zip › Figure 3A -source data/figure 3A - source data 6 Vinculin raw.tif]

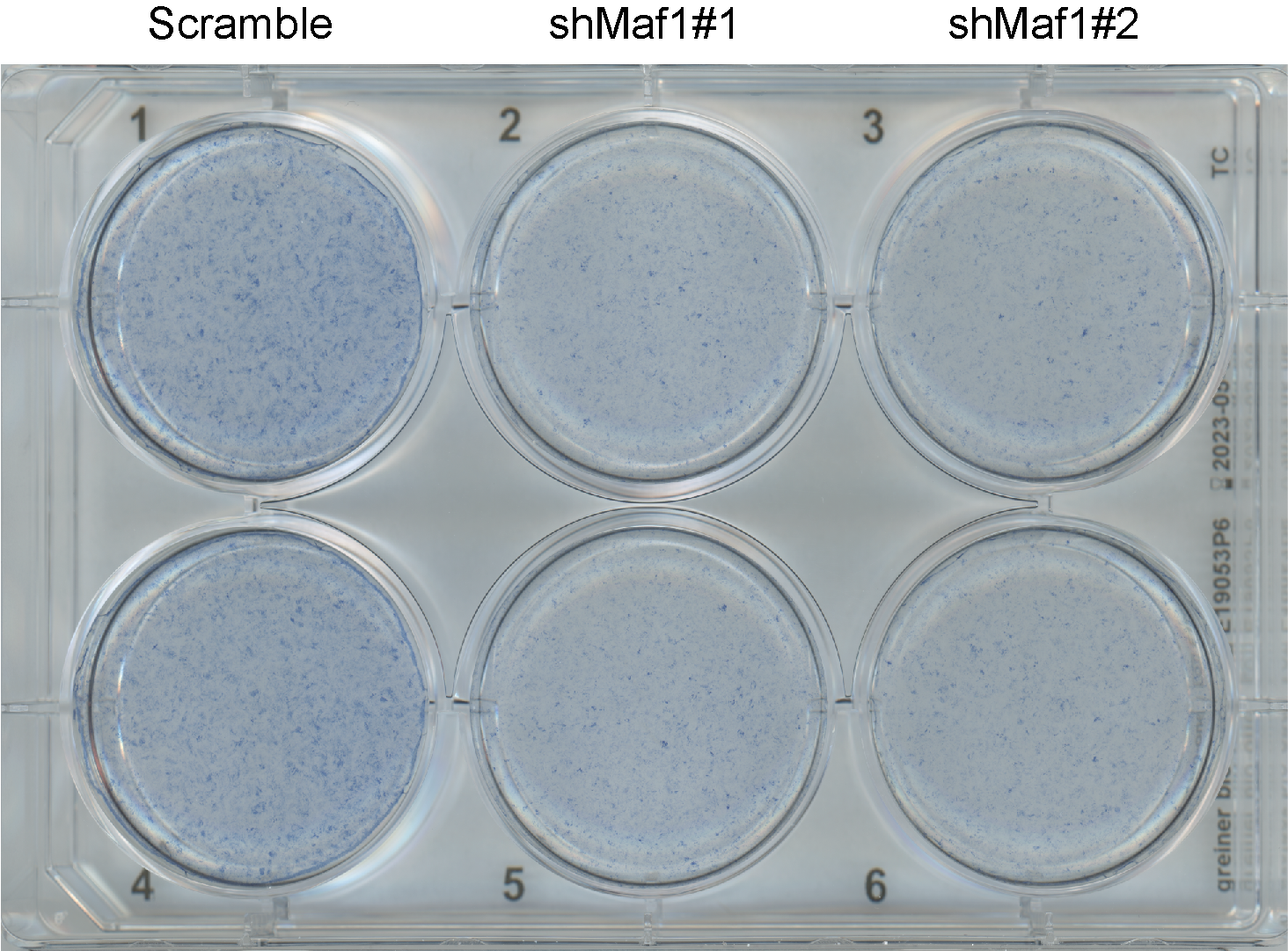

Supplement: Figure 3—source data 2. [file elife-74740-fig3-data2.zip › Figure 3 C-D -source data/Figure 3C source data 1- ALP.png]

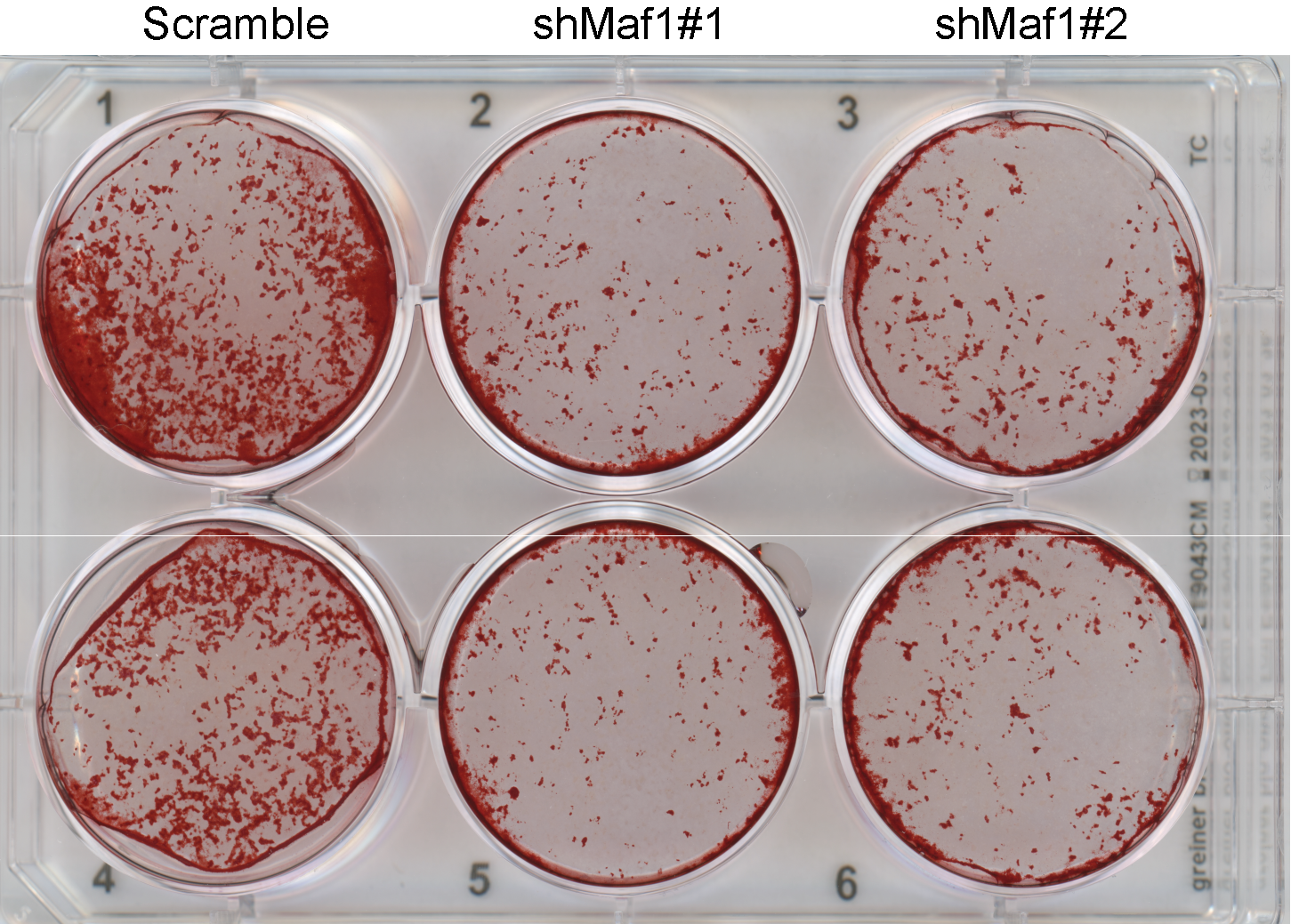

Supplement: Figure 3—source data 2. [file elife-74740-fig3-data2.zip › Figure 3 C-D -source data/Figure 3D source data 2- Alizarin Red.png]

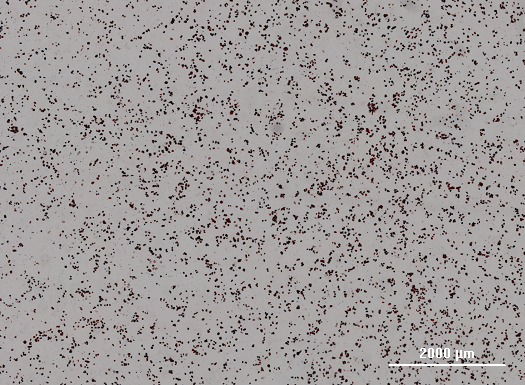

Supplement: Figure 3—figure supplement 1—source data 1. [file elife-74740-fig3-figsupp1-data1.zip › supplementary figure 6 B - source data/suppl fig 6B - source data 10 - well 3 WT stitched.png]

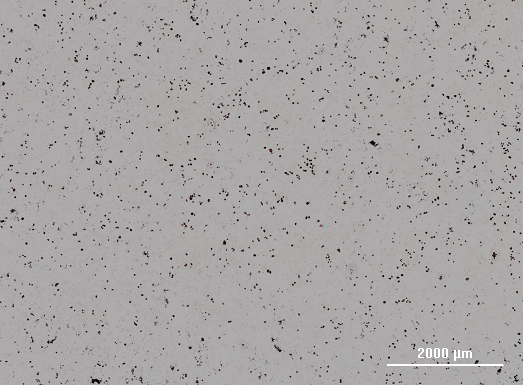

Supplement: Figure 3—figure supplement 1—source data 1. [file elife-74740-fig3-figsupp1-data1.zip › supplementary figure 6 B - source data/suppl fig 6B - source data 11 - well 4 Maf1KO stitched.png]

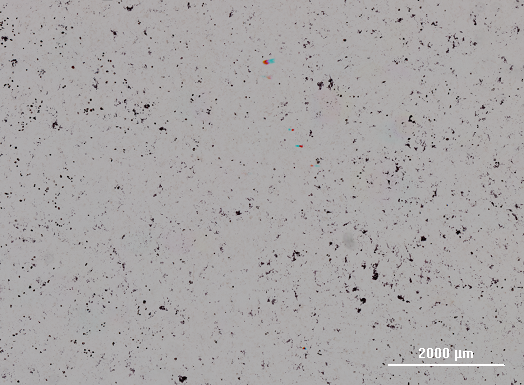

Supplement: Figure 3—figure supplement 1—source data 1. [file elife-74740-fig3-figsupp1-data1.zip › supplementary figure 6 B - source data/suppl fig 6B - source data 13 - well 6 Maf1KO stitched.png]

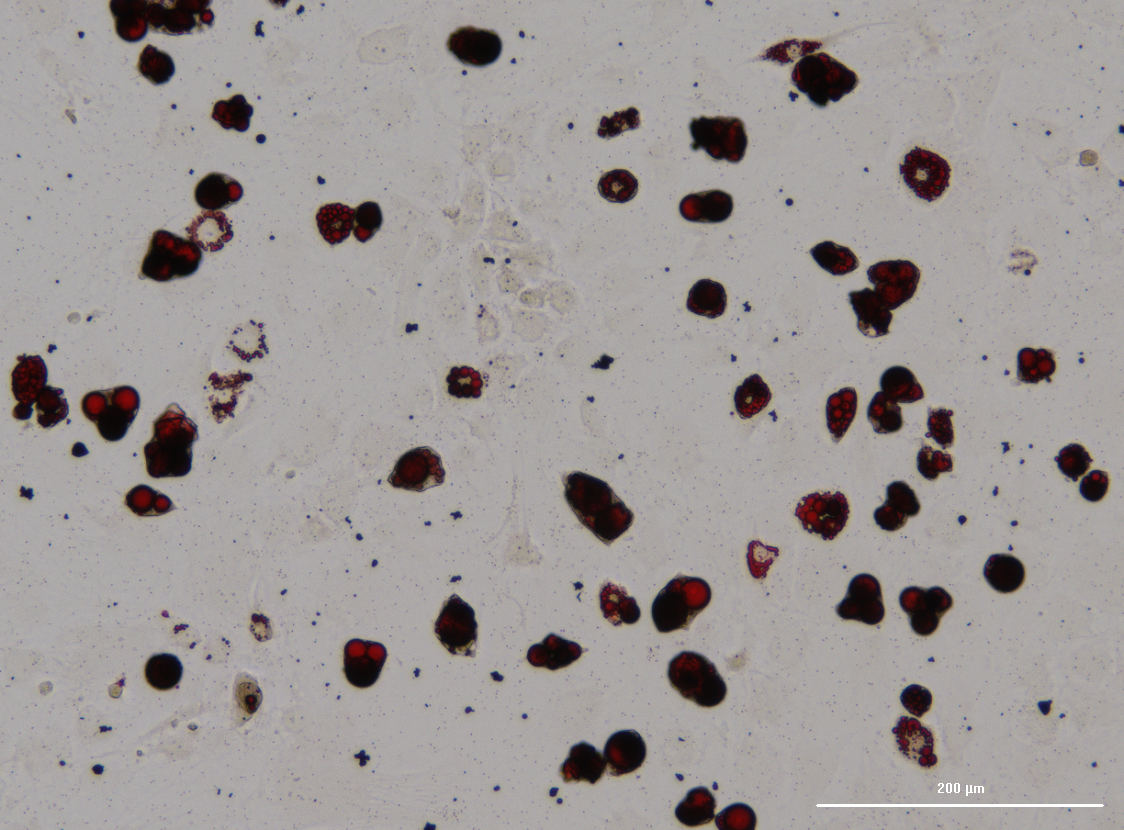

Supplement: Figure 3—figure supplement 1—source data 1. [file elife-74740-fig3-figsupp1-data1.zip › supplementary figure 6 B - source data/suppl fig 6B - source data 2- well 1 WT 10x.png]

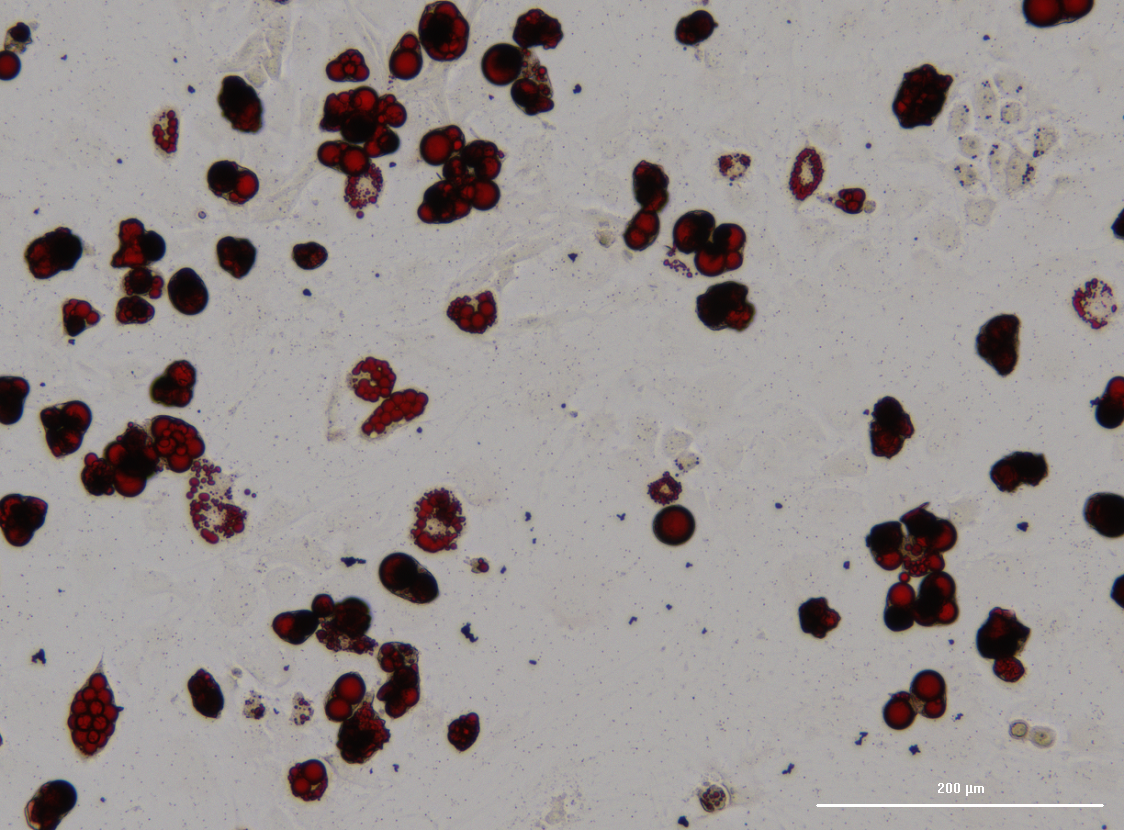

Supplement: Figure 3—figure supplement 1—source data 1. [file elife-74740-fig3-figsupp1-data1.zip › supplementary figure 6 B - source data/suppl fig 6B - source data 3 - well 2 WT 10X.png]

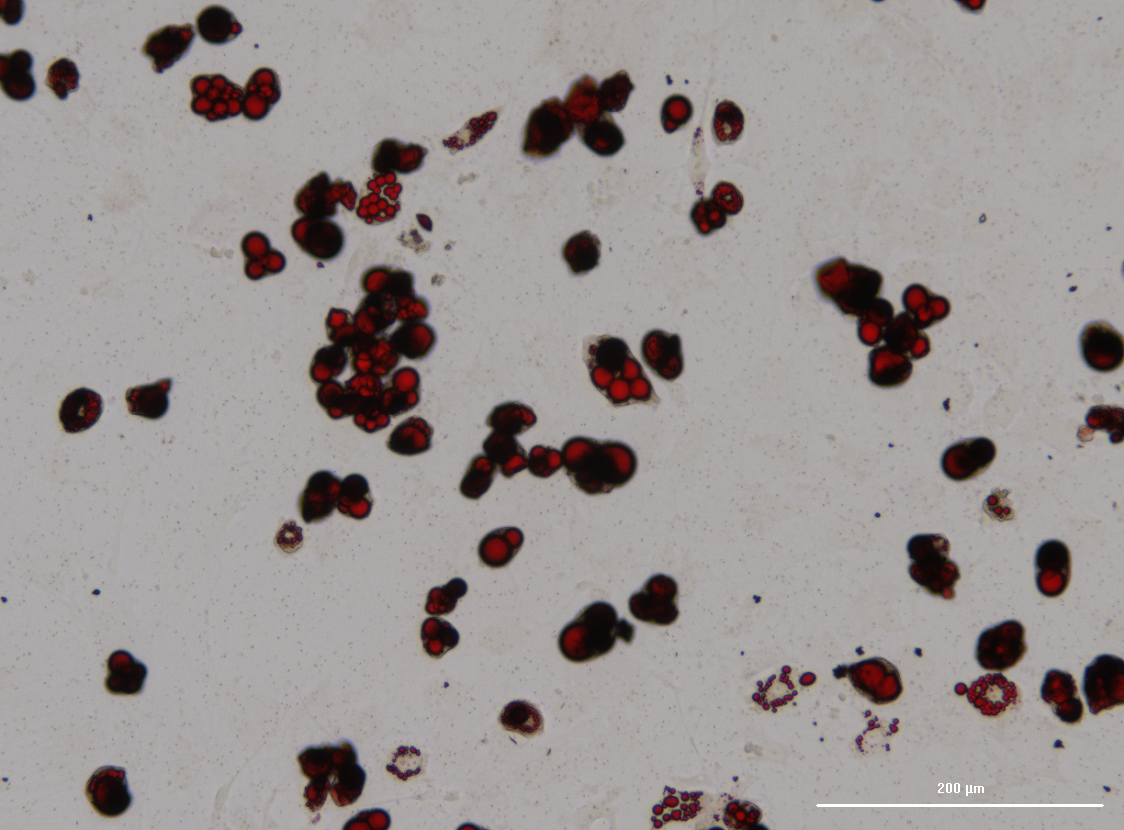

Supplement: Figure 3—figure supplement 1—source data 1. [file elife-74740-fig3-figsupp1-data1.zip › supplementary figure 6 B - source data/suppl fig 6B - source data 4 - well 3 WT 10X.png]

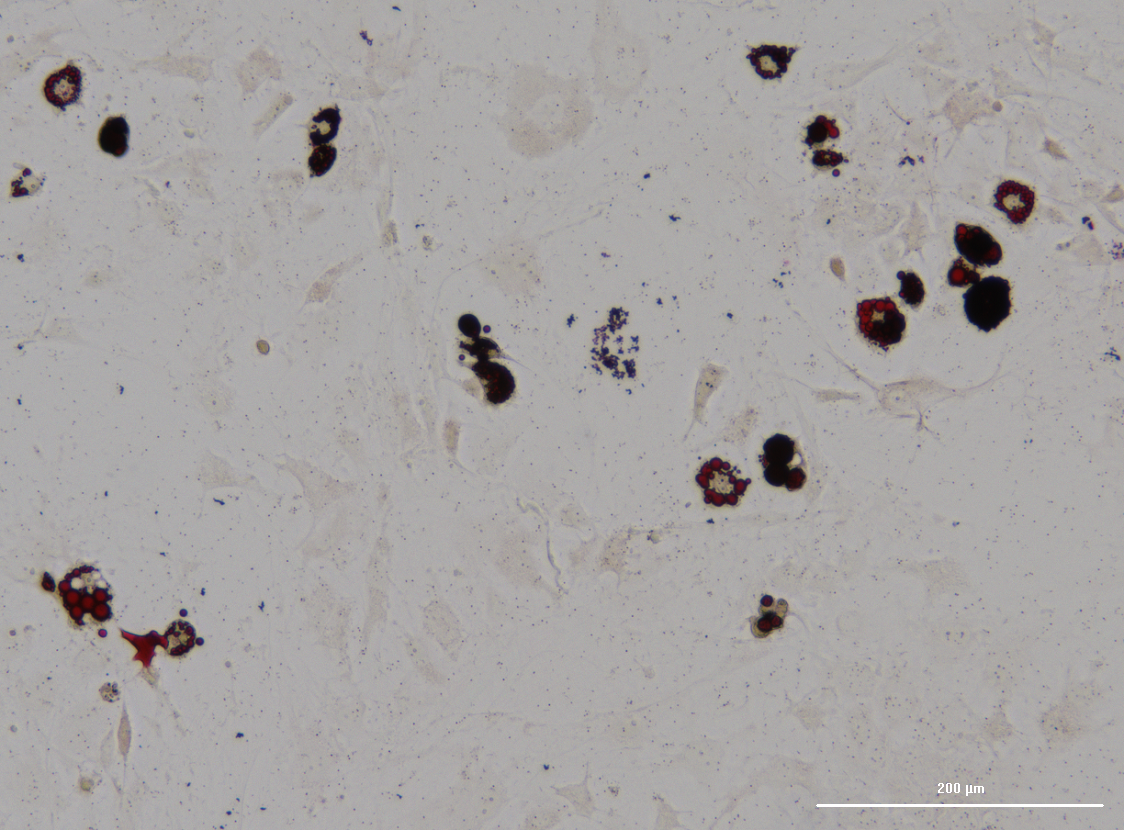

Supplement: Figure 3—figure supplement 1—source data 1. [file elife-74740-fig3-figsupp1-data1.zip › supplementary figure 6 B - source data/suppl fig 6B - source data 5 - well 4 Maf1KO 10x.png]

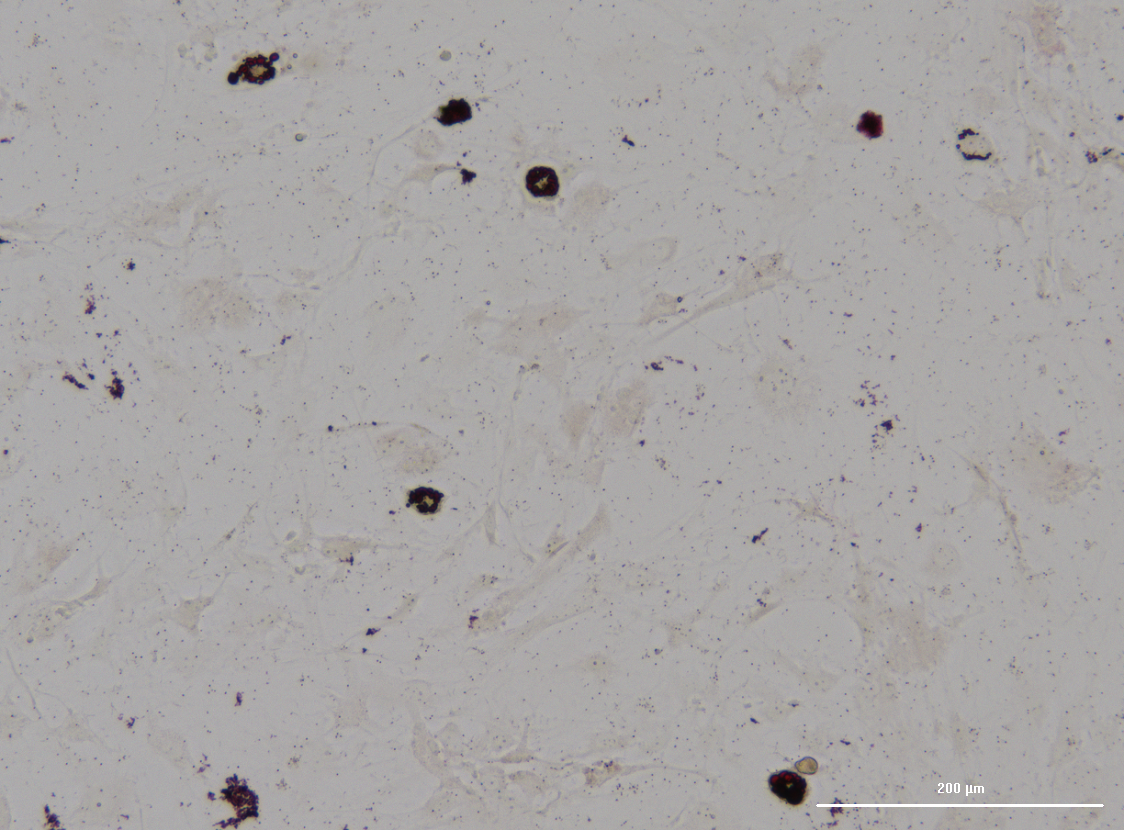

Supplement: Figure 3—figure supplement 1—source data 1. [file elife-74740-fig3-figsupp1-data1.zip › supplementary figure 6 B - source data/suppl fig 6B - source data 7 - well 6 Maf1 KO 10x.png]

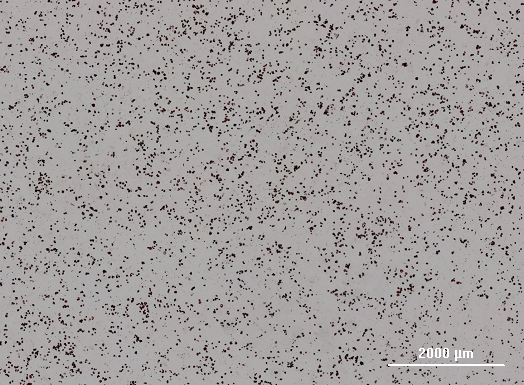

Supplement: Figure 3—figure supplement 1—source data 1. [file elife-74740-fig3-figsupp1-data1.zip › supplementary figure 6 B - source data/suppl fig 6B - source data 8- well 1 WT stitched.png]

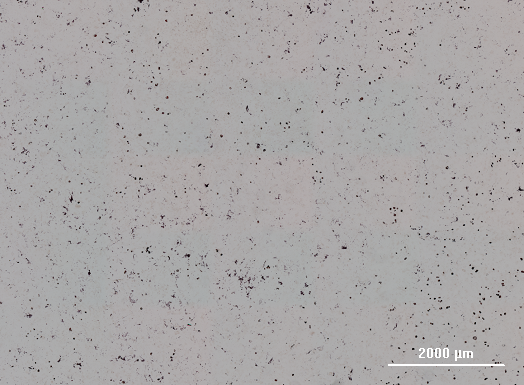

Supplement: Figure 3—figure supplement 1—source data 1. [file elife-74740-fig3-figsupp1-data1.zip › supplementary figure 6 B - source data/suppl fig 6B- source data 12- well 5 Maf1KO stitched.png]

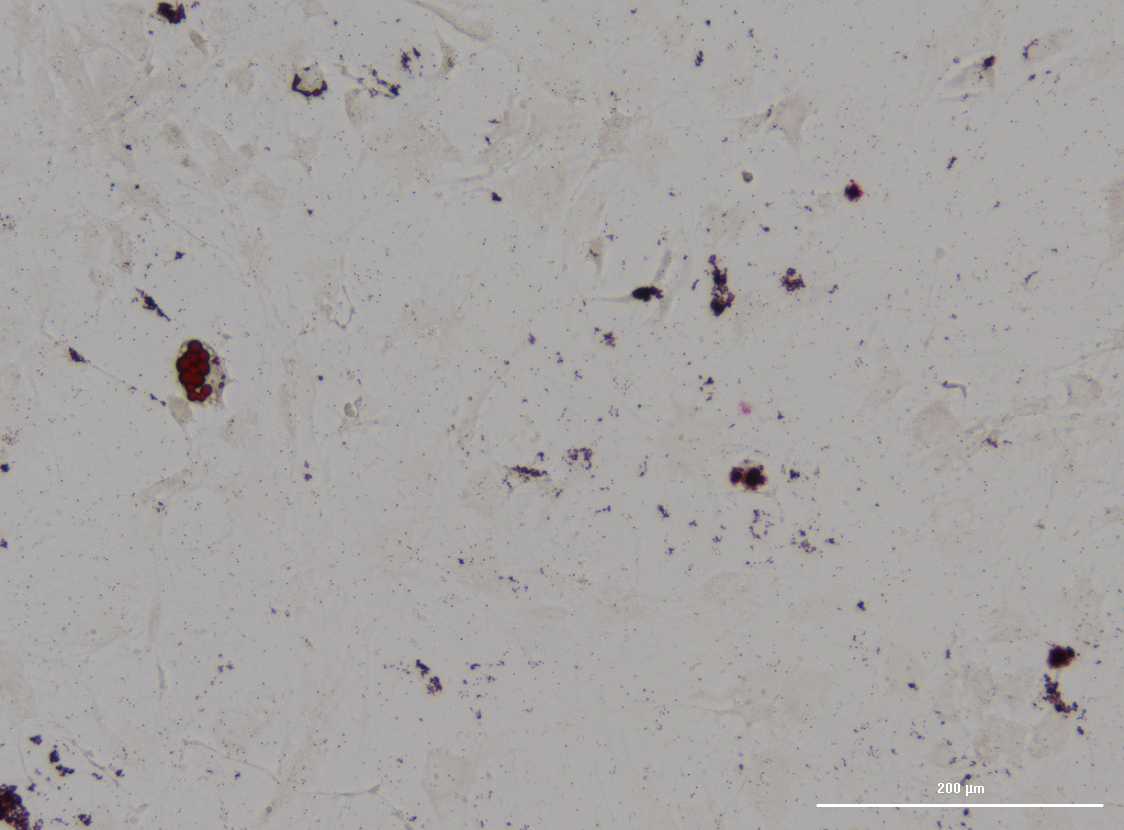

Supplement: Figure 3—figure supplement 1—source data 1. [file elife-74740-fig3-figsupp1-data1.zip › supplementary figure 6 B - source data/suppl fig 6B- source data 6 - well 5 Maf1KO 10x.png]

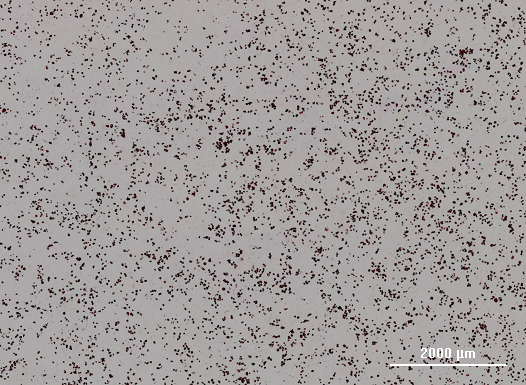

Supplement: Figure 3—figure supplement 1—source data 1. [file elife-74740-fig3-figsupp1-data1.zip › supplementary figure 6 B - source data/supple fig 4B - source data 9- well 2 WT stitched.png]

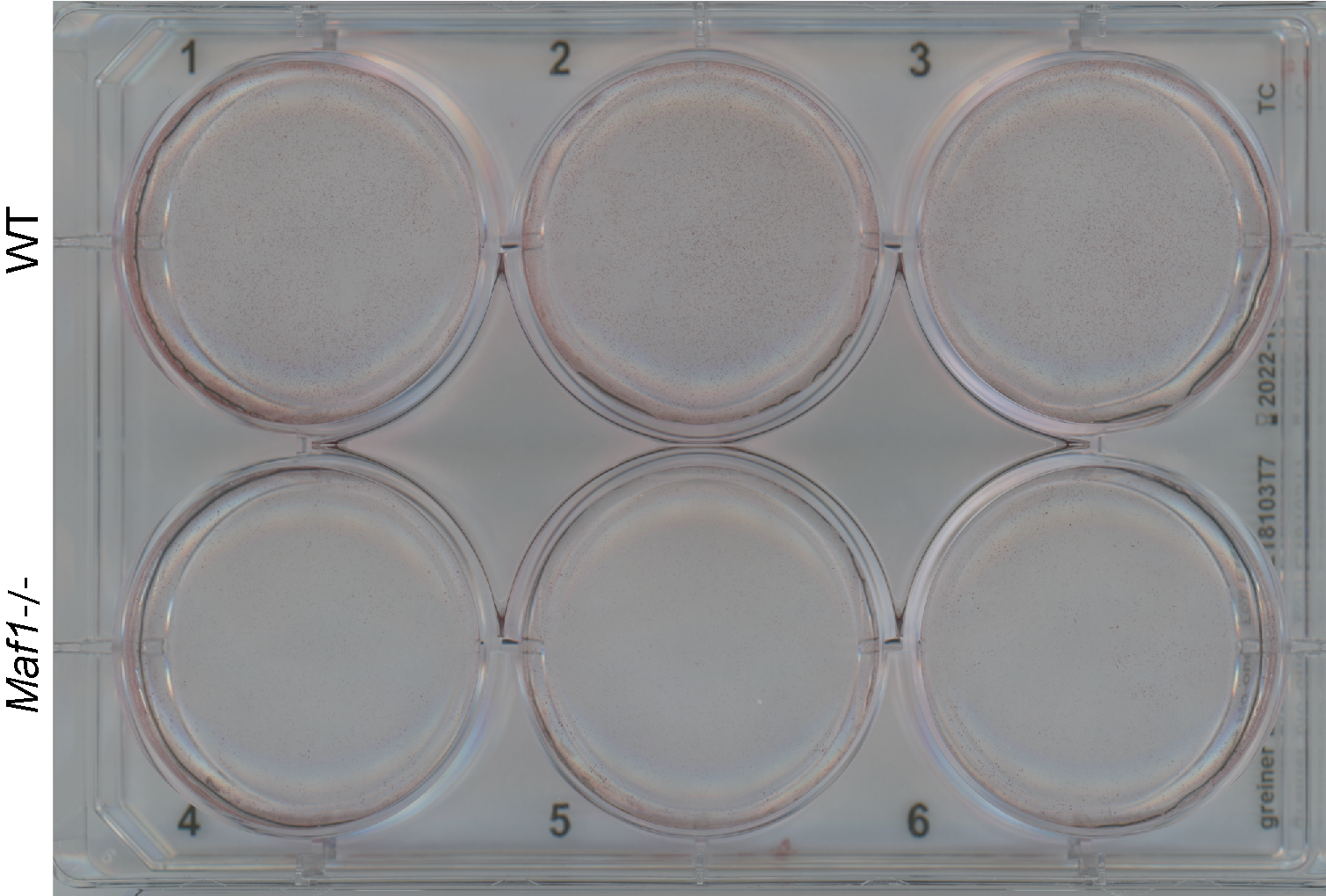

Supplement: Figure 3—figure supplement 1—source data 1. [file elife-74740-fig3-figsupp1-data1.zip › supplementary figure 6 B - source data/supplementary figure 6 B- source datat 1 ORO.png]

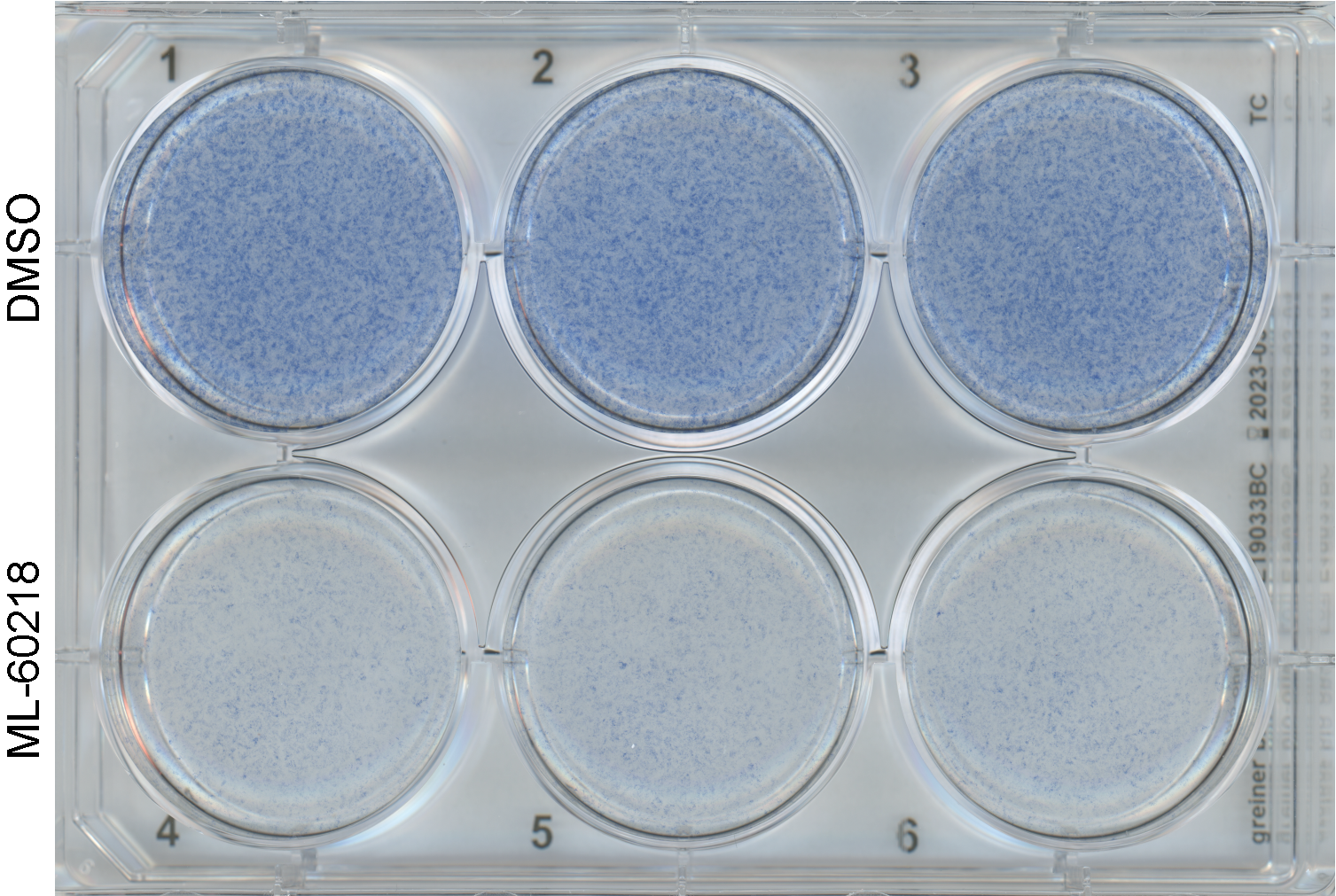

Supplement: Figure 4—source data 1. [file elife-74740-fig4-data1.zip › figure 4 B-C -source data/Figure 4B - source data 1- ALP.png]

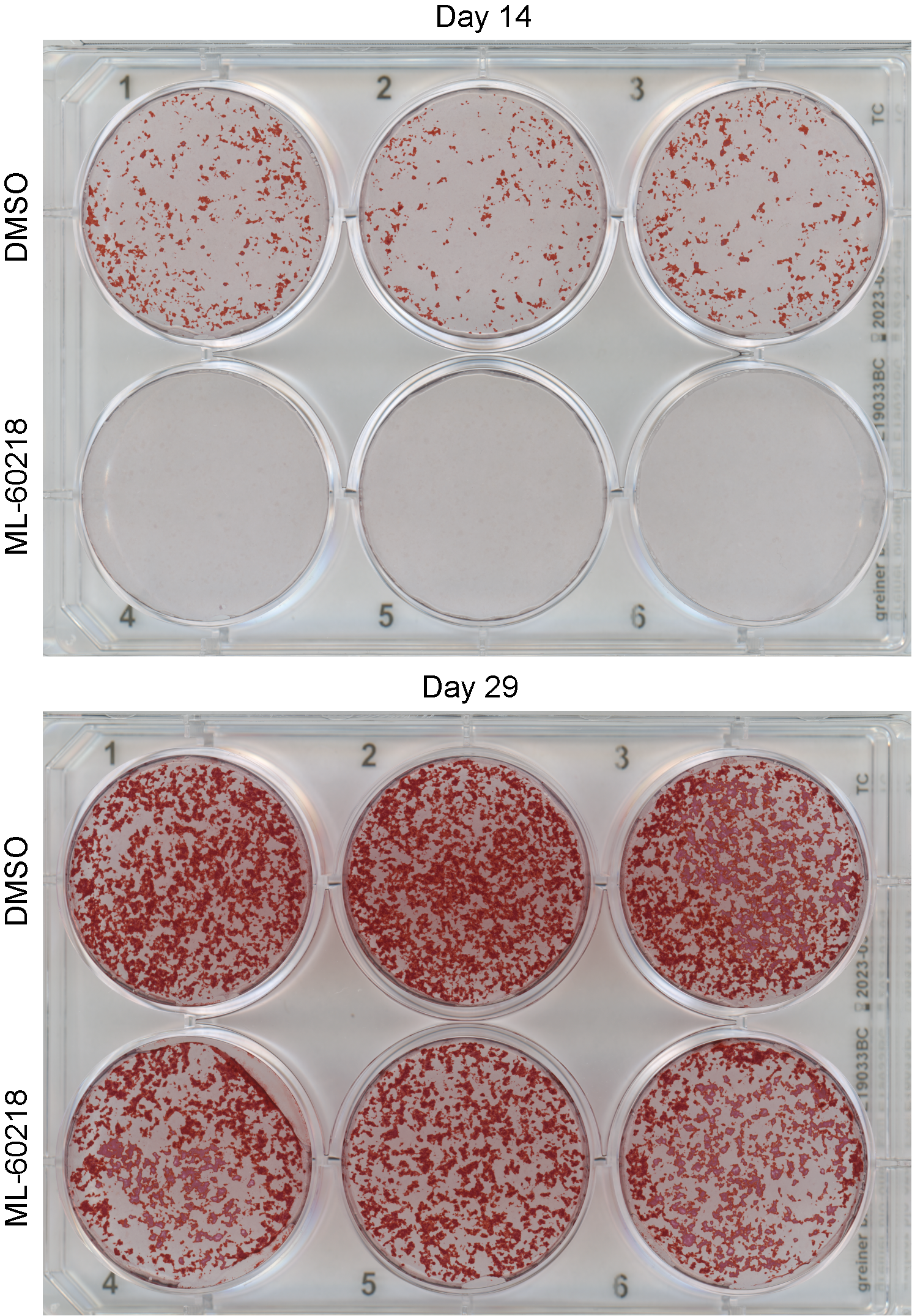

Supplement: Figure 4—source data 1. [file elife-74740-fig4-data1.zip › figure 4 B-C -source data/figure 4C - source data 2-Alizarin red.png]

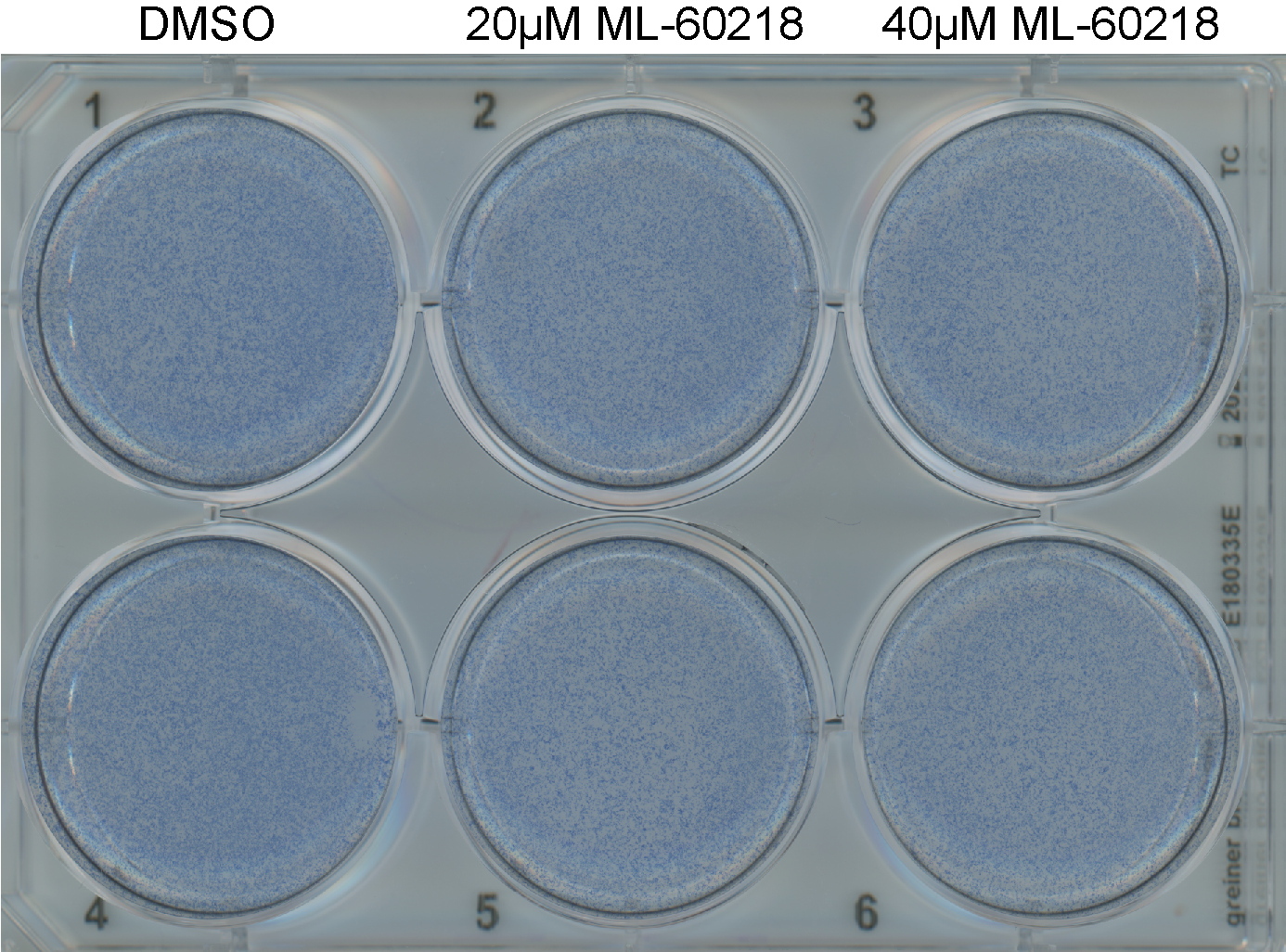

Supplement: Figure 4—figure supplement 1—source data 1. [file elife-74740-fig4-figsupp1-data1.zip › supplementary figure 7 AB - source data/supplementary figure 7A - source data 1 - Alp.png]

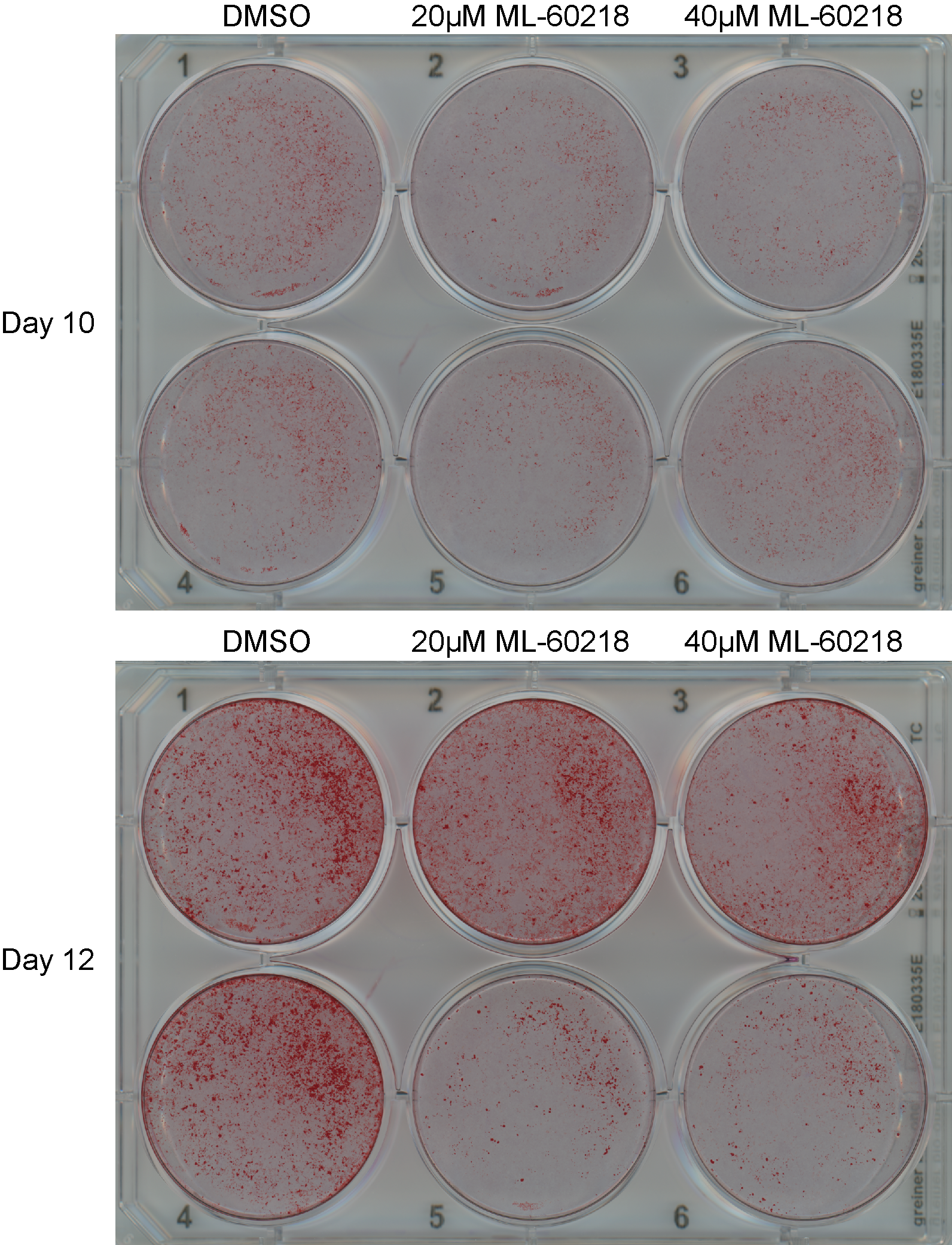

Supplement: Figure 4—figure supplement 1—source data 1. [file elife-74740-fig4-figsupp1-data1.zip › supplementary figure 7 AB - source data/supplementary figure 7B - source data 2 - Alizarin red.png]

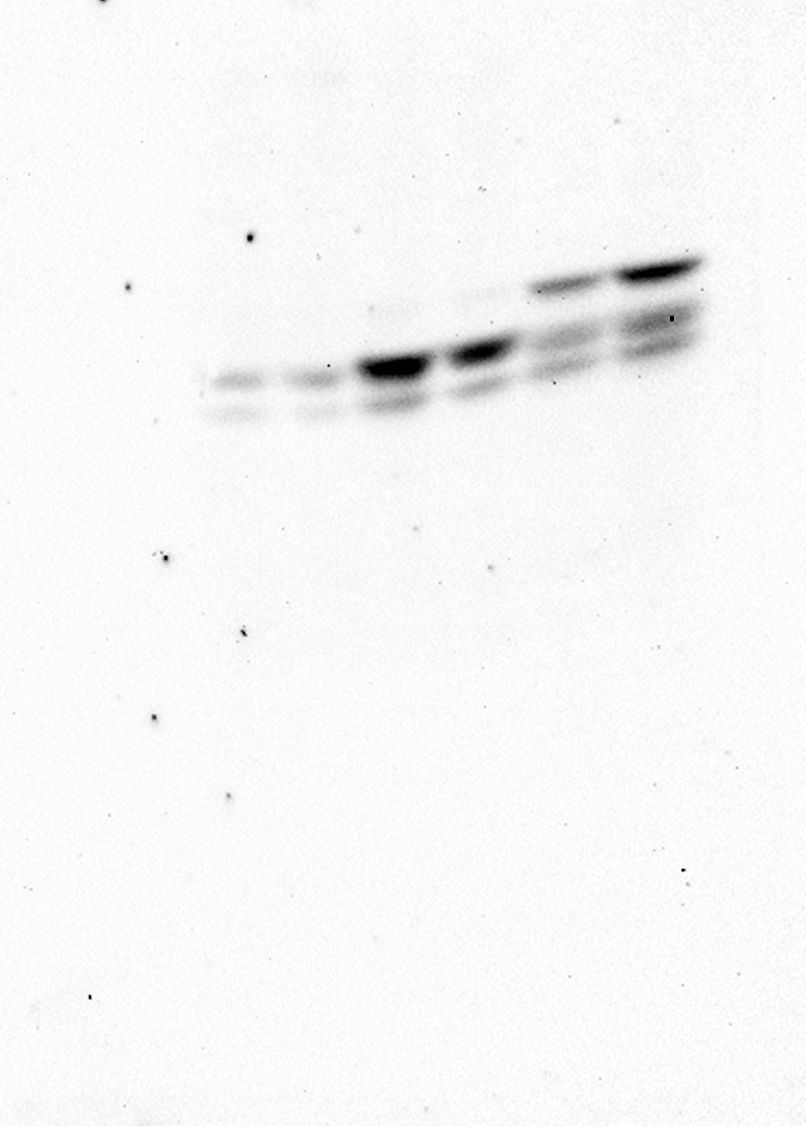

Supplement: Figure 4—figure supplement 2—source data 1. [file elife-74740-fig4-figsupp2-data1.zip › supplementary figure 8 B - source data/supplementary figure 8B - source data 2 Ppary.tif]

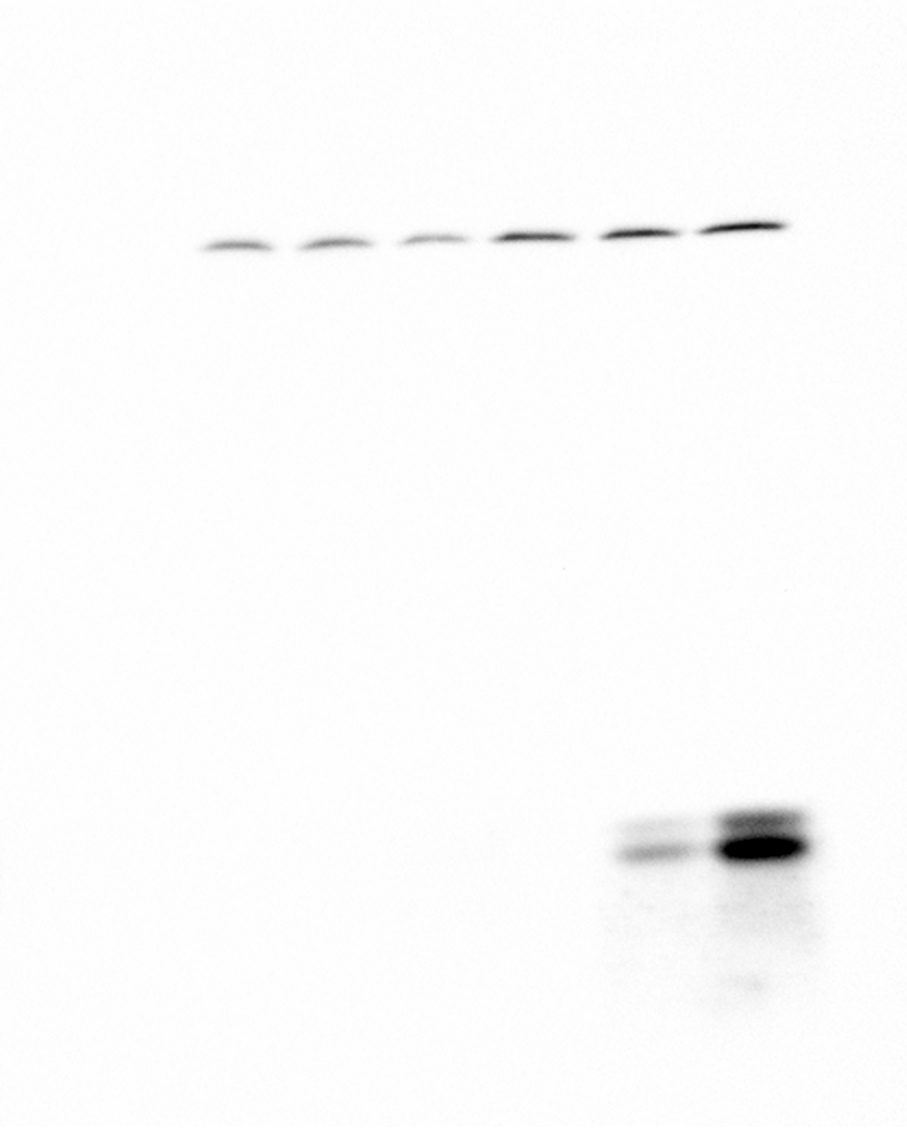

Supplement: Figure 4—figure supplement 2—source data 1. [file elife-74740-fig4-figsupp2-data1.zip › supplementary figure 8 B - source data/supplementary figure 8B - source data 4 - Fabp4.tif]

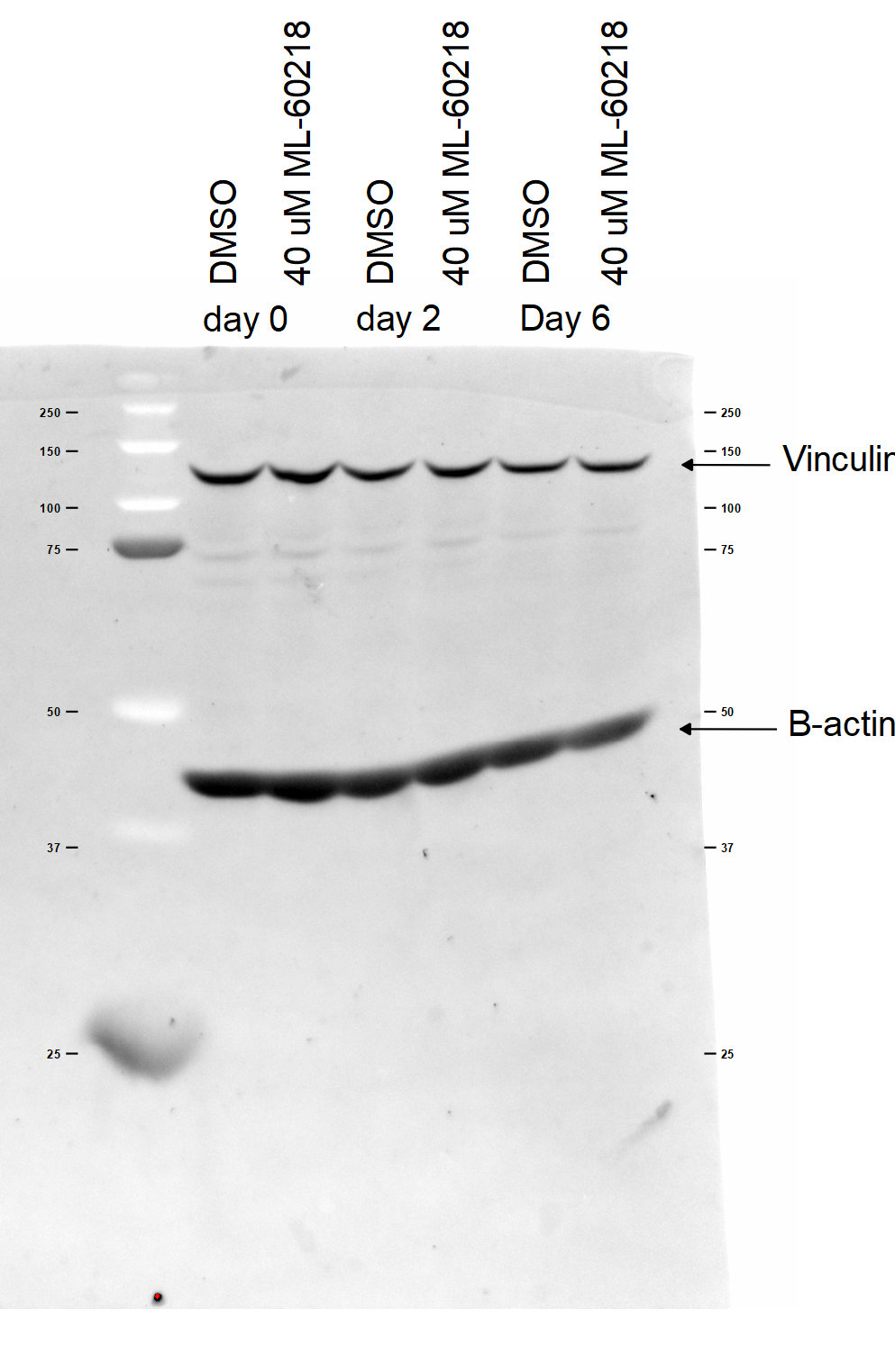

Supplement: Figure 4—figure supplement 2—source data 1. [file elife-74740-fig4-figsupp2-data1.zip › supplementary figure 8 B - source data/supplementary figure 8B - source data 5 - Vinculin labeled.tif]

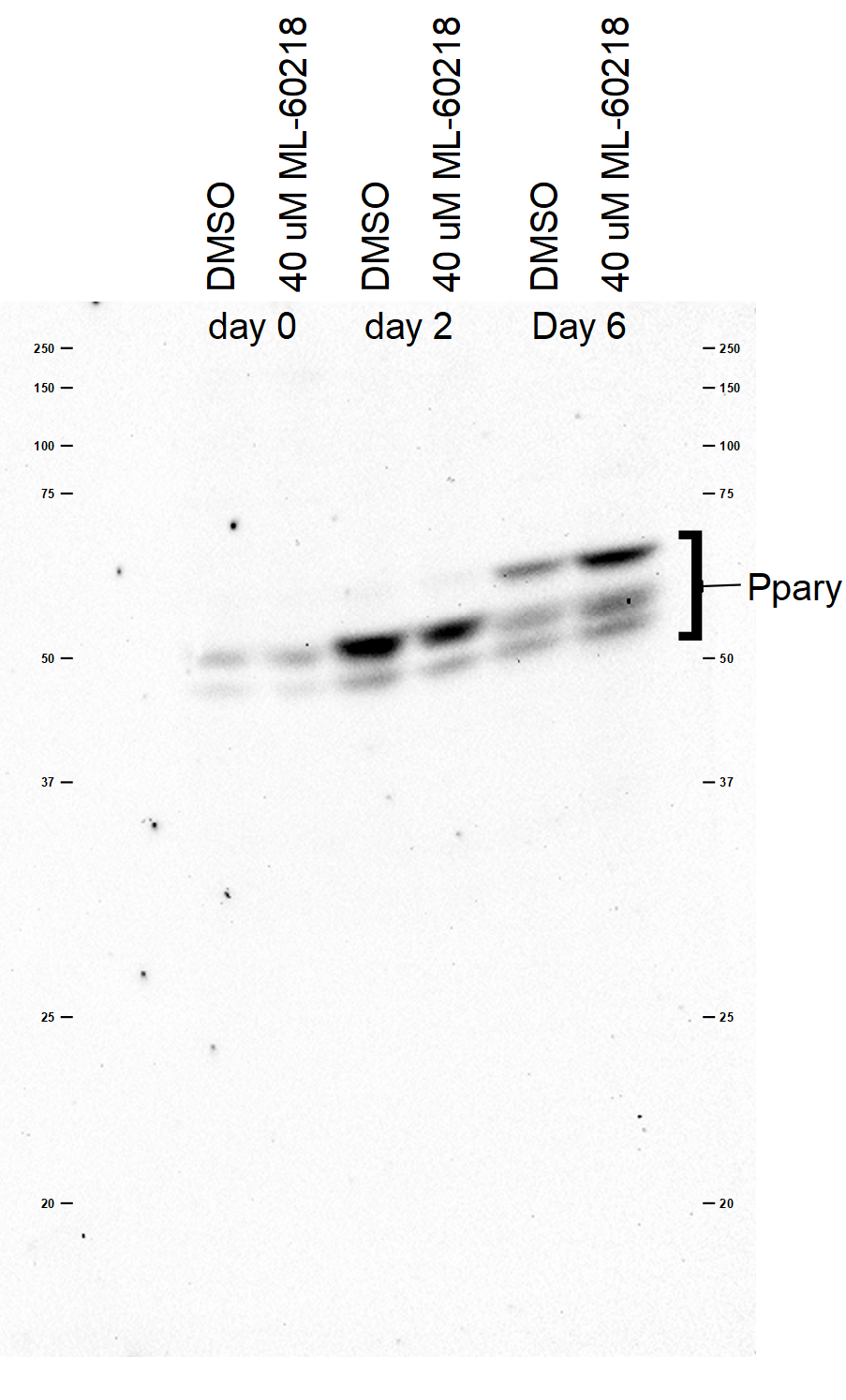

Supplement: Figure 4—figure supplement 2—source data 1. [file elife-74740-fig4-figsupp2-data1.zip › supplementary figure 8 B - source data/supplementary figure 8B source data 1 - Ppary labeled.tif]

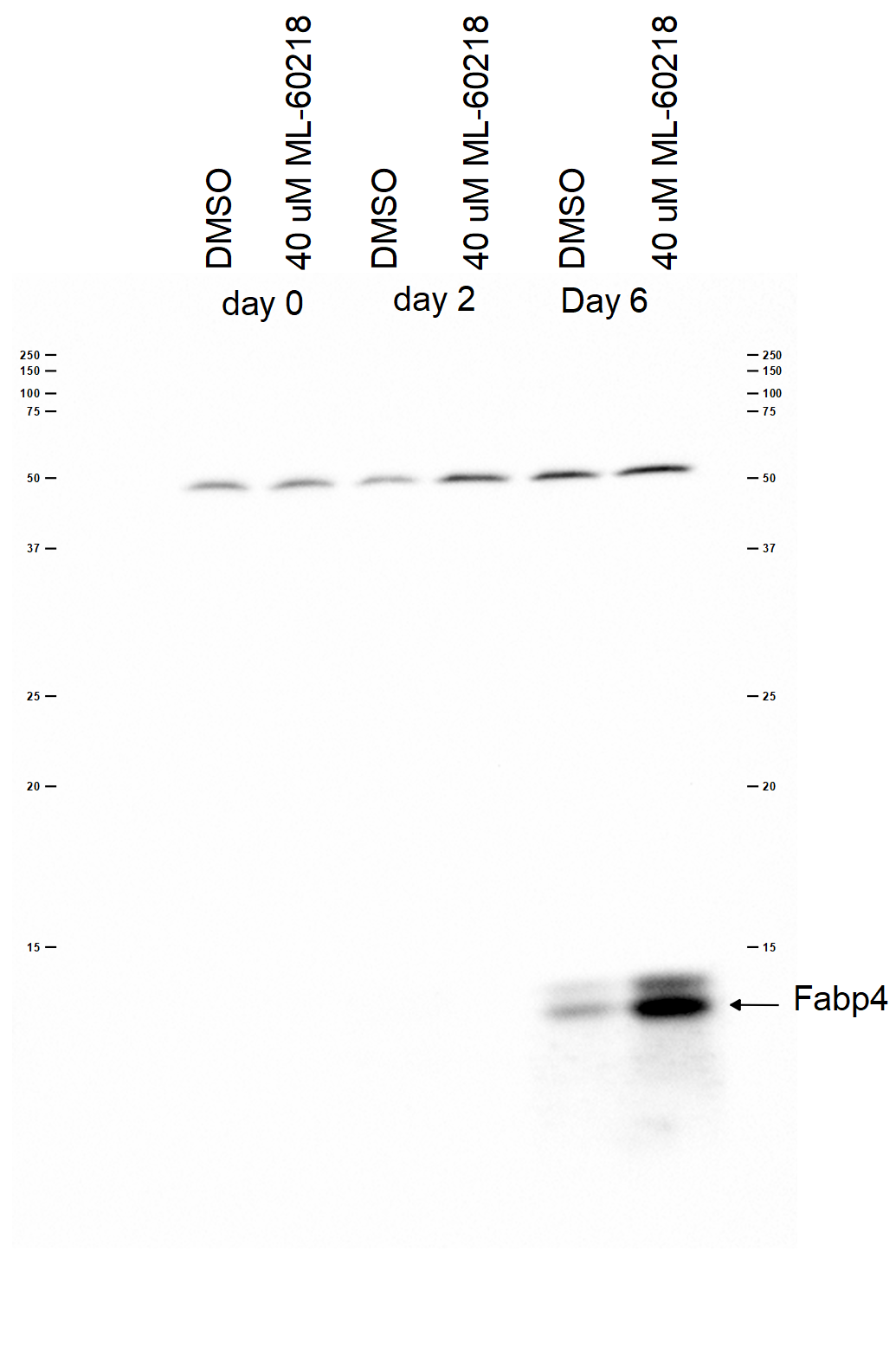

Supplement: Figure 4—figure supplement 2—source data 1. [file elife-74740-fig4-figsupp2-data1.zip › supplementary figure 8 B - source data/supplementary figure 8B source data 3 - Fabp4 labeled.tif]

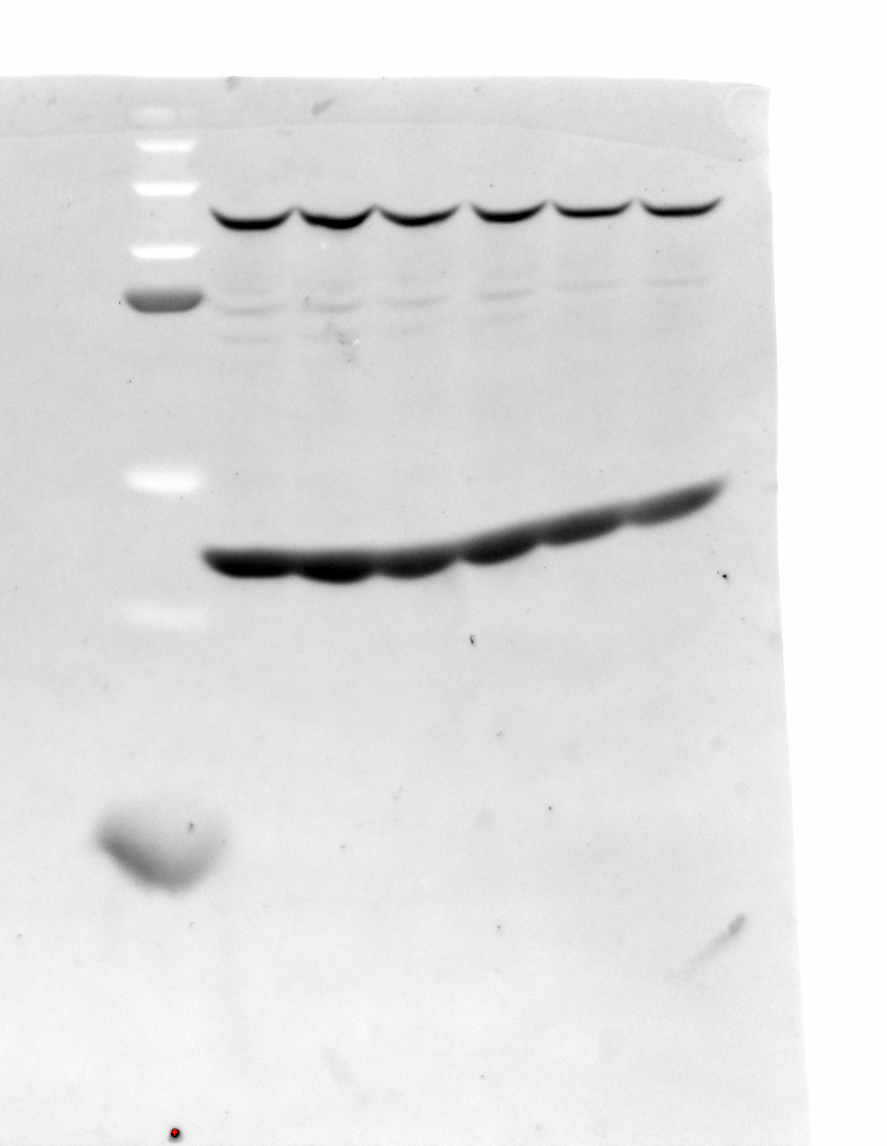

Supplement: Figure 4—figure supplement 2—source data 1. [file elife-74740-fig4-figsupp2-data1.zip › supplementary figure 8 B - source data/supplementary figure 8B source data 6 - Vinculin.tif]

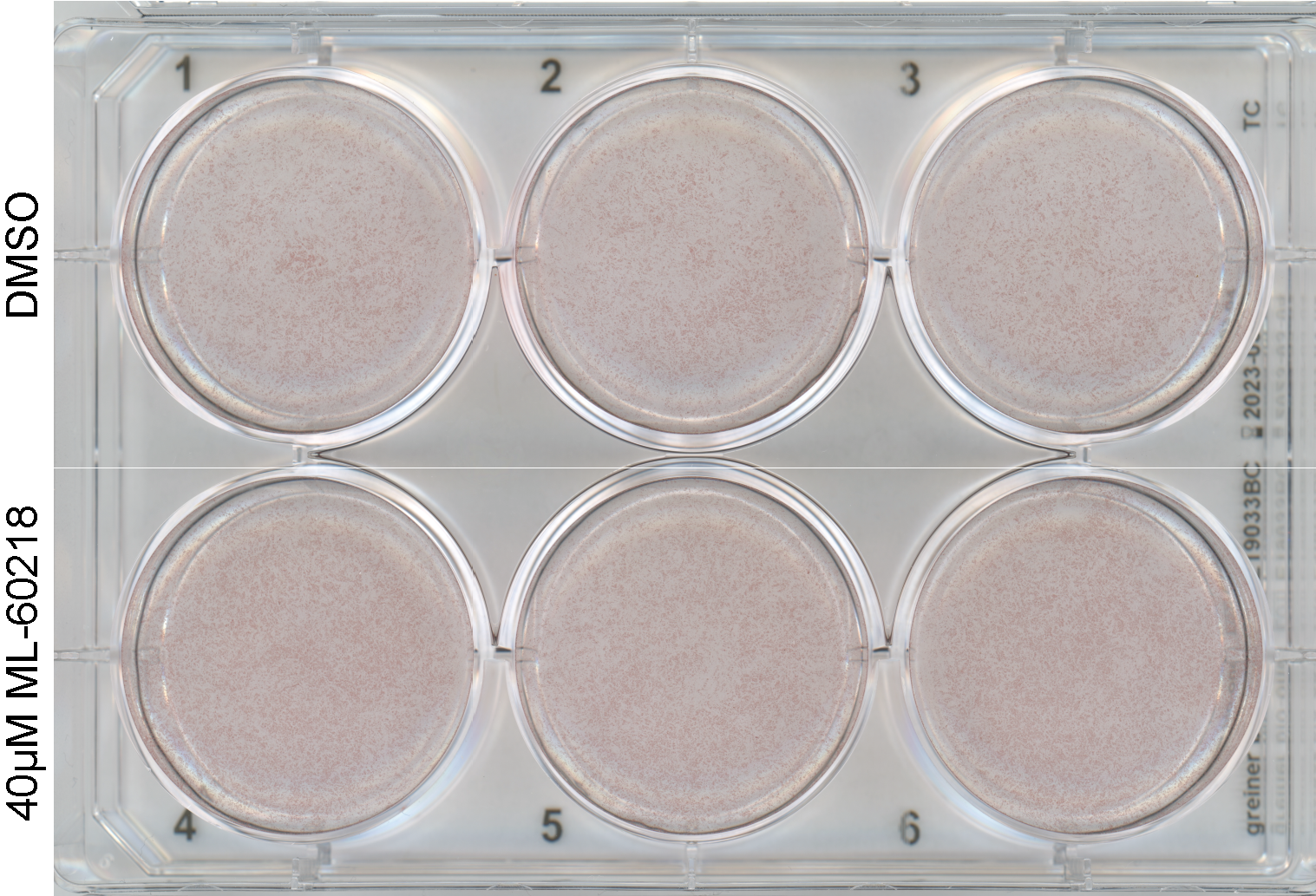

Supplement: Figure 4—figure supplement 2—source data 2. [file elife-74740-fig4-figsupp2-data2.zip › supplementary figure 8 D - source data/suppl fig 8D - source data1 - ORO.png]

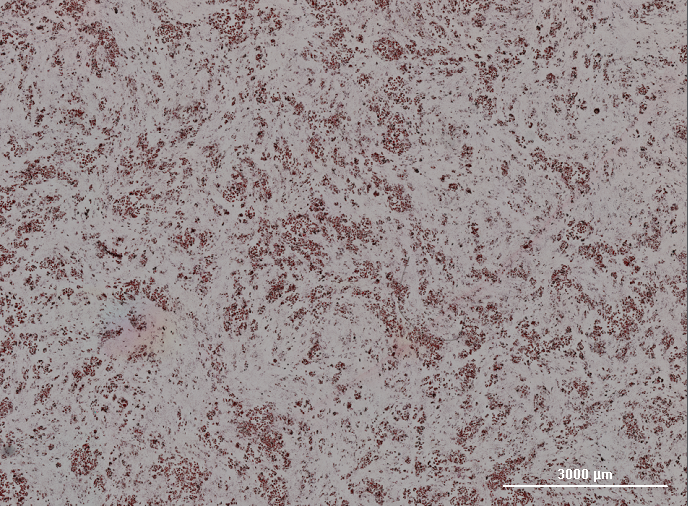

Supplement: Figure 4—figure supplement 2—source data 2. [file elife-74740-fig4-figsupp2-data2.zip › supplementary figure 8 D - source data/suppl fig 8D - source data10 - well 3 DMSO stitched.png]

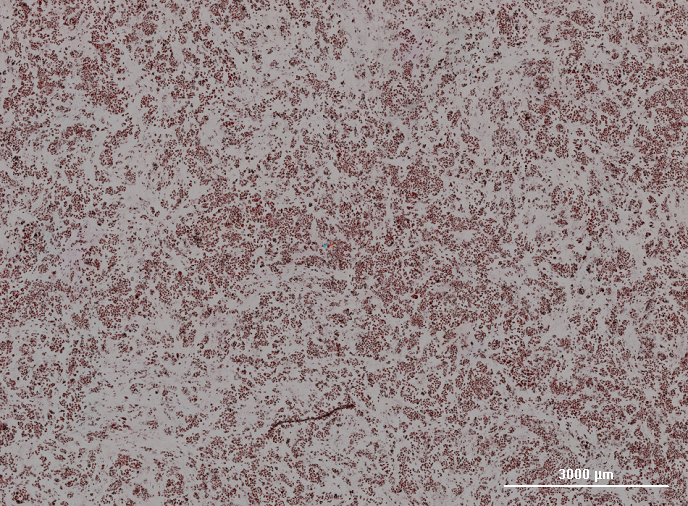

Supplement: Figure 4—figure supplement 2—source data 2. [file elife-74740-fig4-figsupp2-data2.zip › supplementary figure 8 D - source data/suppl fig 8D - source data11 - well 4 ML stitched.png]

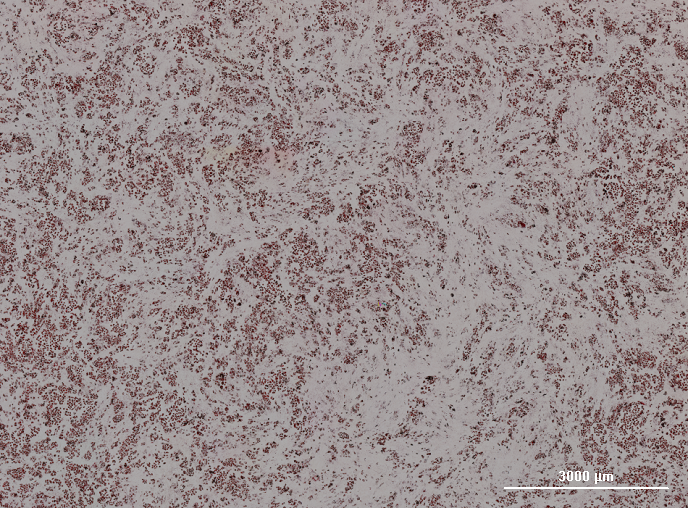

Supplement: Figure 4—figure supplement 2—source data 2. [file elife-74740-fig4-figsupp2-data2.zip › supplementary figure 8 D - source data/suppl fig 8D - source data12 - well 5 ML stitched.png]

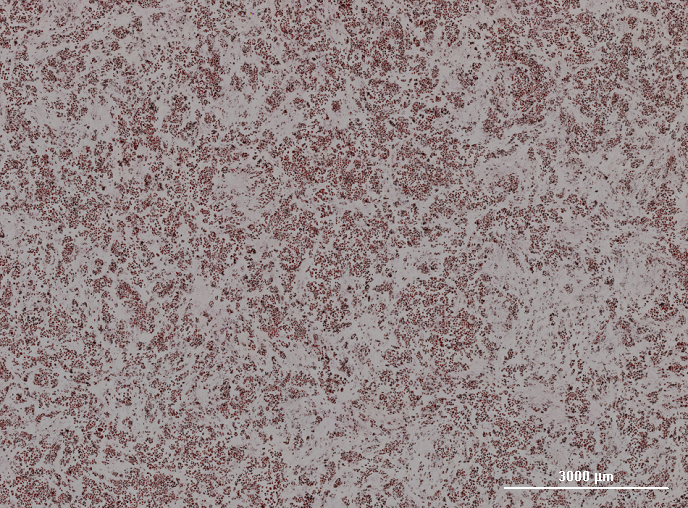

Supplement: Figure 4—figure supplement 2—source data 2. [file elife-74740-fig4-figsupp2-data2.zip › supplementary figure 8 D - source data/suppl fig 8D - source data13 - well 6 ML stitched.png]

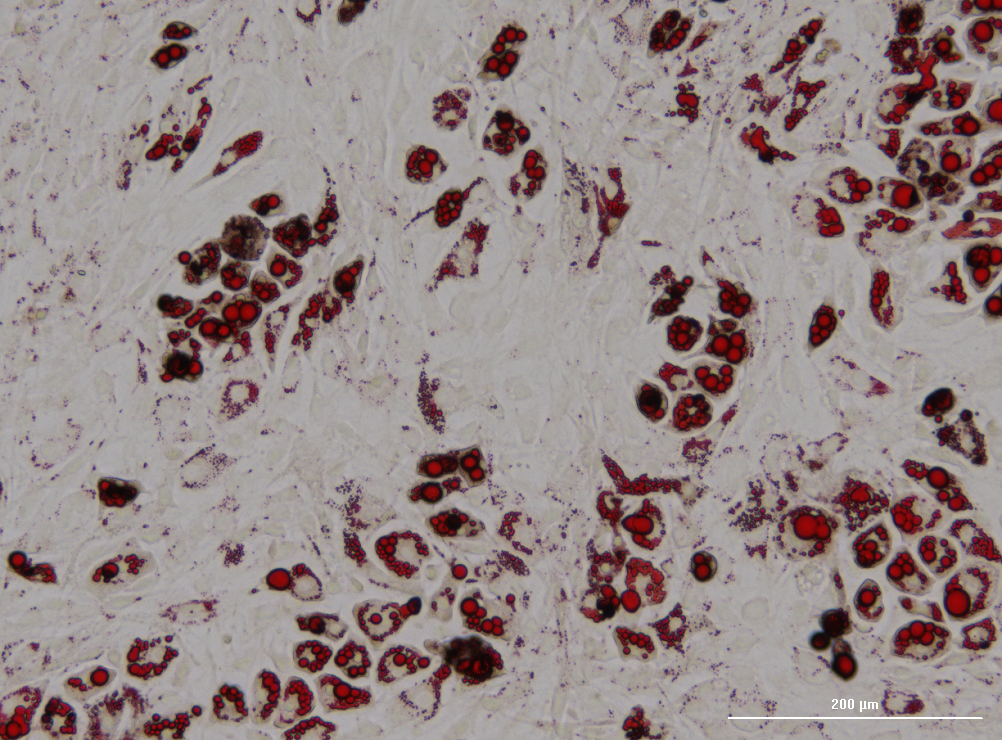

Supplement: Figure 4—figure supplement 2—source data 2. [file elife-74740-fig4-figsupp2-data2.zip › supplementary figure 8 D - source data/suppl fig 8D - source data2 - well 1 DMSO 10x.png]

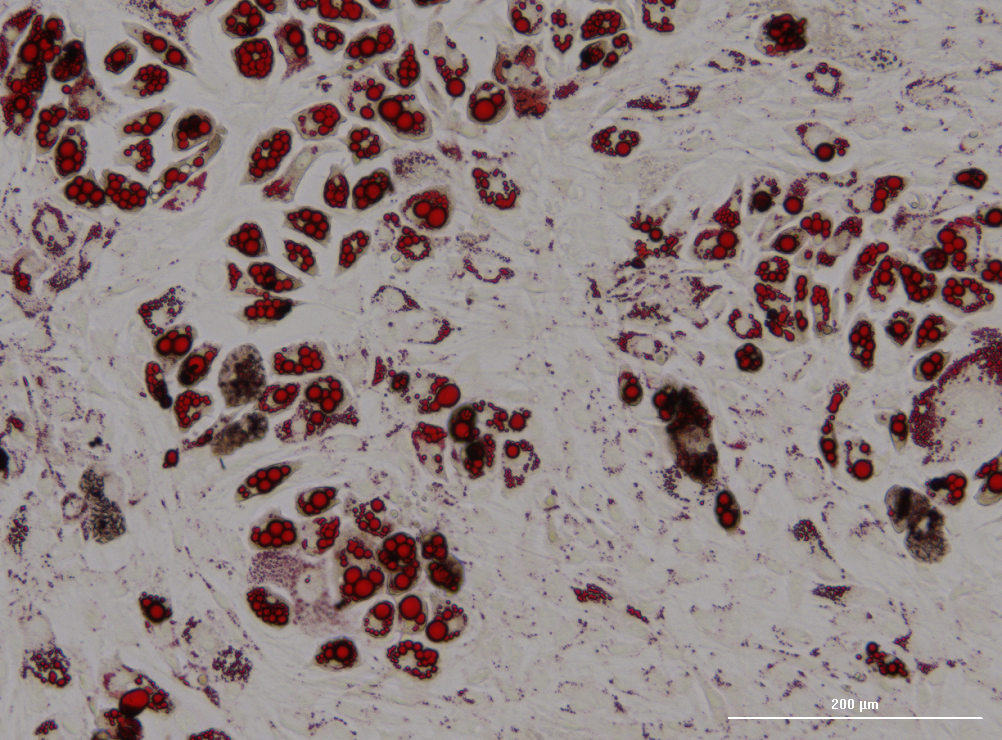

Supplement: Figure 4—figure supplement 2—source data 2. [file elife-74740-fig4-figsupp2-data2.zip › supplementary figure 8 D - source data/suppl fig 8D - source data3 - well 2 DMSO 10x.png]

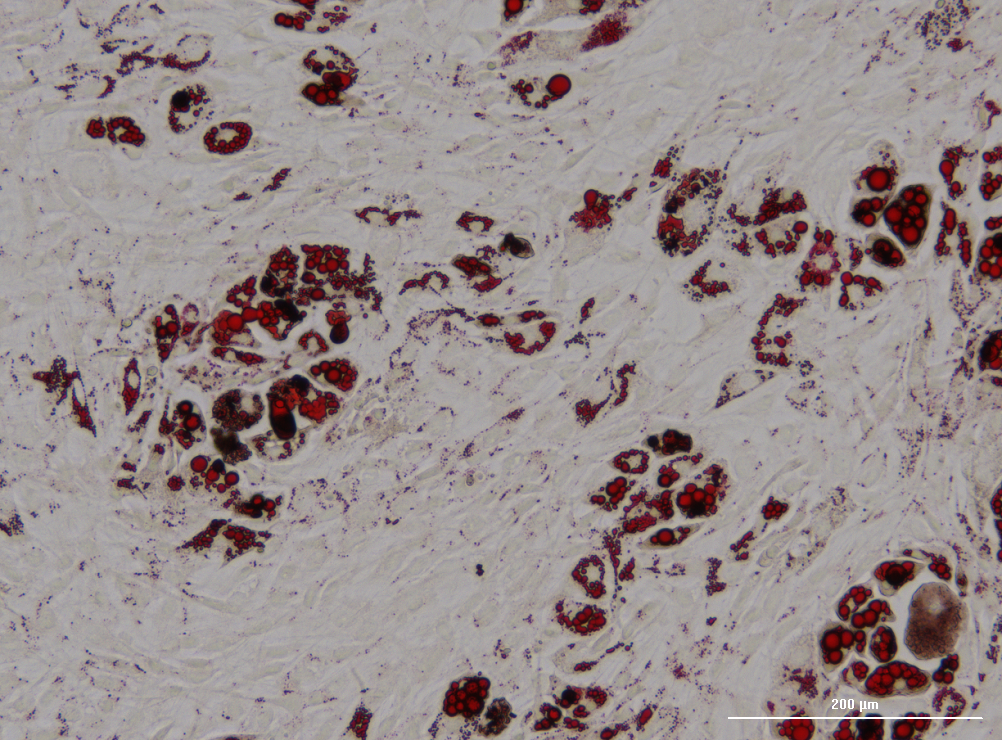

Supplement: Figure 4—figure supplement 2—source data 2. [file elife-74740-fig4-figsupp2-data2.zip › supplementary figure 8 D - source data/suppl fig 8D - source data4 - well 3 DMSO 10x.png]

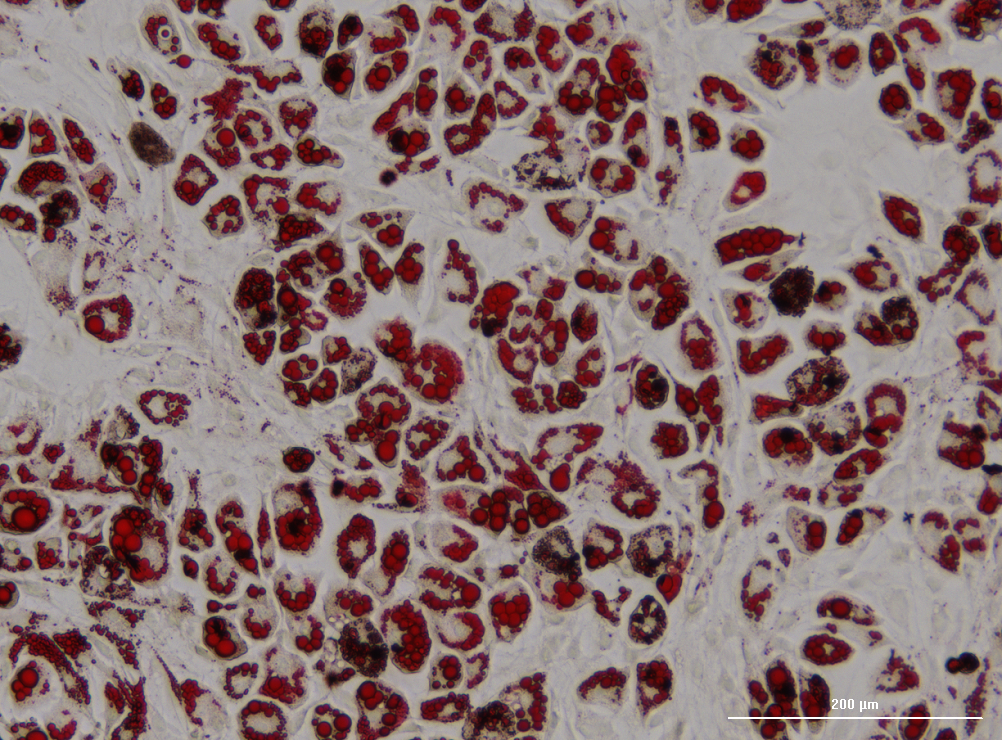

Supplement: Figure 4—figure supplement 2—source data 2. [file elife-74740-fig4-figsupp2-data2.zip › supplementary figure 8 D - source data/suppl fig 8D - source data5 - well 4 ML 10x.png]

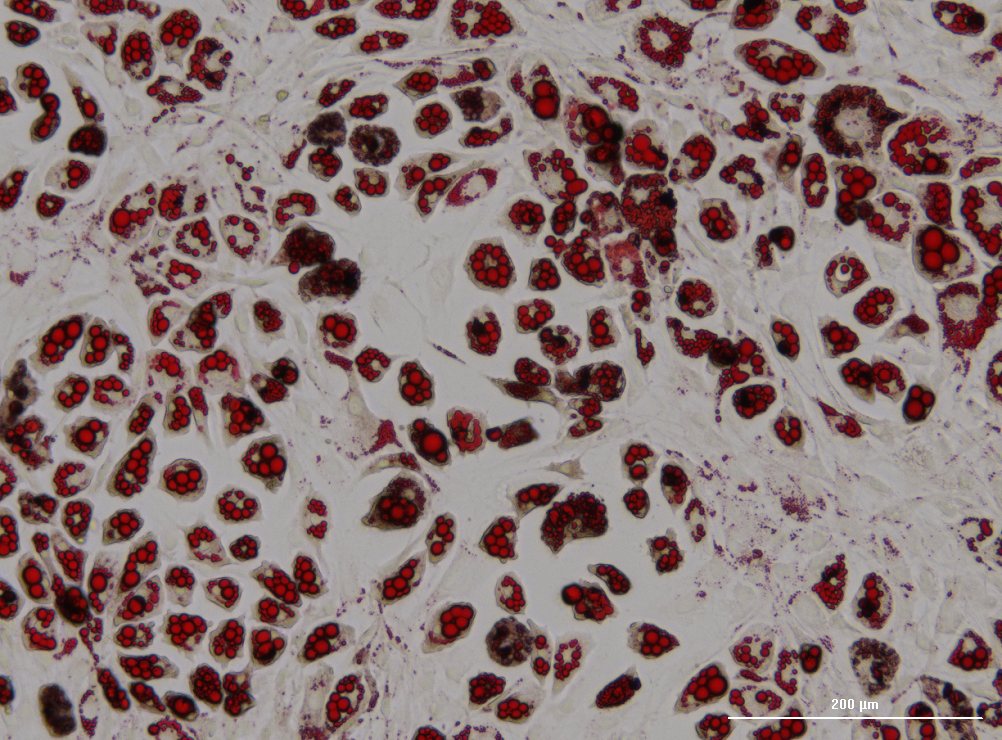

Supplement: Figure 4—figure supplement 2—source data 2. [file elife-74740-fig4-figsupp2-data2.zip › supplementary figure 8 D - source data/suppl fig 8D - source data6 - well 5 ML 10x.png]

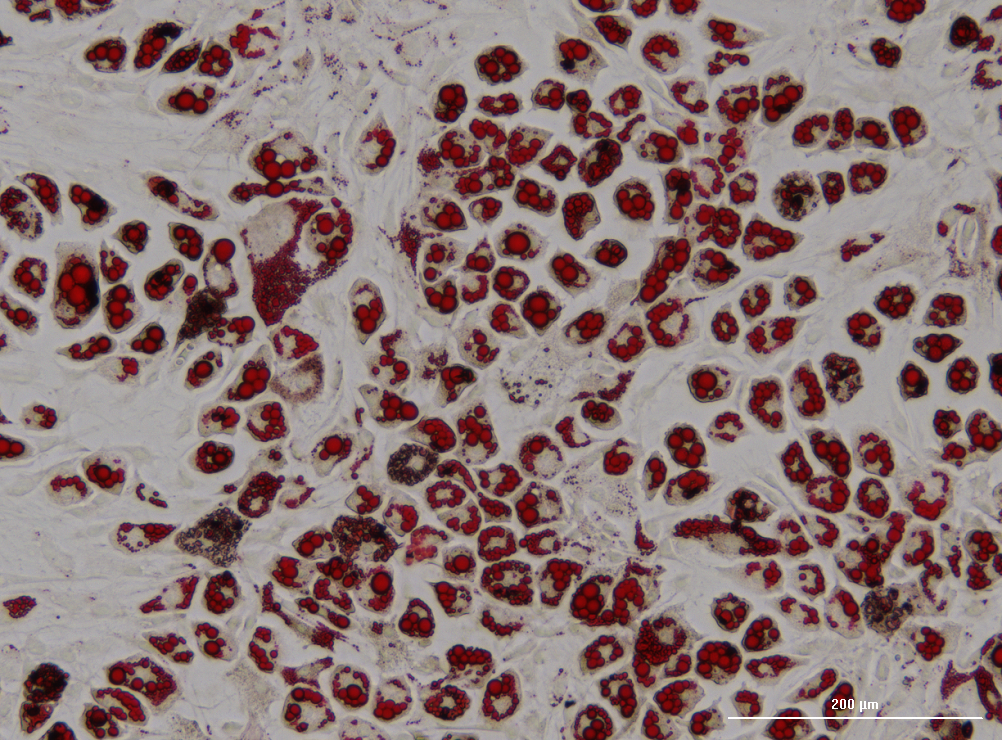

Supplement: Figure 4—figure supplement 2—source data 2. [file elife-74740-fig4-figsupp2-data2.zip › supplementary figure 8 D - source data/suppl fig 8D - source data7 - well 6 ML 10x.png]

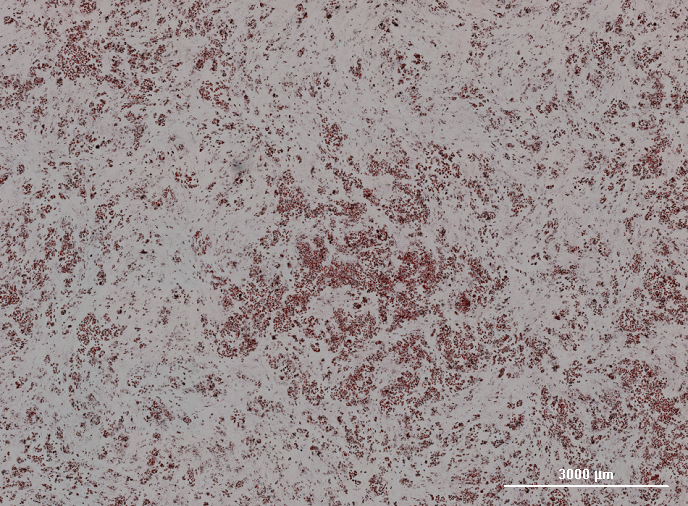

Supplement: Figure 4—figure supplement 2—source data 2. [file elife-74740-fig4-figsupp2-data2.zip › supplementary figure 8 D - source data/suppl fig 8D - source data8 - well 1 DMSO stitched.png]

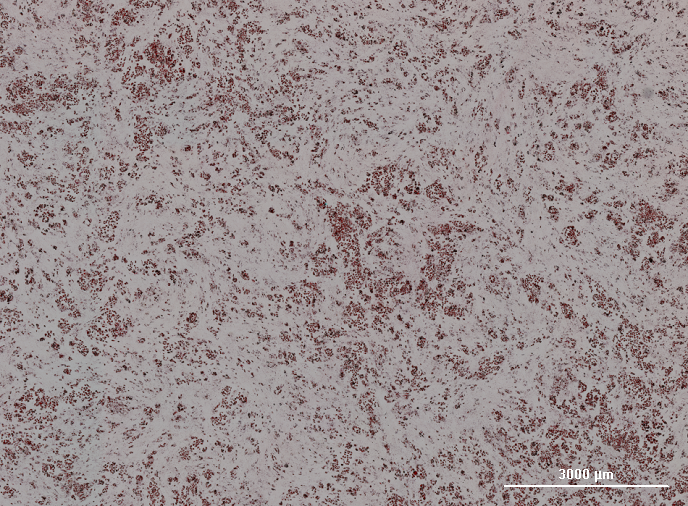

Supplement: Figure 4—figure supplement 2—source data 2. [file elife-74740-fig4-figsupp2-data2.zip › supplementary figure 8 D - source data/suppl fig 8D - source data9 - well 2 DMSO stitched.png]

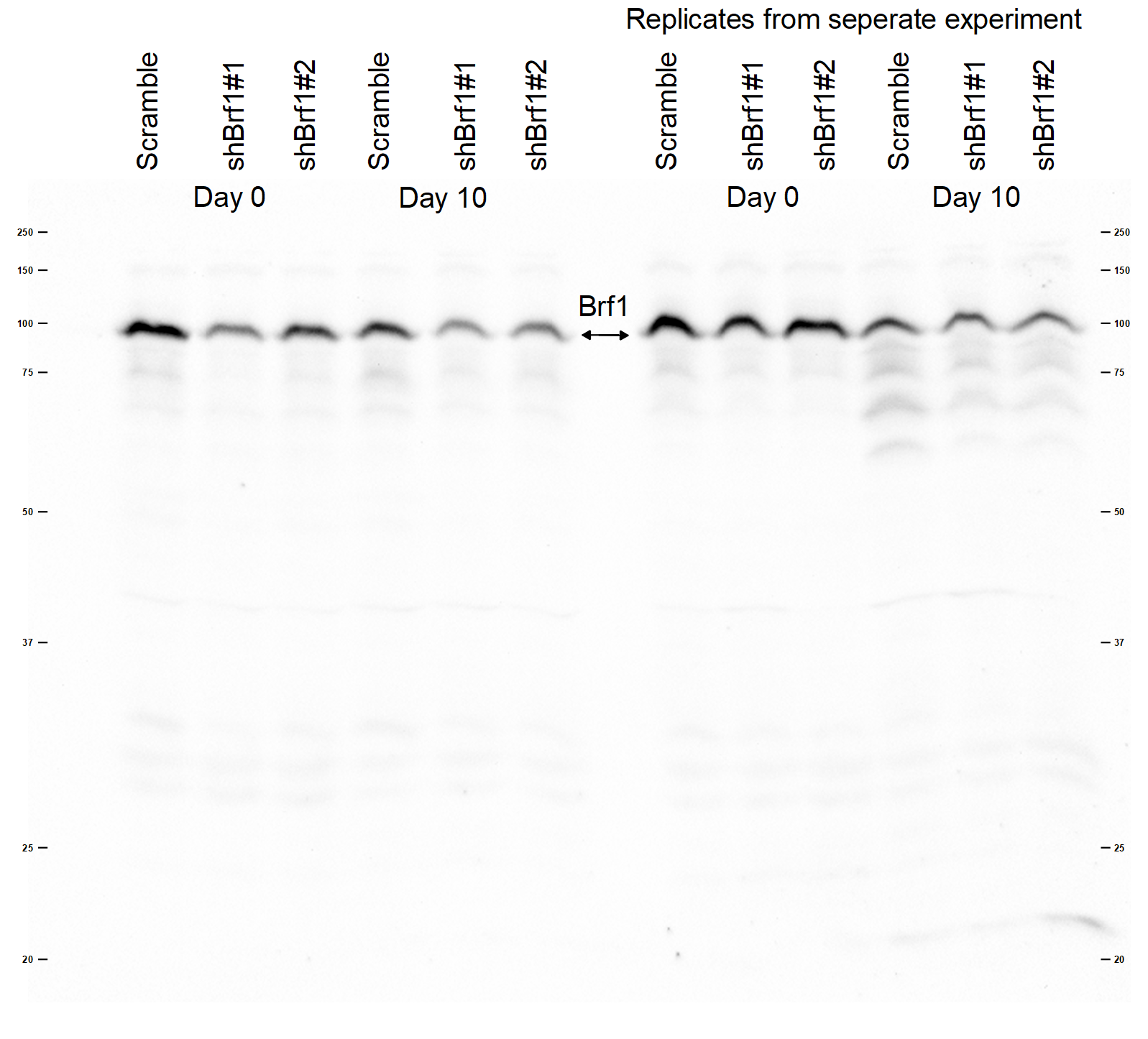

Supplement: Figure 5—source data 1. [file elife-74740-fig5-data1.zip › Figure 5A - source data/figure 5A - source data 1 Brf1 labeled.tif]

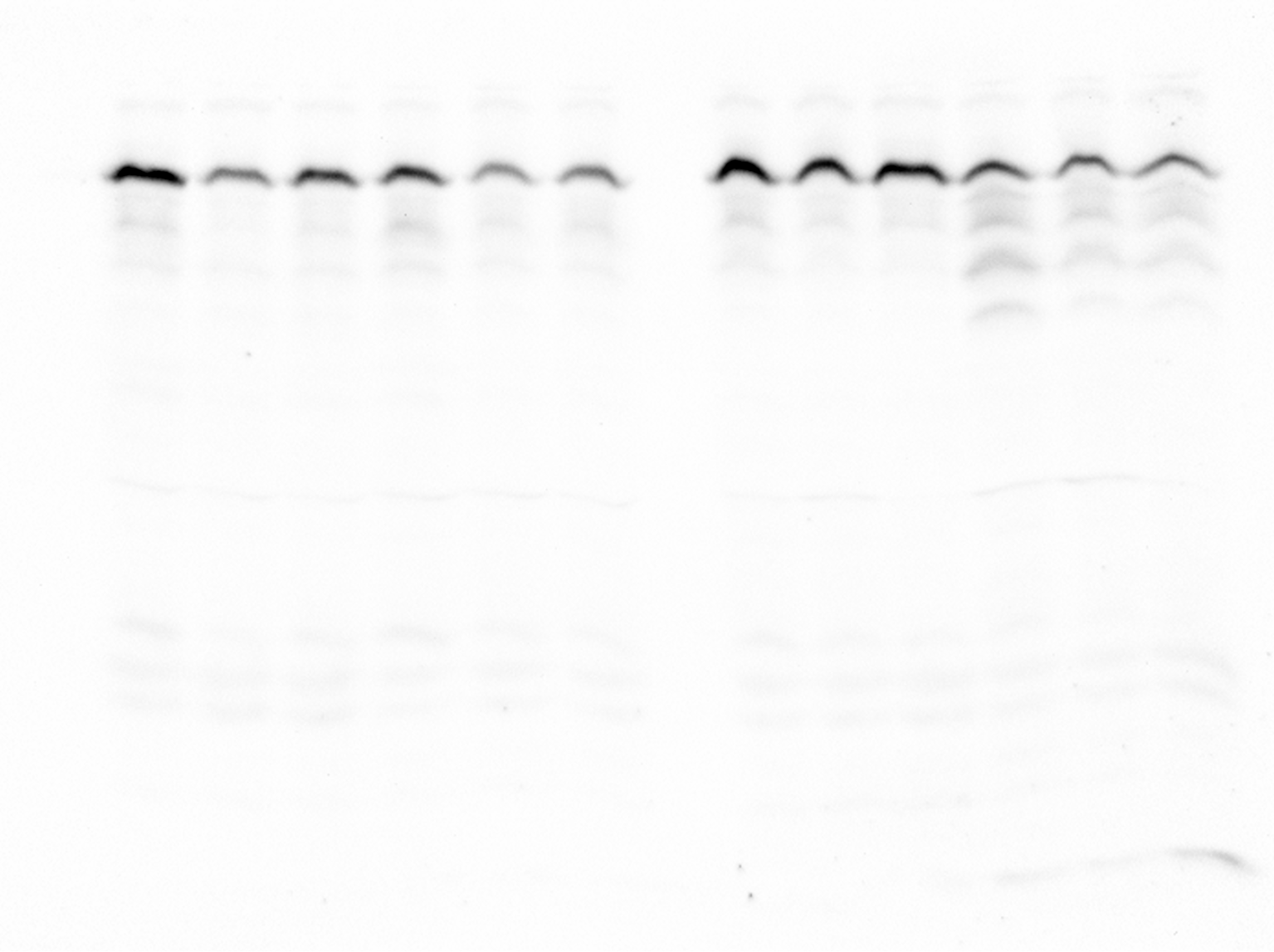

Supplement: Figure 5—source data 1. [file elife-74740-fig5-data1.zip › Figure 5A - source data/figure 5A - source data 2 Brf1.tif]

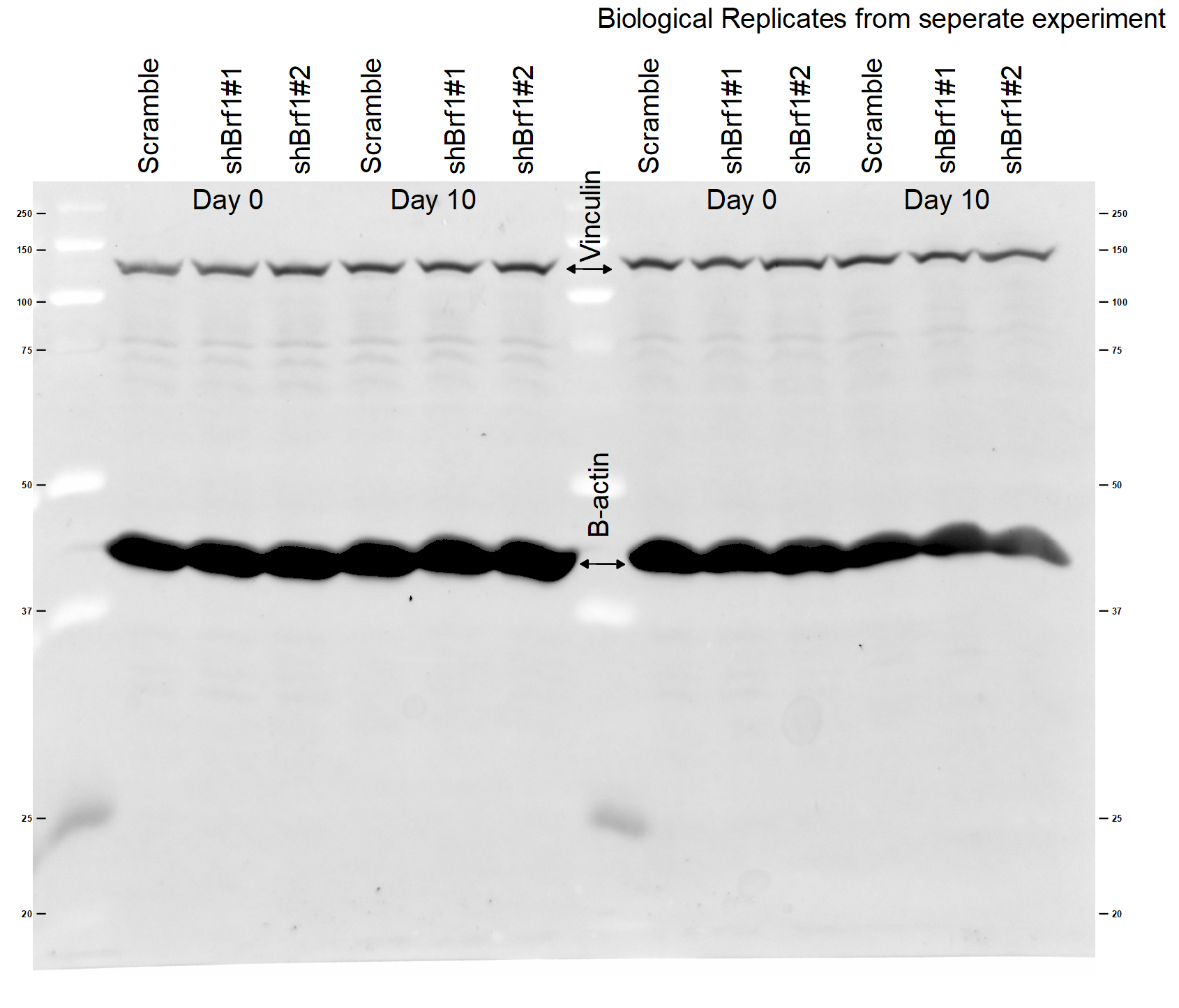

Supplement: Figure 5—source data 1. [file elife-74740-fig5-data1.zip › Figure 5A - source data/figure 5A - source data 3 Vinculin labeled.tif]

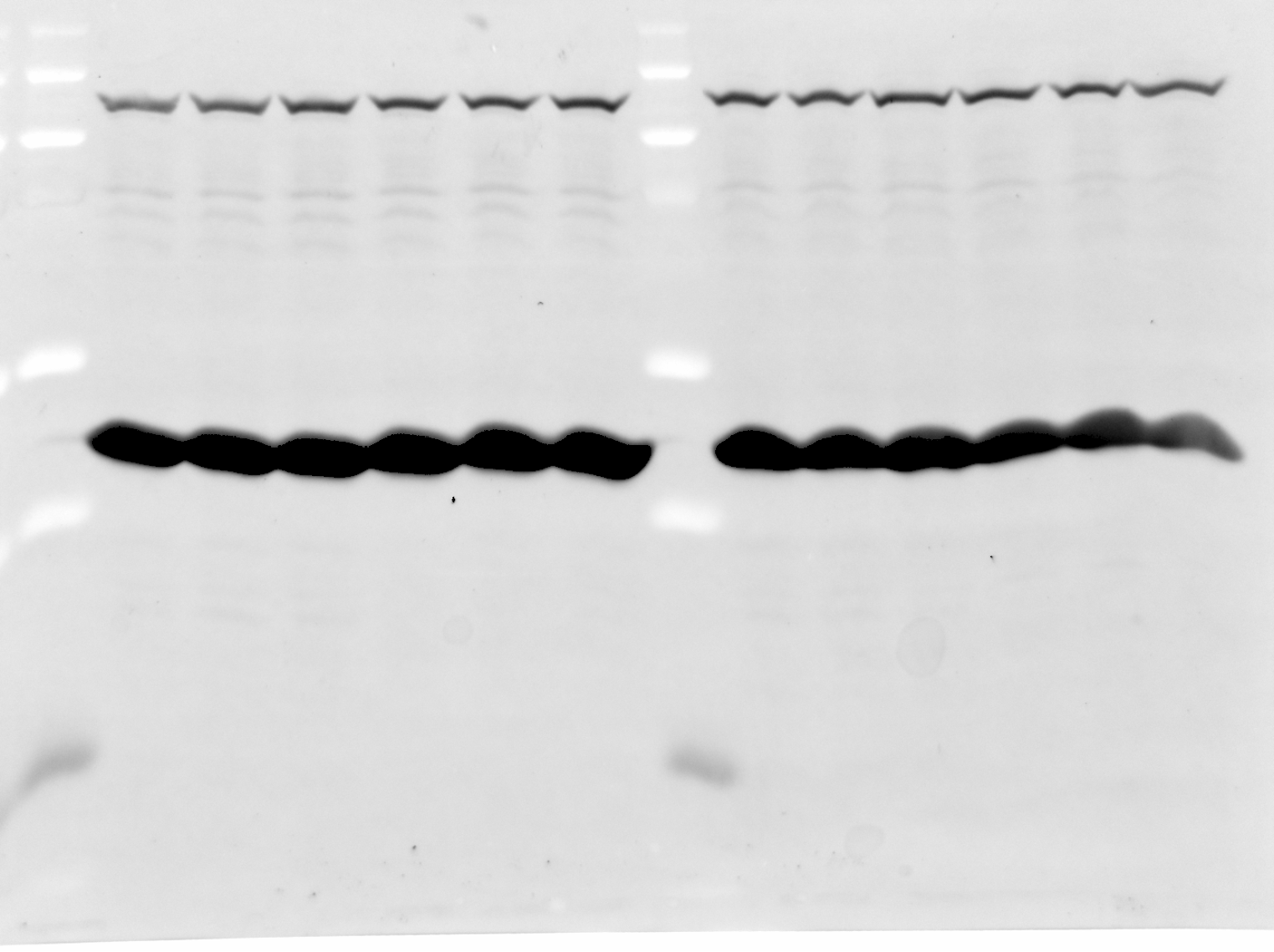

Supplement: Figure 5—source data 1. [file elife-74740-fig5-data1.zip › Figure 5A - source data/figure 5A - source data 4 Vinculin.tif]

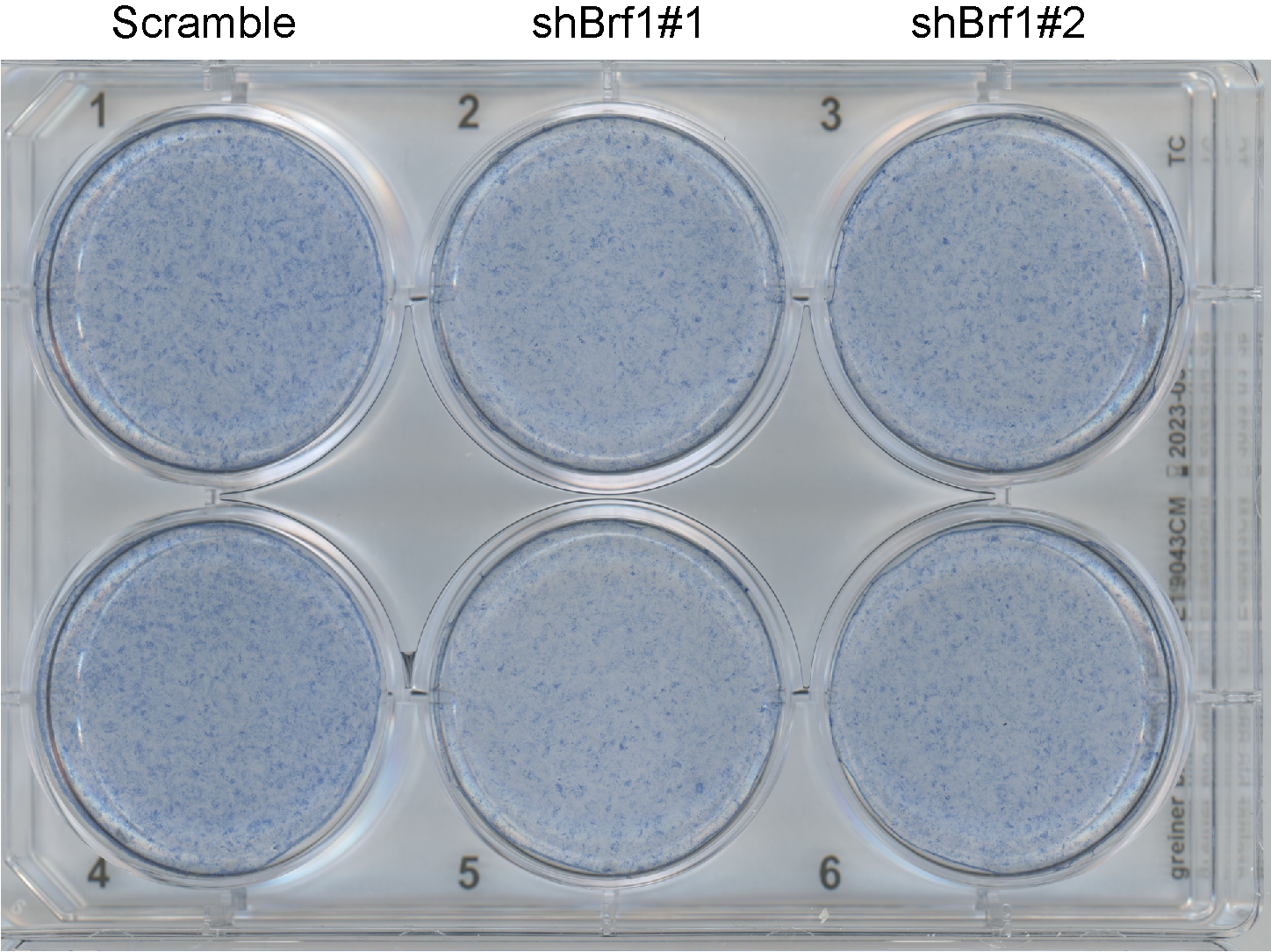

Supplement: Figure 5—source data 2. [file elife-74740-fig5-data2.zip › figure 5 C-D - source data/Figure 5 C - source data 1 - Alp.png]

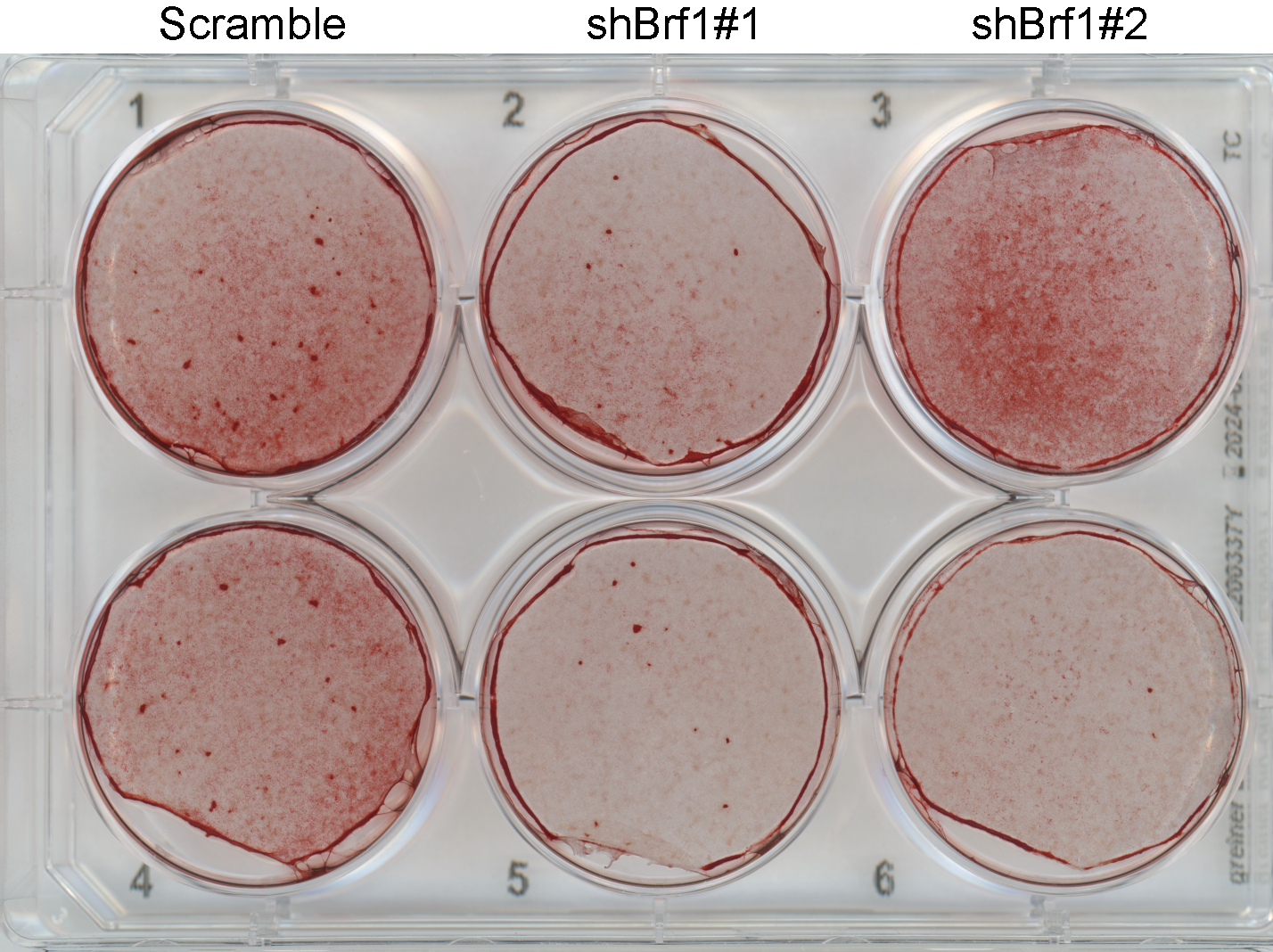

Supplement: Figure 5—source data 2. [file elife-74740-fig5-data2.zip › figure 5 C-D - source data/figure 5D - source data 2 - Alizarin red.png]

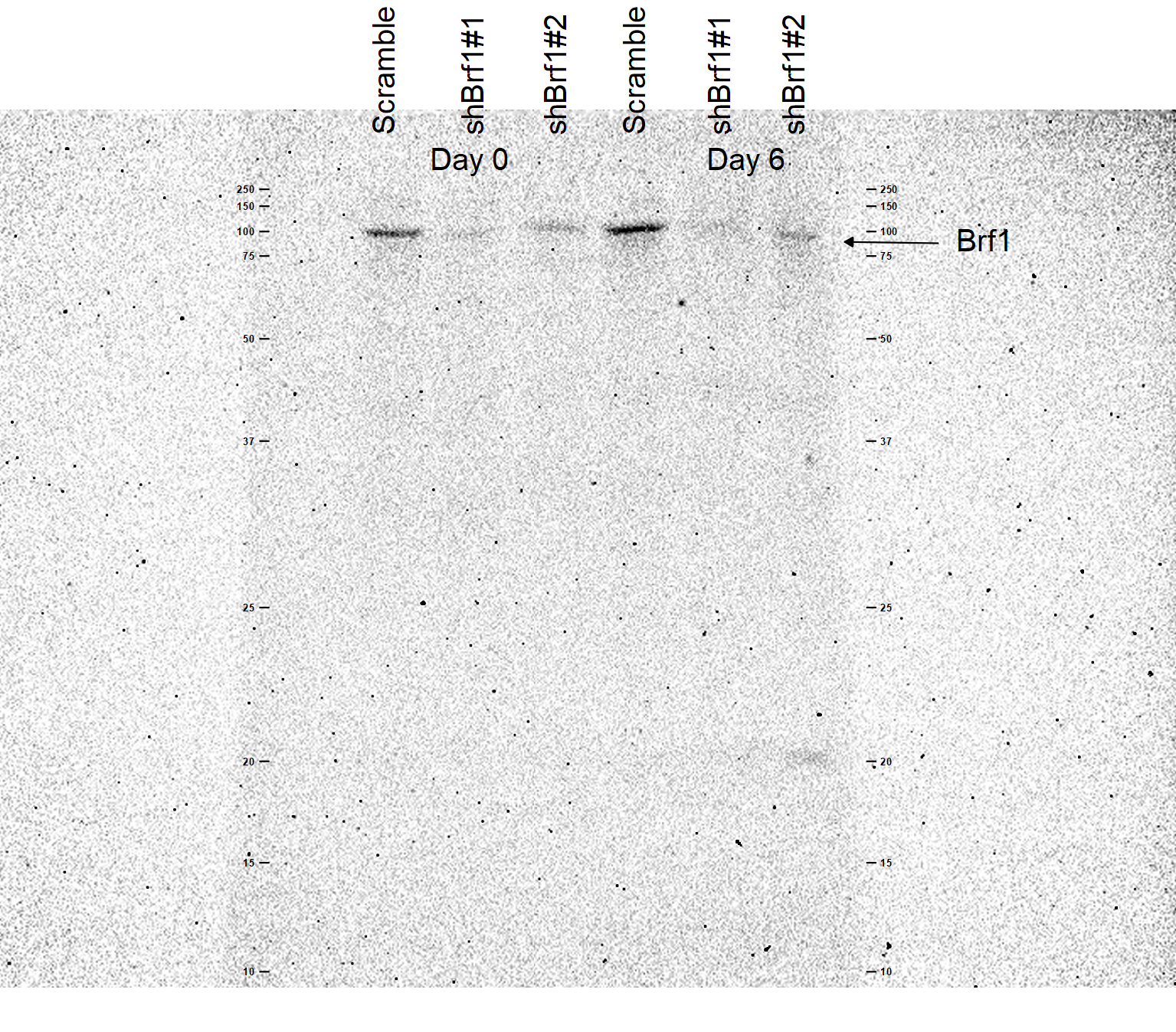

Supplement: Figure 5—figure supplement 1—source data 1. [file elife-74740-fig5-figsupp1-data1.zip › supplementary figure 9 A - source data/supplementary figure 9A source data 1 Brf1 labeled.tif]

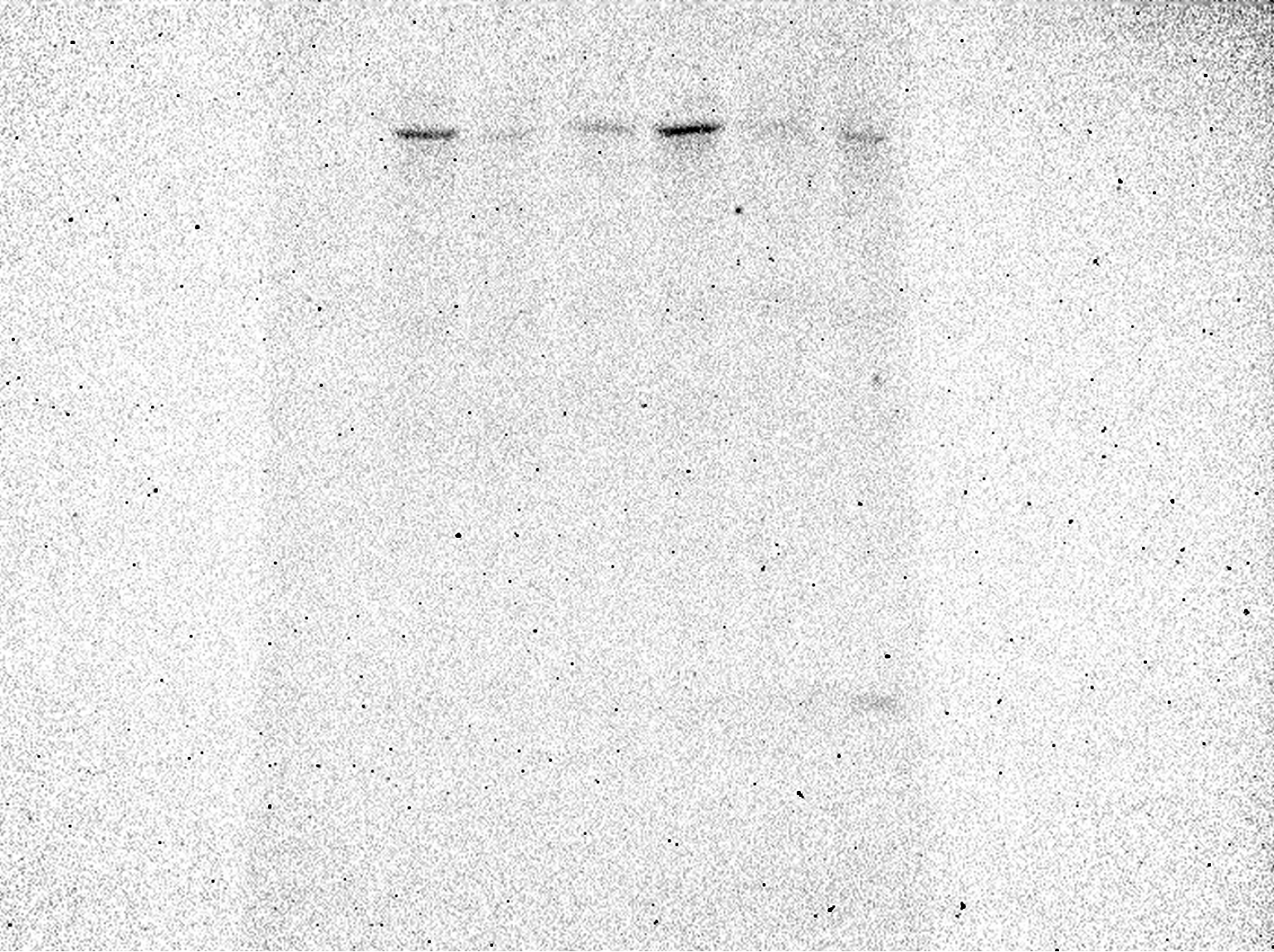

Supplement: Figure 5—figure supplement 1—source data 1. [file elife-74740-fig5-figsupp1-data1.zip › supplementary figure 9 A - source data/supplementary figure 9A source data 2 Brf1.tif]

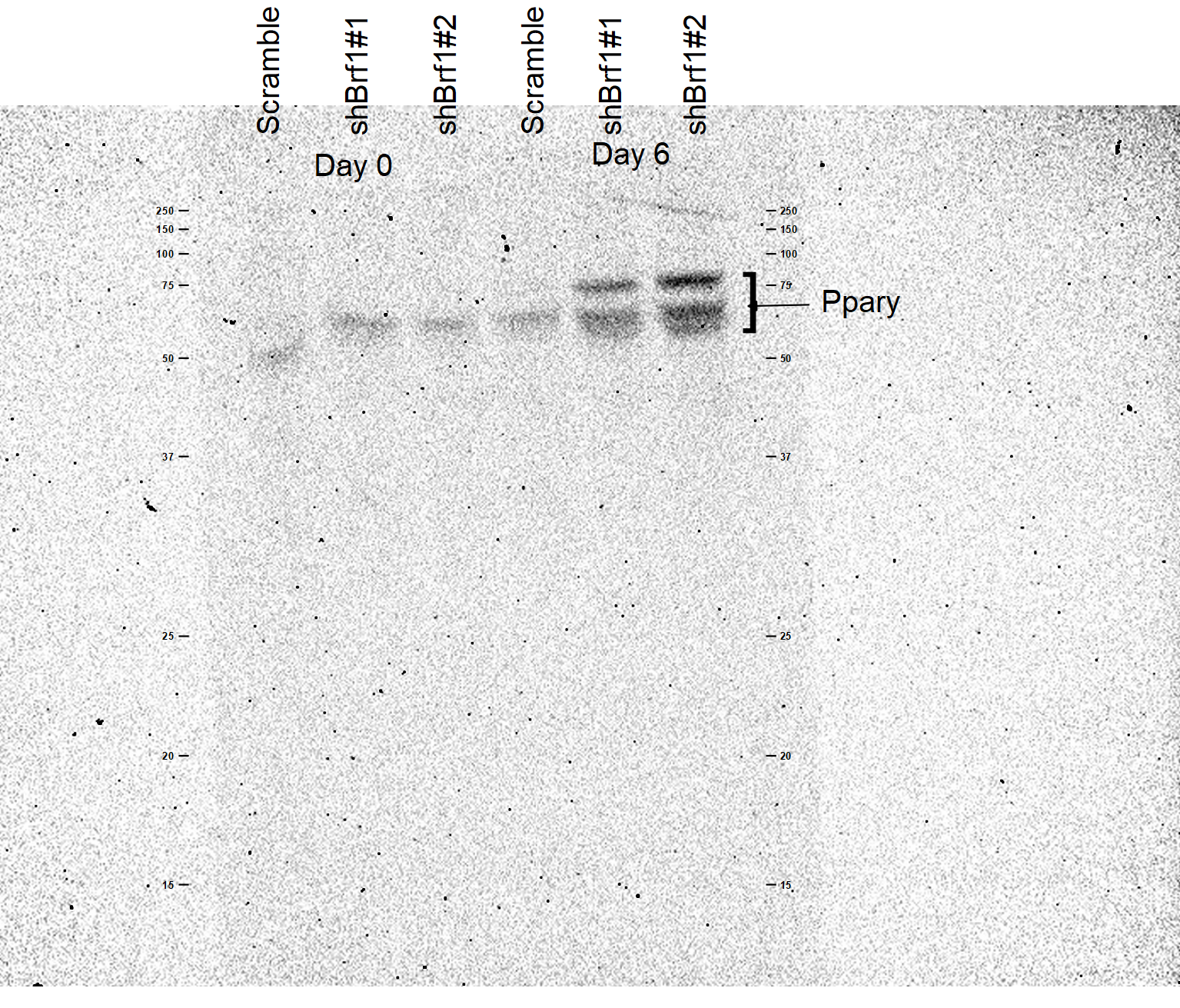

Supplement: Figure 5—figure supplement 1—source data 1. [file elife-74740-fig5-figsupp1-data1.zip › supplementary figure 9 A - source data/supplementary figure 9A source data 3 Ppary labeled.tif]

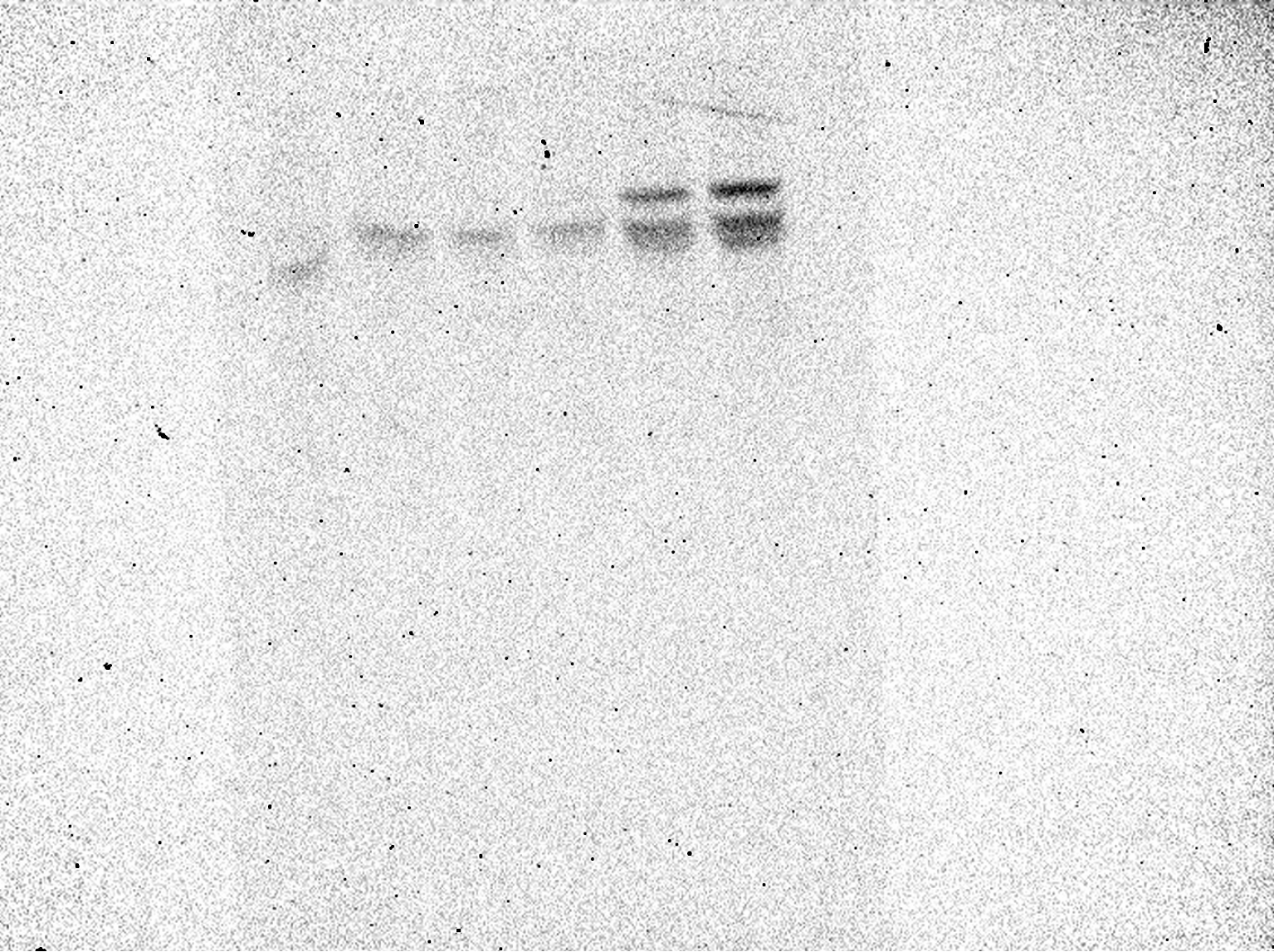

Supplement: Figure 5—figure supplement 1—source data 1. [file elife-74740-fig5-figsupp1-data1.zip › supplementary figure 9 A - source data/supplementary figure 9A source data 4 Ppary.tif]

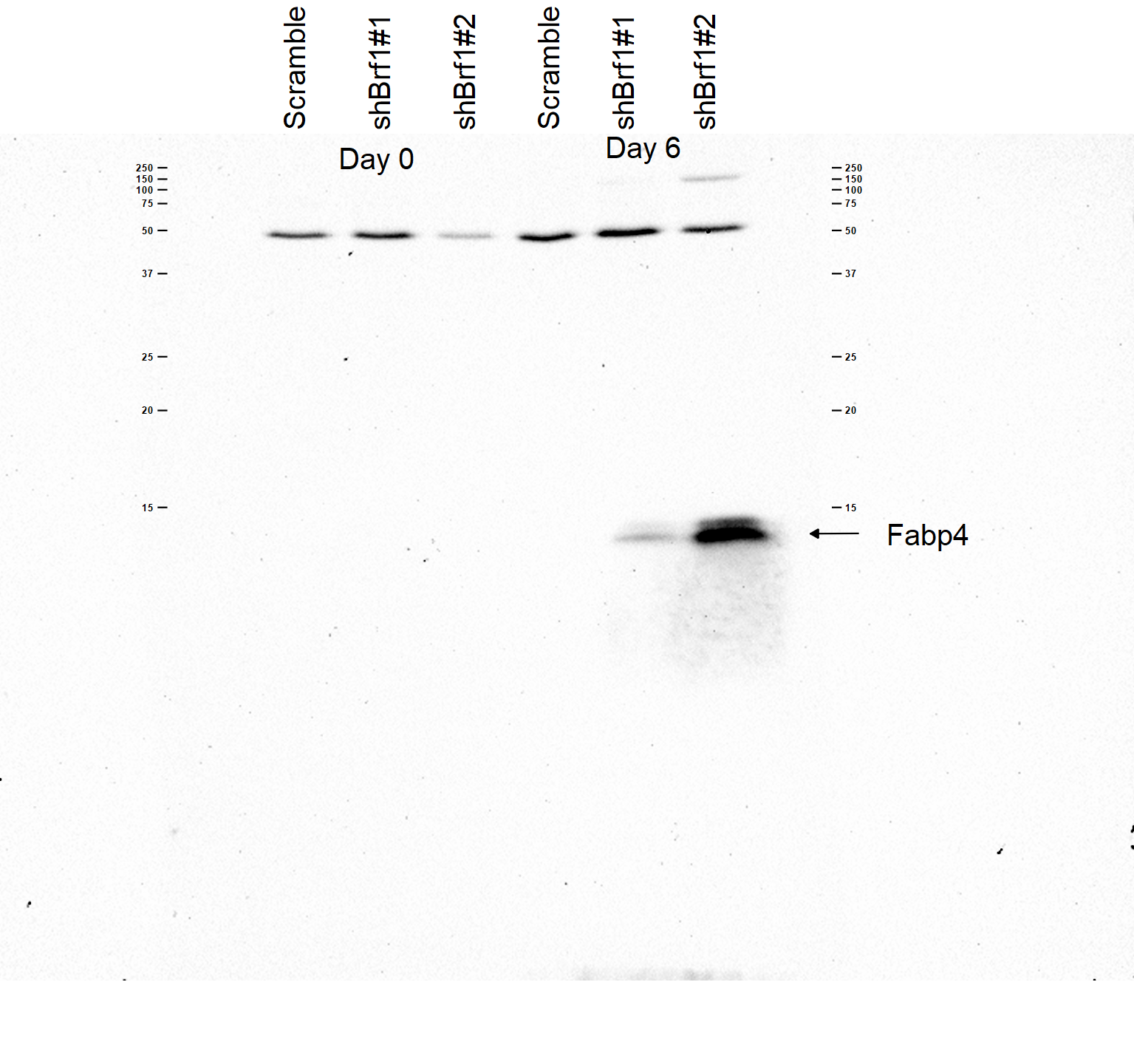

Supplement: Figure 5—figure supplement 1—source data 1. [file elife-74740-fig5-figsupp1-data1.zip › supplementary figure 9 A - source data/supplementary figure 9A source data 5 Fabp4 labeled.tif]

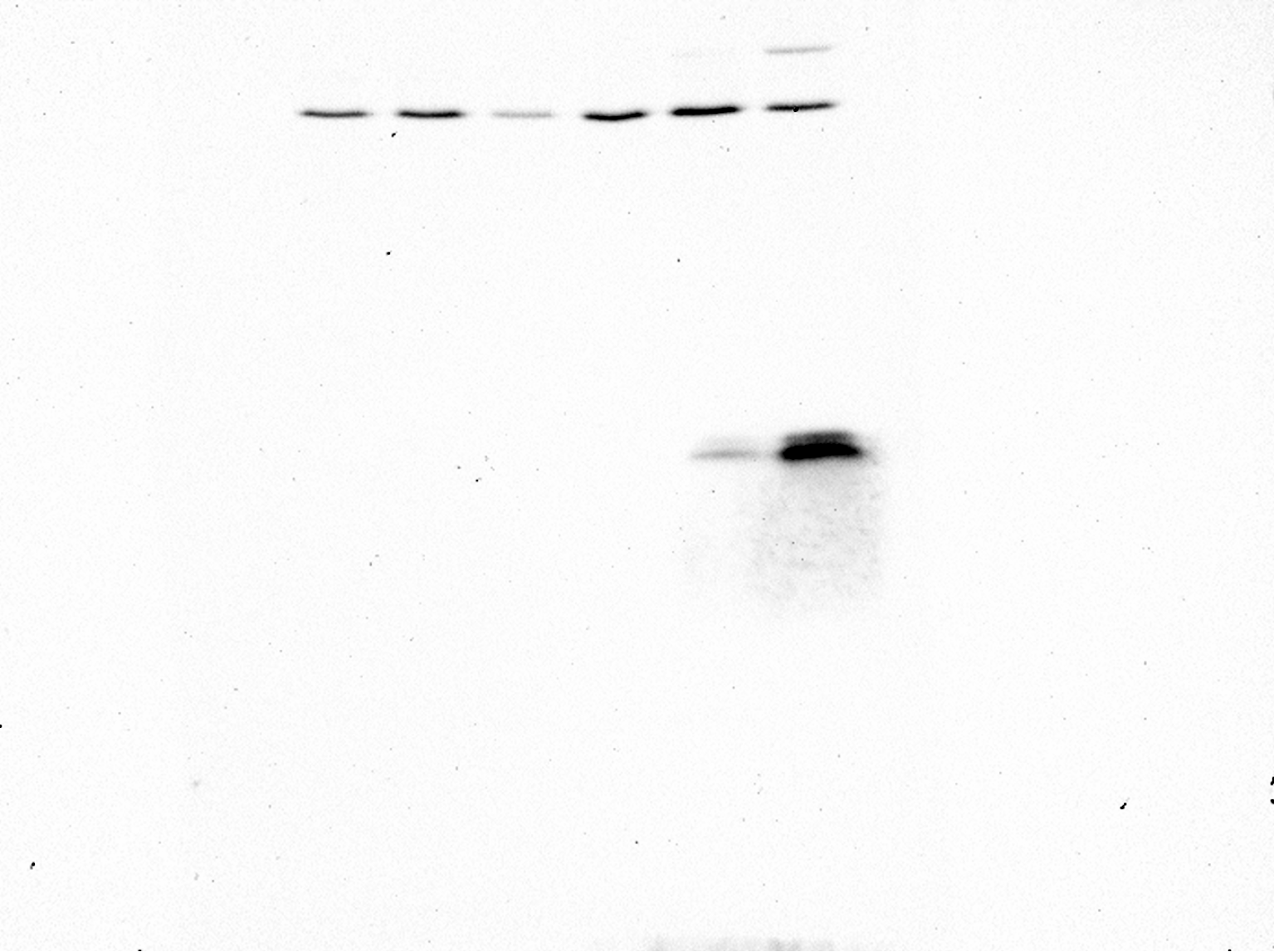

Supplement: Figure 5—figure supplement 1—source data 1. [file elife-74740-fig5-figsupp1-data1.zip › supplementary figure 9 A - source data/supplementary figure 9A source data 6 Fabp4.tif]

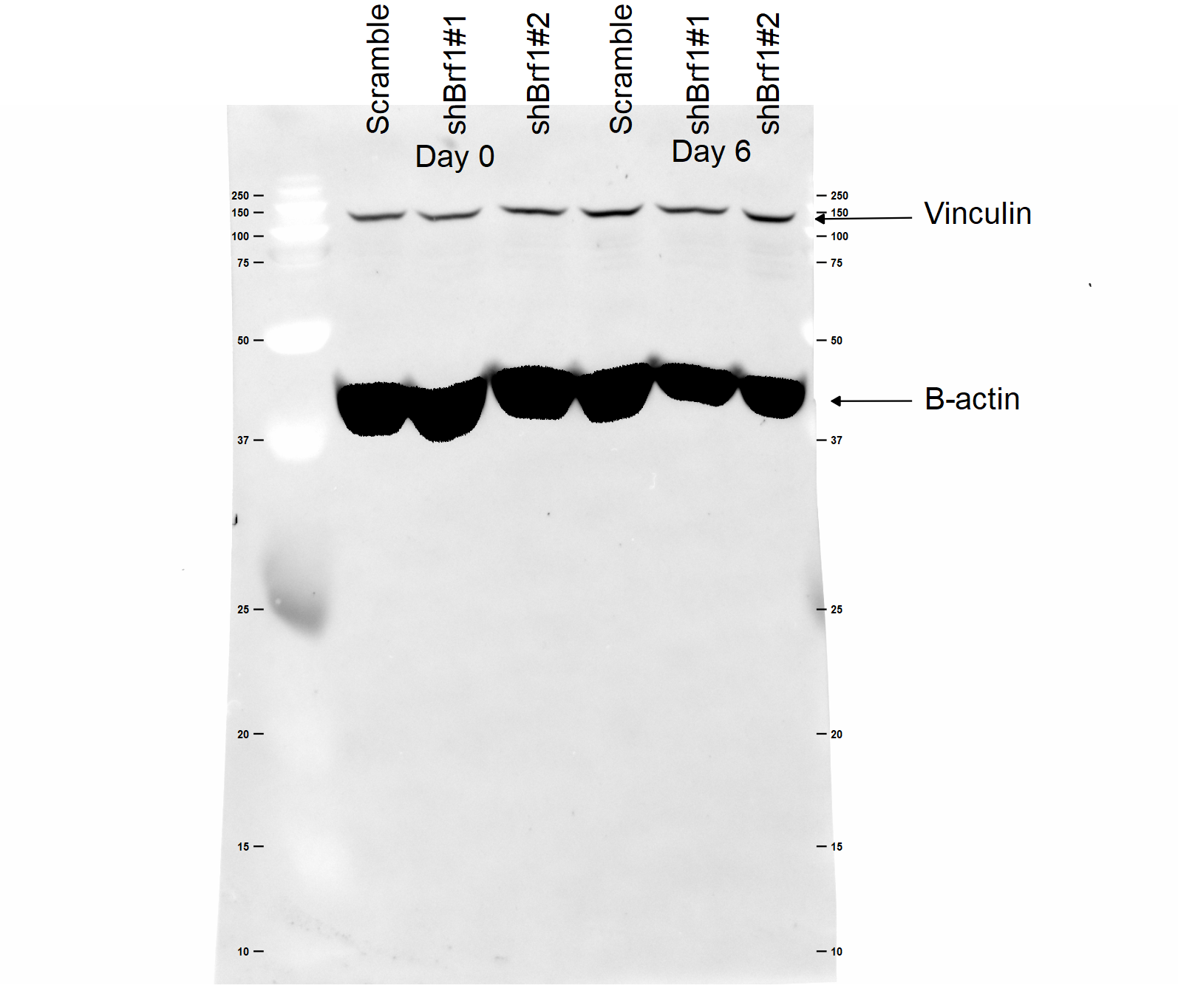

Supplement: Figure 5—figure supplement 1—source data 1. [file elife-74740-fig5-figsupp1-data1.zip › supplementary figure 9 A - source data/supplementary figure 9A source data 7 vinculin labeled.tif]

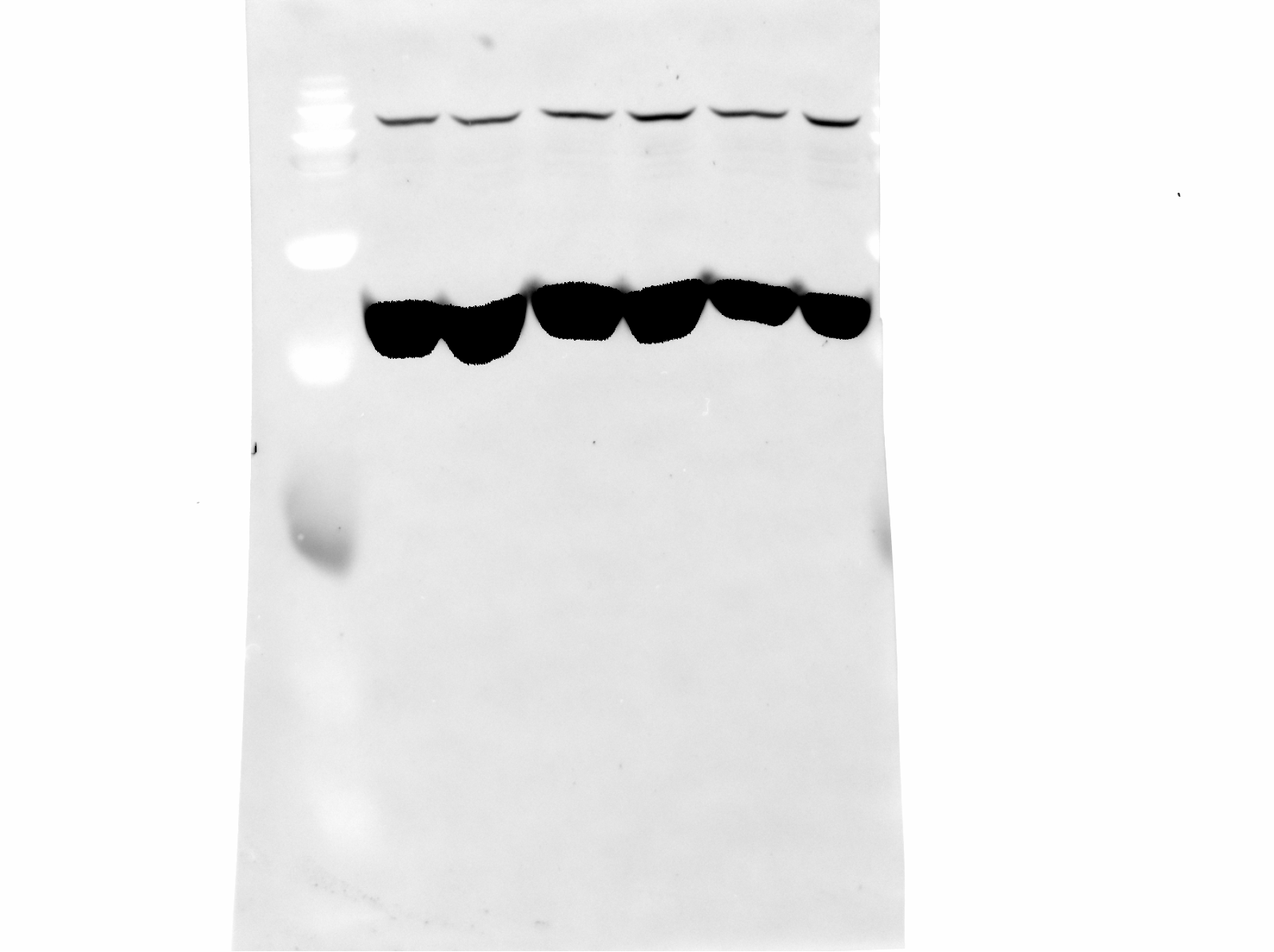

Supplement: Figure 5—figure supplement 1—source data 1. [file elife-74740-fig5-figsupp1-data1.zip › supplementary figure 9 A - source data/supplementary figure 9A source data 8 vinculin.tif]

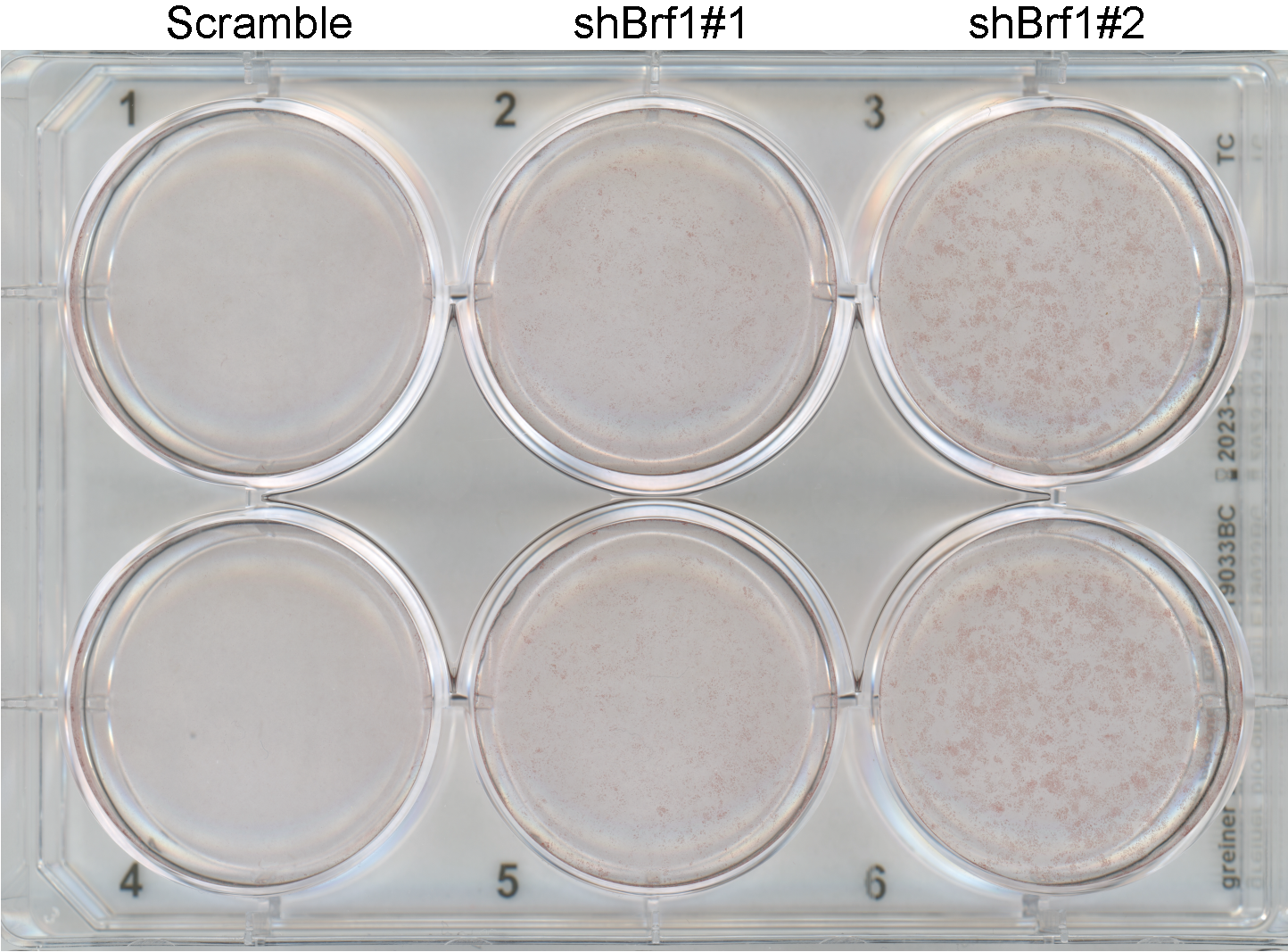

Supplement: Figure 5—figure supplement 1—source data 2. [file elife-74740-fig5-figsupp1-data2.zip › supplementary figure 9 D - source data/suppl fig 9D - source data 1 - ORO.png]

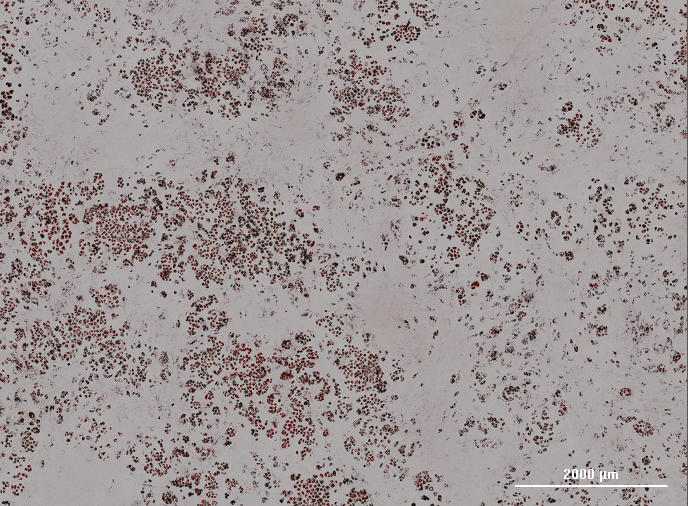

Supplement: Figure 5—figure supplement 1—source data 2. [file elife-74740-fig5-figsupp1-data2.zip › supplementary figure 9 D - source data/suppl fig 9D - source data 10 - well 3 shBrf1-2 stitched.png]

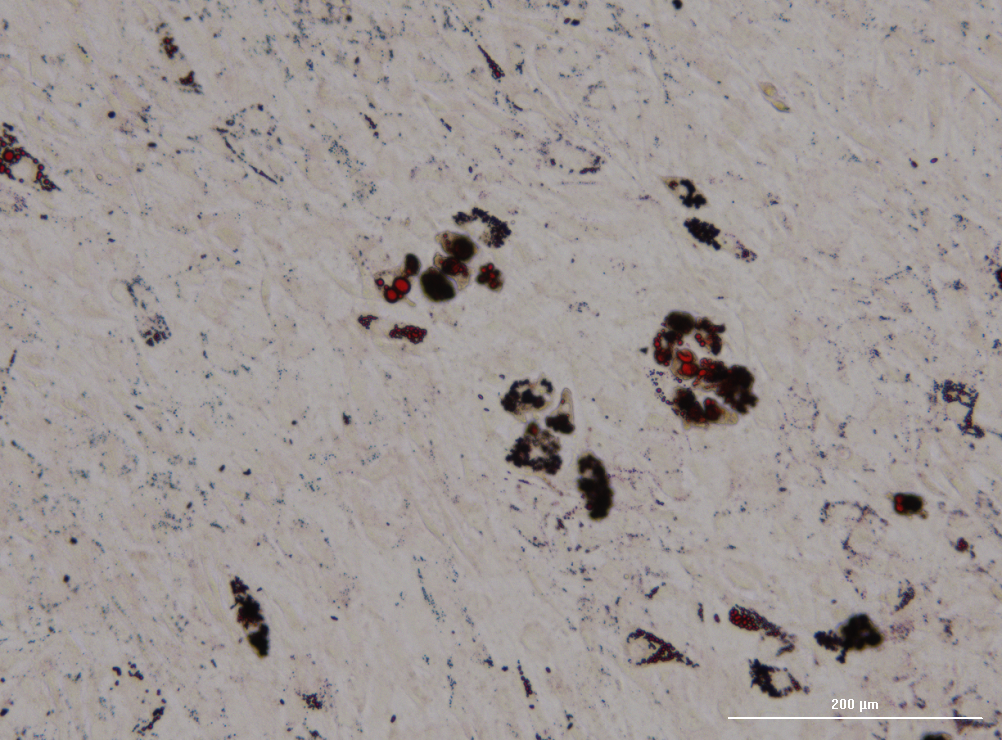

Supplement: Figure 5—figure supplement 1—source data 2. [file elife-74740-fig5-figsupp1-data2.zip › supplementary figure 9 D - source data/suppl fig 9D - source data 2 - well 1 SCR 10x.png]

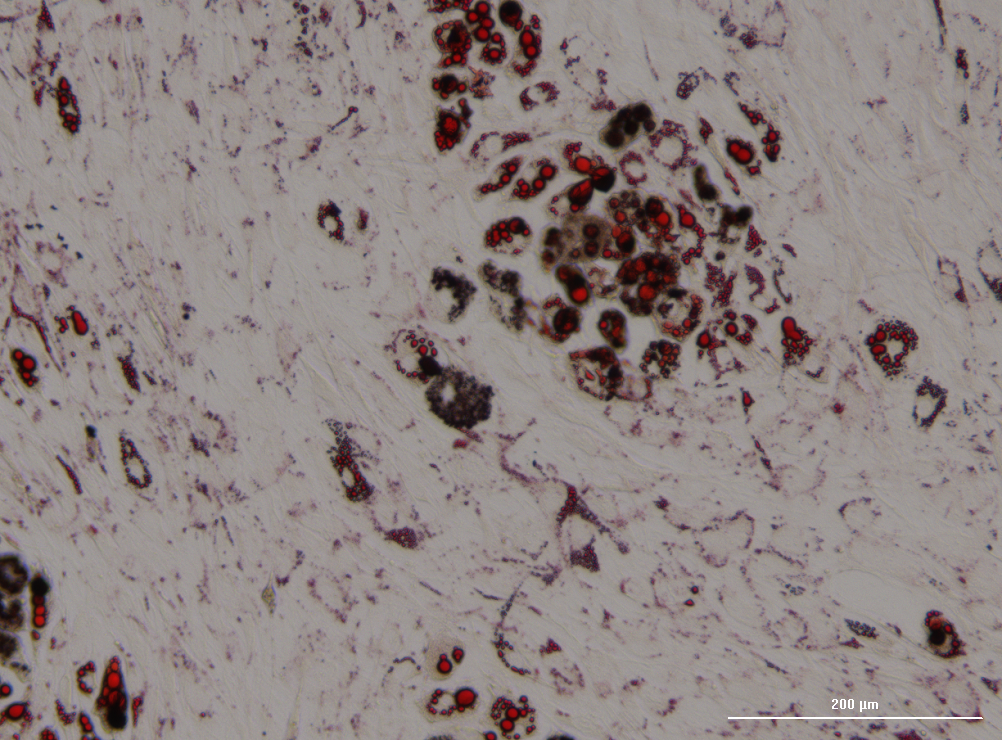

Supplement: Figure 5—figure supplement 1—source data 2. [file elife-74740-fig5-figsupp1-data2.zip › supplementary figure 9 D - source data/suppl fig 9D - source data 3 - well 2 shBrf1-1 10x.png]

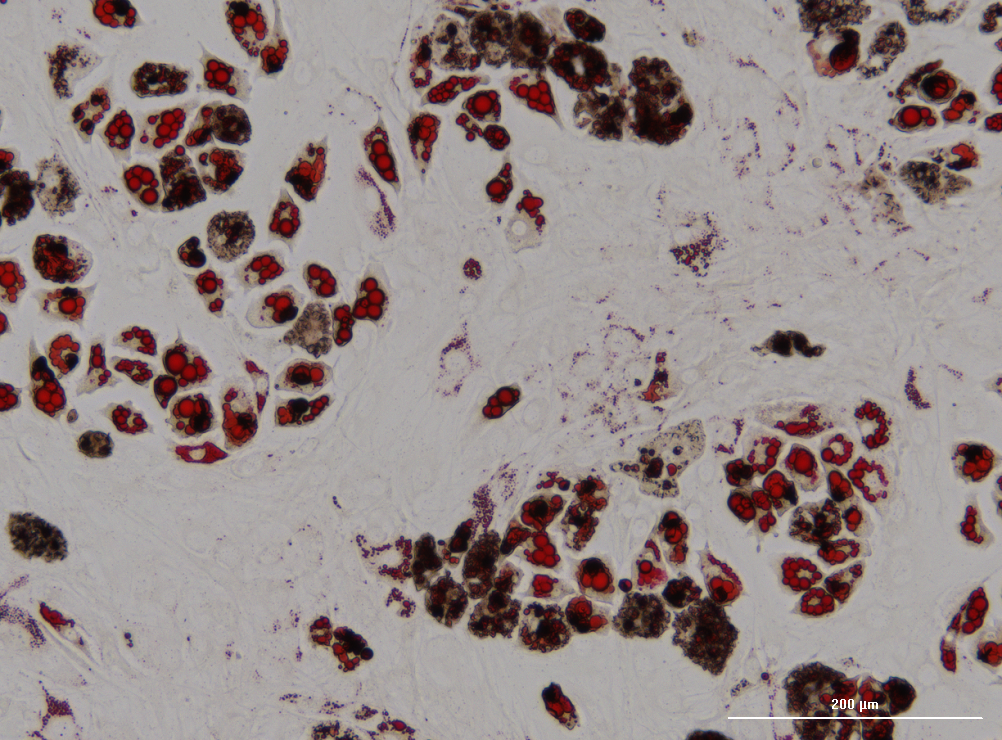

Supplement: Figure 5—figure supplement 1—source data 2. [file elife-74740-fig5-figsupp1-data2.zip › supplementary figure 9 D - source data/suppl fig 9D - source data 4 - well 3 shBrf1-2 10x.png]

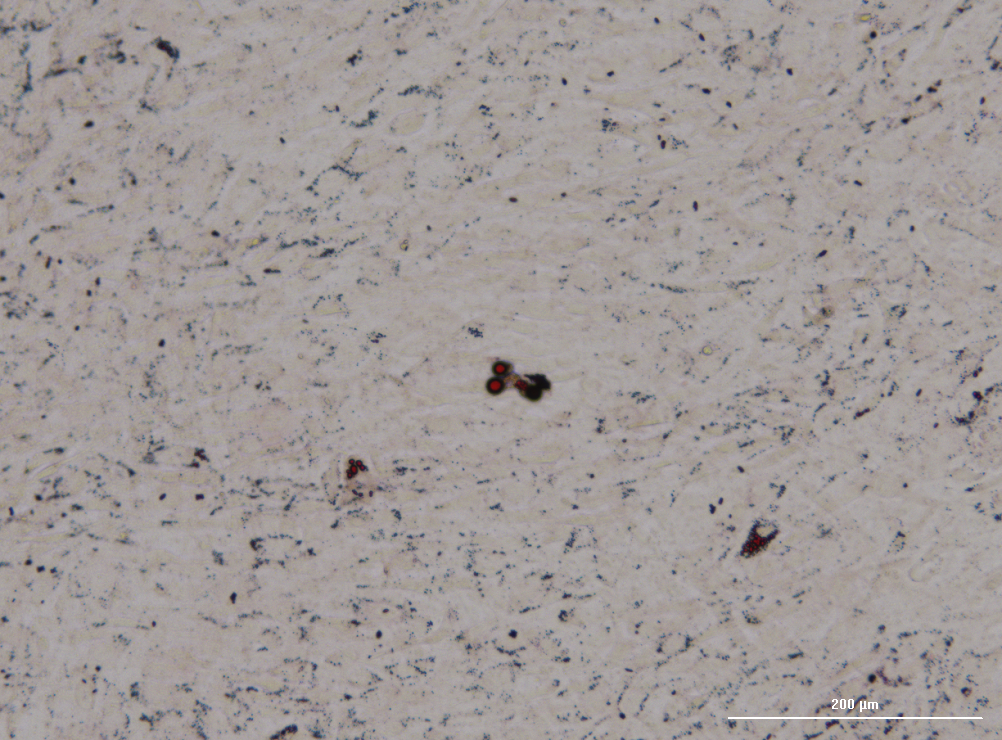

Supplement: Figure 5—figure supplement 1—source data 2. [file elife-74740-fig5-figsupp1-data2.zip › supplementary figure 9 D - source data/suppl fig 9D - source data 5 - well 4 SCR 10x.png]

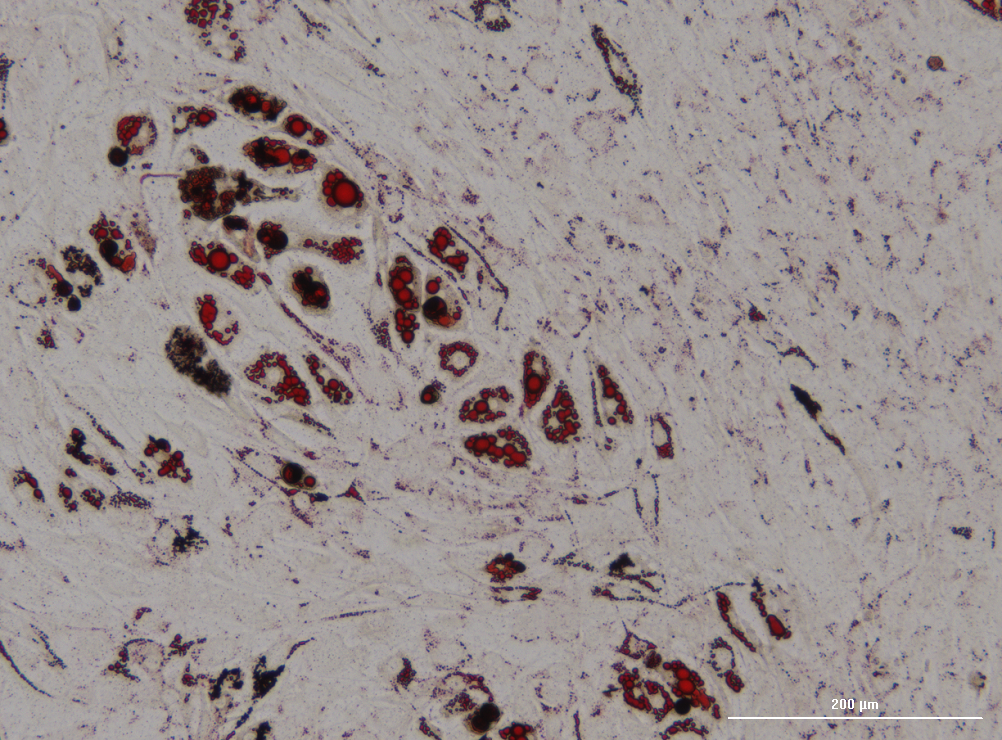

Supplement: Figure 5—figure supplement 1—source data 2. [file elife-74740-fig5-figsupp1-data2.zip › supplementary figure 9 D - source data/suppl fig 9D - source data 6 - well 5 shBrf1-1 10x.png]

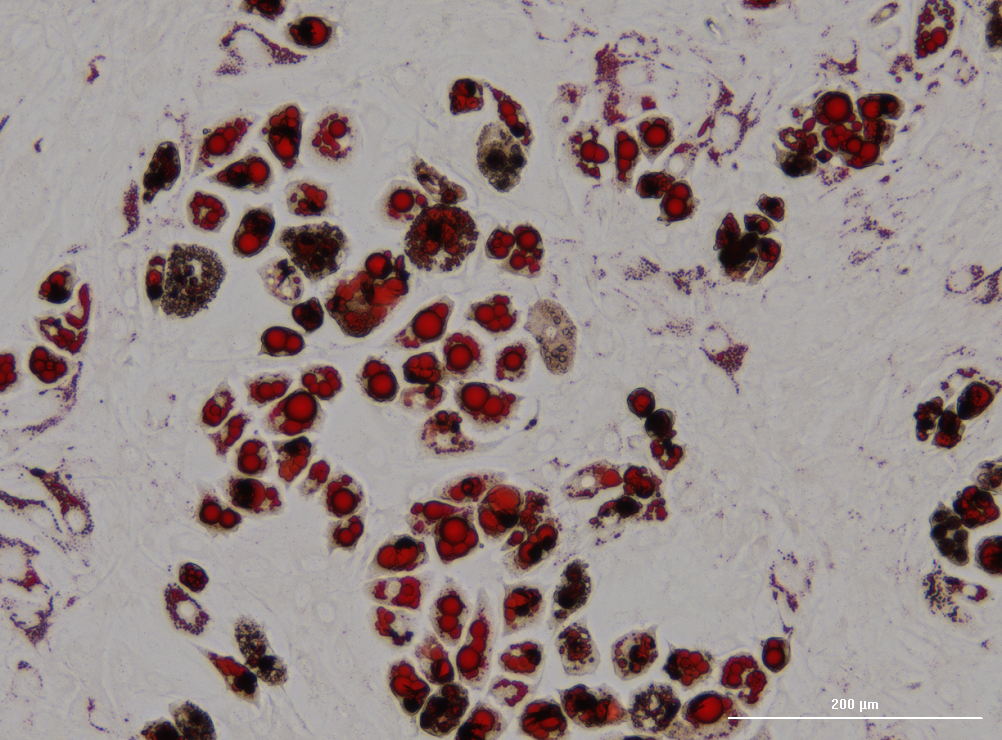

Supplement: Figure 5—figure supplement 1—source data 2. [file elife-74740-fig5-figsupp1-data2.zip › supplementary figure 9 D - source data/suppl fig 9D - source data 7 - well 6 shBrf1-2 10x.png]

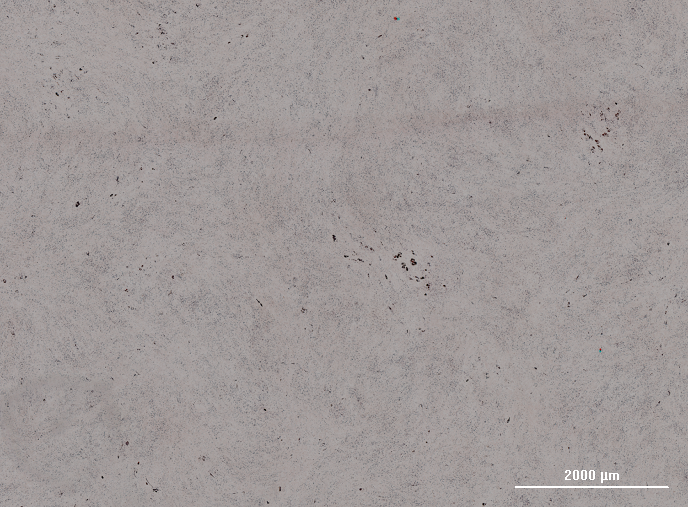

Supplement: Figure 5—figure supplement 1—source data 2. [file elife-74740-fig5-figsupp1-data2.zip › supplementary figure 9 D - source data/suppl fig 9D - source data 8 - well 1 SCR stitched.png]
