# Supplementary figures and images for: MAF1, a repressor of RNA polymerase III-dependent transcription, regulates bone mass (part 2 of 2)
Source: eLife. 2022 May 25;11:e74740. doi: 10.7554/eLife.74740 (PMC9212997; doi:10.7554/eLife.74740)

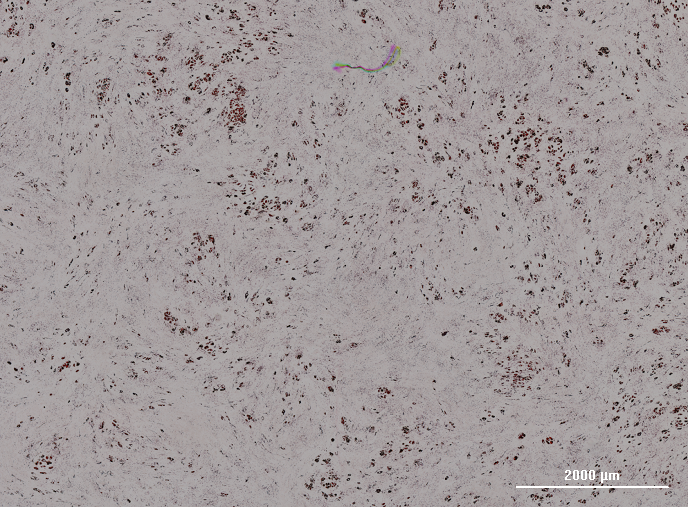

Supplement: Figure 5—figure supplement 1—source data 2. [file elife-74740-fig5-figsupp1-data2.zip › supplementary figure 9 D - source data/suppl fig 9D - source data 9 - well 2 shBrf1-1 stitched.png]
