# Supplementary material for: MAF1, a repressor of RNA polymerase III-dependent transcription, regulates bone mass
Source: eLife. 2022 May 25;11:e74740. doi: 10.7554/eLife.74740 (PMC9212997; doi:10.7554/eLife.74740)
Supplement: Supplementary file 1. [file elife-74740-supp1.docx]

**Supplementary File 1. qPCR primers used for genotyping and qRT-PCR analysis.**

| **Target** | **Forward primer** | **Reverse primer** |
| --- | --- | --- |
| Cre (genotyping) | TCCAATTTACTGACCGTACACCAA | CCTGATCCTGGCAATTTCGGCTA |
| LSL-Maf1 (genotyping) | TTCACTTCATACCCATACGACG | CCATTTTCCTTATTTGCCCCTA |
| WT Maf1 (21) (genotyping) | AGGCTTGCAGGGCAGCAATG | CACTGGCTGACAGGGAGATG |
| Maf1 KO (21) (genotyping) | AGGCTTGCAGGGCAGCAATG | TGGCCCTTAGAGCTGGAGTG |
| Pre-tRNA^Leu^ (29) | GTCAGGATGGCCGAGTGGTCTAAG | CCACGCCTCCATACGGAGAACCAGAAGACCC |
| Pre-tRNA_i_^Met^ (29) | CTGGGCCCATAACCCAGAG | TGGTAGCAGAGGATGGTTTC |
| Pre-tRNA^Ile^ (66) | GTTAGCGCGCGGTACTTATA | GGATCGAACTCACAACCTCG |
| Pre-tRNA^Pro^ (66) | GGCTCGTTGGTCTAGGG | TTTGAACCCGGGACCTC |
| Maf1(29) | GACTATGACTTCAGCACAGCC | CTGGGTTATAGCTGTAGATGTCAC |
| Brf1 (29) | GGAAAGGAATCAAGAGCACAGACCC | GTCCTCGGGTAAGATGCTTGCTT |
| Runx2 (67) | AGGGACTATGGCGTCAAACA | GGCTCACGTCGCTCATCTT |
| Col1α | CCCAATGGTGAGACGTGGAA | TTGGGTCCCTCGACTCCTAC |
| Sp7 (68) | ATGGCGTCCTCTCTGCTTG | GTCCATTGGTGCTTGAGAAGG |
| Osteocalcin (69) | TCTGACAAAGCCTTCATGTCC | AAATAGTGATACCGTAGATGCG |
| Alp | CGGATCCTGACCAAAAACC | TCATGATGTCCGTGGTCAAT |
| Bone sialoprotein | GAAAATGGAGACGGCGATAG | CATTGTTTTCCTCTTCGTTTGA |
| EF1α (70) | CTGAACCATCCAGGCCAAAT | GGCTGTGTGACAATCCAG |
| β-actin | CGACAACGGCTCCGGCATG | CTGGGGTGTTGAAGGTCTCAAACATG |
| RANK-L | CAGCCATTTGCACACCTCAC | GTCTGTAGGTACGCTTCCCG |
| OPG | AGGAACTGCAGTCCGTGAAG | ATTCCACACTTTTGCGTGGC |
| Ppia1 (29) | CGAGCTGTTTGCAGACAAAGTTCC | CCCTGGCACATGAATCCTGG |
| Pparγ(29) | ATCATCTACACGATGCTGGCCT | TGAGGAACTCCCTGGTCATGAATC |
| Pparγ2 | TCGCTGATGCACTGCCTATGA | GGAGAGGTCCACAGAGCTGAT |
| C/ebpα(29) | GAACAGCAACGAGTACCGGGTA | CCATGGCCTTGACCAAGGAG |
| Fabp4(29) | TGGGAACCTGGAAGCTTGTCT | TCGAATTCCACGCCCAGTTTGA |
